# Fullerene Complexation in a Hydrogen-Bonded Porphyrin Receptor via Induced-Fit: Cooperative Action of Tautomerization and C–H···π Interactions

**DOI:** 10.1021/jacs.2c10668

**Published:** 2022-12-22

**Authors:** Augustina Jozeliu̅naitė, Algirdas Neniškis, Arnau Bertran, Alice M. Bowen, Marilena Di Valentin, Steponas Raišys, Paulius Baronas, Karolis Kazlauskas, Linas Vilčiauskas, Edvinas Orentas

**Affiliations:** † Institute of Chemistry, Vilnius University, LT-03225 Vilnius, Lithuania; ‡ Centre for Advanced Electron Spin Resonance and Inorganic Chemistry Laboratory, Department of Chemistry, University of Oxford, OX1 3QR Oxford, United Kingdom; § Department of Chemistry, Photon Science Institute and The National EPR Research Facility, The University of Manchester, Manchester M13 9PL, United Kingdom; ∥ Department of Chemical Sciences, University of Padova, 35131 Padova, Italy; ⊥ Centro Interdipartimentale di Ricerca “Centro Studi di Economia e Tecnica dell’energia Giorgio Levi Cases”, 35131 Padova, Italy; # Institute of Photonics and Nanotechnology, Vilnius University, Saulėtekio av. 3, LT-10257 Vilnius, Lithuania; ∇ Center for Physical Sciences and Technology (FTMC), Saulėtekio al. 3, LT-10257 Vilnius, Lithuania

## Abstract

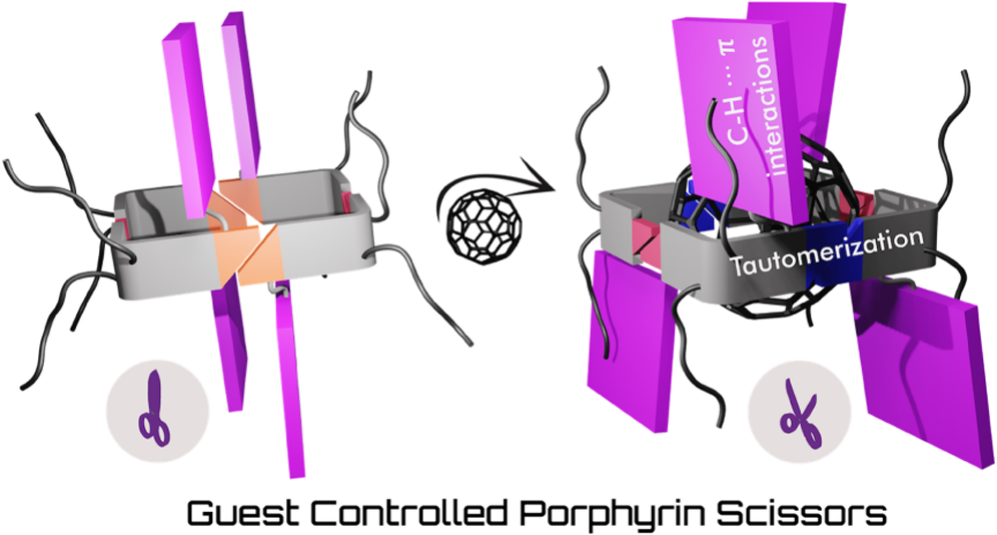

A supramolecular chiral hydrogen-bonded tetrameric aggregate possessing a large cavity and tetraarylporphyrin substituents was assembled using alternating 4H- and 2H-bonds between ureidopyrimidinone and isocytosine units, respectively. The aggregation mode was rationally shifted from social to narcissistic self-sorting by changing urea substituent size only. The H-bonded tetramer forms a strong complex with C_60_ guest, at the same time undergoing remarkable structural changes. Namely, the cavity adjusts to the guest via keto-to-enol tautomerization of the ureidopyrimidinone unit and as a result, porphyrin substituents move apart from each other in a scissor blade-like opening fashion. The rearrangement is accompanied by C–H···π interaction between the alkyl solubilizing groups and the nearby placed porphyrin π-systems. The latter interaction was found to be crucial for the guest complexation event, providing energetic compensation for otherwise costly tautomerization. We showed that only the systems possessing sufficiently long alkyl chains capable of interacting with a porphyrin ring are able to form a complex with C_60_. The structural rearrangement of the tetramer was quantitatively characterized by electron paramagnetic resonance pulsed dipolar spectroscopy measurements using photogenerated triplets of porphyrin and C_60_ as spin probes. Further exploring the C–H···π interaction as a decisive element for the C_60_ recognition, we investigated the guest-induced self-sorting phenomenon using scrambled tetramer assemblies composed of two types of monomers possessing alkyl chains of different lengths. The presence of the fullerene guest has enabled the selective scavenging of monomers capable of C–H···π interaction to form homo-tetrameric aggregates.

## Introduction

The modulation of the host–guest chemistry by means of extending beyond simple covalent adjustment of the cavity size represents a highly sought-after strategy to construct dynamic supramolecular receptors that are reminiscent of enzymes.^1−4^ In nature, induced-fit,^5,6^ conformational selection,^7,8^ and allosteric control,^9−11^ all operating by conformational changes of the host in response to the guest or other stimuli present, are the principles governing the substrate binding, activation, or remote control of the binding itself. Structural plasticity is also central to protein–protein recognition.^12^ Although abundant in biological systems, the induced fit still remains underexplored in artificial supramolecular systems.^13−29^ This can be partially attributed to the fact that the large part of the cavity is often assembled covalently to achieve shape persistency and high degree of preorganization. More dynamic aggregates composed of a larger number of smaller building blocks are intrinsically more difficult to design, especially those endowed with such challenging attributes as stimuli-responsive and conformationally flexible cavities. The host–guest properties of the majority of adaptive cavitands or capsules rely on adjusting the cavity interior using a rotation around the σ-bonds as part of the molecular framework.

The construction of large cavities reaching nanoscale dimensions using noncovalent synthesis is a formidable task that has usually been tackled by employing metal coordination chemistry.^30^ Less common are approaches based on hydrogen bonding (H-bonds), despite many obvious advantages of this directional interaction.^27−29,31,32^ Namely, switching H-bonds on and off is easily done by changing the polarity of the media or introducing competing molecular partners, whereas the spatial arrangement of assembly parts and the stability of the construct can be controlled by the judicious choice of the H-bonding arrays, number of H-bonds within these arrays, and their complementarity. Much less explored, yet a potentially very powerful way to control the assembly topology is by using different tautomeric forms of the H-bonding unit or secondary interactions formed between complementary dimers.^33^

In our report, we present a chiral H-bonded receptor capable of adjusting the cavity size by tautomeric changes in the H-bonding motif. Unlike the conformationally flexible systems, our tubular aggregate displays switching between two fully rigid cavities as a result of the conversion of the keto-form of Meijer’s ureidopyrimidinone (**UPy**)^34^ into the enol form upon complexation of C_60_. The obtained results also suggest that such a tautomeric switch constitutes an energetically uphill process, which is not fully compensated by noncovalent interactions between the guest and the host unless additional stabilization is provided from peripheral solubilizing alkyl chains.

**Figure 1:**
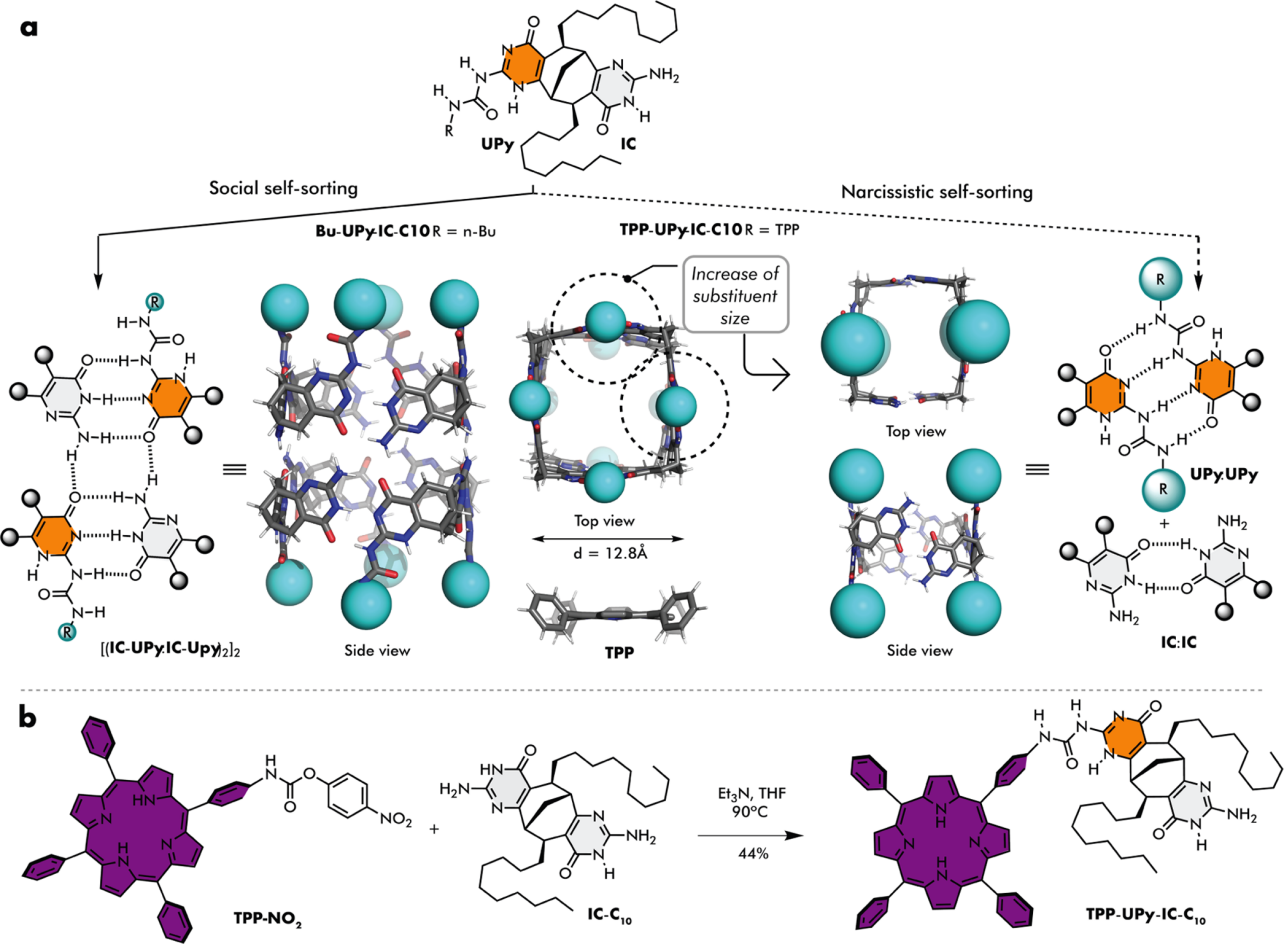
(a) Chemical structure of monomers and substituent size-modulated switching between social (left) and narcissistic (right) self-sorting of ureidopyrimidinone (**UPy**) and isocytosine (**IC**) H-bonding motifs. (b) Synthesis of tetraphenyl porphyrin functionalized monomer.

The molecular design of the receptor is based on the results of our previous study, where we have demonstrated that mixing of isocytosine (**IC**) and **UPy** units, embedded in a rigid and chiral bicyclo[3.3.1]nonane framework (monomer **Bu**-**UPy**-**IC**-**C**_**10**_), results in social self-sorting, forming hetero-dimers connected via triple H-bonds (Figure 1a, left).^33a^ Such unorthodox aggregation mode, where one H-bond donor of the **UPy** fragment is no longer involved in H-bonding, is stabilized by establishing a new cooperative H-bonding interface connecting two cyclic tetramers into an octameric tube (Figure 1a, left). The social self-sorting of **UPy** and **IC** motifs resulted in all eight urea substituents placed at the termini of the octameric supramolecular tube (cyano spheres in Figure 1a). An alternative tetrameric complex held together by narcissistically self-sorted alternating quadruple **UPy**:**UPy** and 2H-bonding **IC**:**IC** dimers was not observed (Figure 1a, right). Based on these findings, we became interested in whether it would be possible to have a control over the aggregation outcome by changing the bulkiness of the urea substituent of the **UPy** unit. Steric crowding of the four substituents at the tube termini was expected to favor the tetrameric aggregate with substituents arranged in an alternating fashion (Figure 1a, right). To test this idea, we designed a model system composed of the **TPP**-**UPy**-**IC**-**C**_10_ enantiopure monomer bearing tetraphenyl porphyrin (**TPP**) substituents and decyl solubilizing chains (**C**_10_) on the bicyclic scaffold (Figure 1b). The results of molecular modeling show the **TPP** unit to be slightly wider than the tube diameter. Therefore, it is impossible to have all four **TPP**s arranged parallel to the tube walls. The choice for the **TPP** substituent was additionally motivated by its pronounced spectroscopic features (*e*.*g*., intense Soret band) and further interesting light harvesting or sensing applications.^35−38^

## Results and Discussion

### Synthesis and Characterization

**Figure 2:**
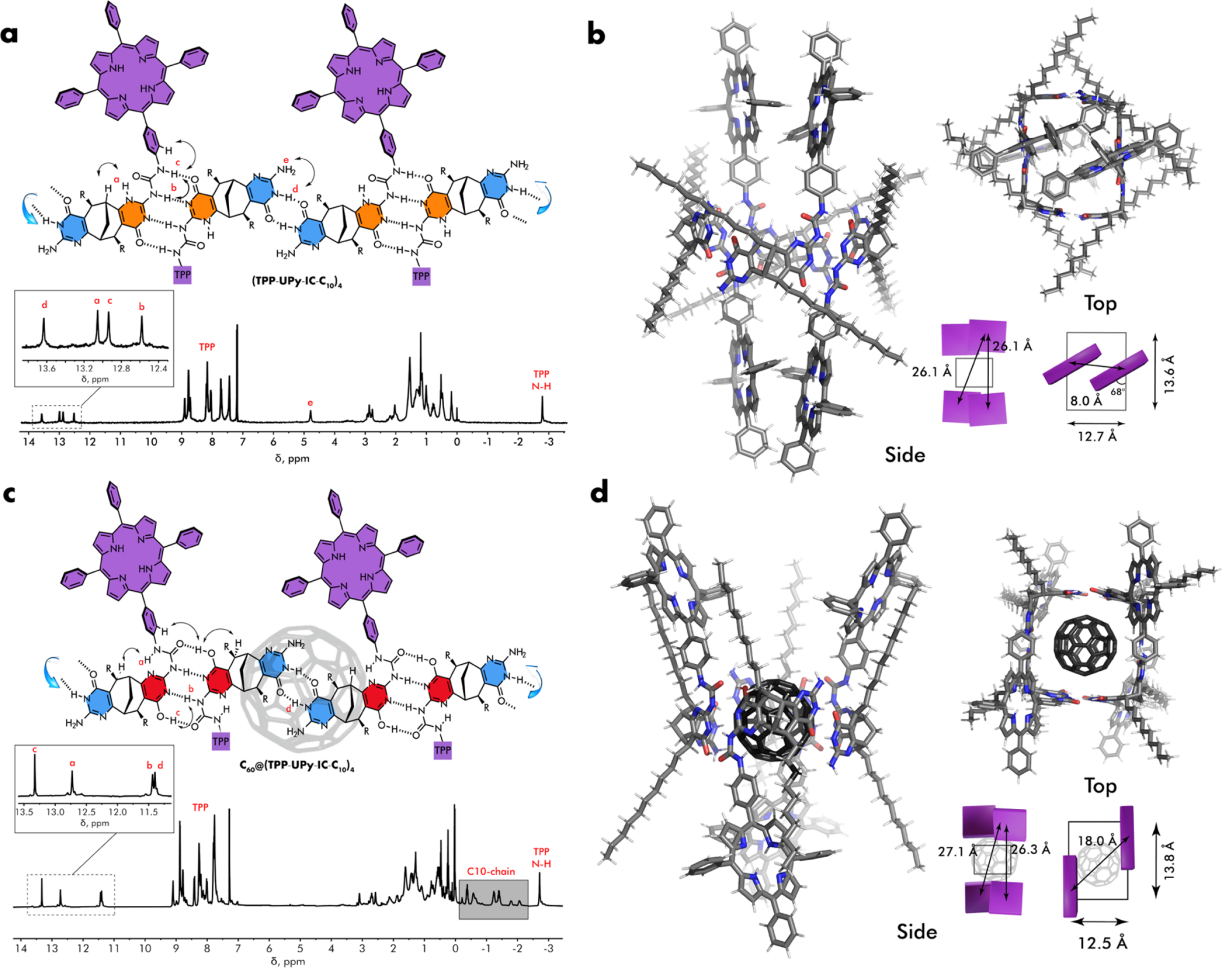
(a) Schematic representation of (**TPP**-**UPy**-**IC**-**C**_10_)_4_ tetramer and its ^1^H NMR spectrum with key resonances assigned. (b) Side (left) and top (right) views of the molecular model (molecular mechanics) of (**TPP**-**UPy**-**IC**-**C**_10_)_4_ tetramer with the dimensions of the cavity and interporphyrin distance indicated. (c) Schematic representation of the C_60_@(**TPP**-**UPy**-**IC**-**C**_10_)_4_ complex and its ^1^H NMR spectrum with key resonances assigned. (d) Side (left) and top (right) views of the molecular model (molecular mechanics) of the C_60_@(**TPP**-**UPy**-**IC**-**C**_10_)_4_ complex with the dimensions of the cavity and interporphyrin distance indicated.

The enantiopure monomer **TPP**-**UPy**-**IC**-**C**_10_ was synthesized in a single step from a known bis-isocytosine **IC**-**C**_10_^33a^ and 4-(10,15,20-triphenylporphyrin-5-yl)aniline p-nitrophenylcarbamate **TPP**-**NO**_2_ as an isocyanate surrogate in 44% yield (Figure 1b). The ^1^H NMR spectrum of **TPP**-**UPy**-**IC**-**C**_10_ in CDCl_3_ indicated exclusive formation of alternate 2H- and 4H-bonded tetrameric aggregates. This was evidenced by the set of four upfield signals assigned to **UPy**:**UPy** dimer and **IC:IC** dimer NH resonances (Figure 2a). The resonance of **IC** −NH_2_ group appears at 4.83 ppm, and these protons are clearly not involved in H-bonding, in accord to alternating bonding mode. The assignment of resonances was made using two-dimensional (2D) ^1^H NMR scalar and dipolar correlation spectroscopy, such as COSY and ROESY (Figures 2a, S27, and S28). The proton **d** shows a single NOE correlation with **IC** −NH_2_ and thus must reside on the **IC** ring. The assignment of the proton **a** was based on the observed NOE to the bridgehead proton of the bicyclic framework, whereas the proton **c** was easily identified by NOE cross-peak to the **TPP** phenyl ring. The NOE correlation between the protons **b** and **c** and the ^1^H–^15^N HSQC spectrum, showing that all downfield resonances reside on nitrogen atoms, further supported the AADD-DDAA H-bonding mode between **UPy** units.

Diffusion-ordered spectroscopy (DOSY) provided a size estimate of the aggregate (Figure S30). The hydrodynamic radius *R*_H_ = 1.96 nm is consistent with the tetrameric aggregate. According to molecular modeling, the H-bonded tetramer possesses a large cavity of approximately 13 Å in diameter and 5 Å in depth (Figure 2b). To avoid the steric clash of the opposing **TPP**s, the aniline nitrogen loses conjugation with the phenyl ring but remains H-bonded in the **UPy**-**UPy** dimer. As a result, **TPP** units are arranged in parallel to each other and are rotated approximately 68° with respect to the longer wall of the tetramer (Figure 2b).

Next, the complexation of C_60_ was attempted to obtain a supramolecular dyad composed of a porphyrin donor and a fullerene acceptor. Treating the tetramer (**TPP**-**UPy**-**IC**-**C**_10_)_4_ with 1.0 equiv of C_60_ in CDCl_3_, resulted in the formation of new species as evidenced by the notably changed ^1^H NMR spectrum (Figure 2c). The H-bonding N–H region undergoes changes upon complexation, all resonances being shifted upfield compared to the free host. The empty tetramer and the complex are in slow equilibrium, and a 4:1 complexation stoichiometry was directly determined by adding increasing amounts of C_60_ until the disappearance of the resonances attributed to the empty tetramer was observed (Figure S42). The fact that upon complexation the resonances of H-bonding motifs were most affected and that the obtained complex possessed high symmetry clearly indicate the location of the guest within the central cavity and not between the porphyrin rings. The DOSY measurements corroborate the tetrameric structure of the complex giving the value of the hydrodynamic radius (*R*_H_ = 2.13 nm) similar to that of an empty tetramer. The dilution of the solution of C_60_@(**TPP**-**UPy**-**IC**-**C**_10_)_4_ down to 10^–5^ M showed no signals of the free cage giving a rough estimate of the complex stability in the range of *K* = 10^5^–10^6^ M^–1^.

Unexpectedly, a careful inspection of the NMR data revealed the change of the tautomeric form of the **UPy** unit upon the complexation. Namely, the ^1^H–^15^N HSQC correlation spectroscopy indicated that all but one downfield resonance at 13.33 ppm (proton **c**, Figure 2c) give the corresponding cross-peaks. Moreover, the same proton **c** shows NOE correlations with the protons on the aromatic ring and the bicyclic core as expected for the enolic form. Interestingly, the proton resonances of the isocytosine NH_2_ group become too broad to be visible in the ^1^H NMR spectrum.

The current system represents the first example of a tetrameric H-bonded **UPy**-based tetramer of this type where the enolic form of **UPy** is operating. For instance, the previously reported *C*_2_-symmetric bicyclic analogue **UPy**-**UPy**^31f^ only forms DDAA-AADD quadruple H-bonds even in aromatic solvents, where simple monotopic **UPy** derivatives show a large fraction of the enolic form.^34^ The reason behind such selectivity is most likely related to geometric factors. Namely, the movement of the urea substituent toward the bicyclic core in the enolic form places it nearby the solubilizing chain, leading to repulsive steric interactions. The combination of **TPP** and decyl substituents in the present system seems to violate this principle, despite the large size of **TPP**. The special role of solubilizing chain, however, was revealed by the observation that some hydrogen atoms of one of the two decyl chains in complex C_60_@(**TPP**-**UPy**-**IC**-**C**_10_)_4_ are highly shielded as evidenced by their upfield chemical shifts (δ = −0.31 to −2.19 ppm) in the ^1^H NMR spectrum (Figure 2c). This clearly implies a short distance between the alkyl chain and the porphyrin ring.^39,40^ The molecular modeling corroborated this assumption and showed that upon C_60_ complexation and keto-to-enol tautomerization of **UPy**, the **TPP** rings rotate and move toward the bicyclic core. At the same time, the nearest alkyl chain becomes stuck to the **TPP** surface presumably via C–H···π interactions. As a result, the distance between porphyrin centers increases from 0.8 to 1.8 nm (Figure 2d).^41^

**Figure 3:**
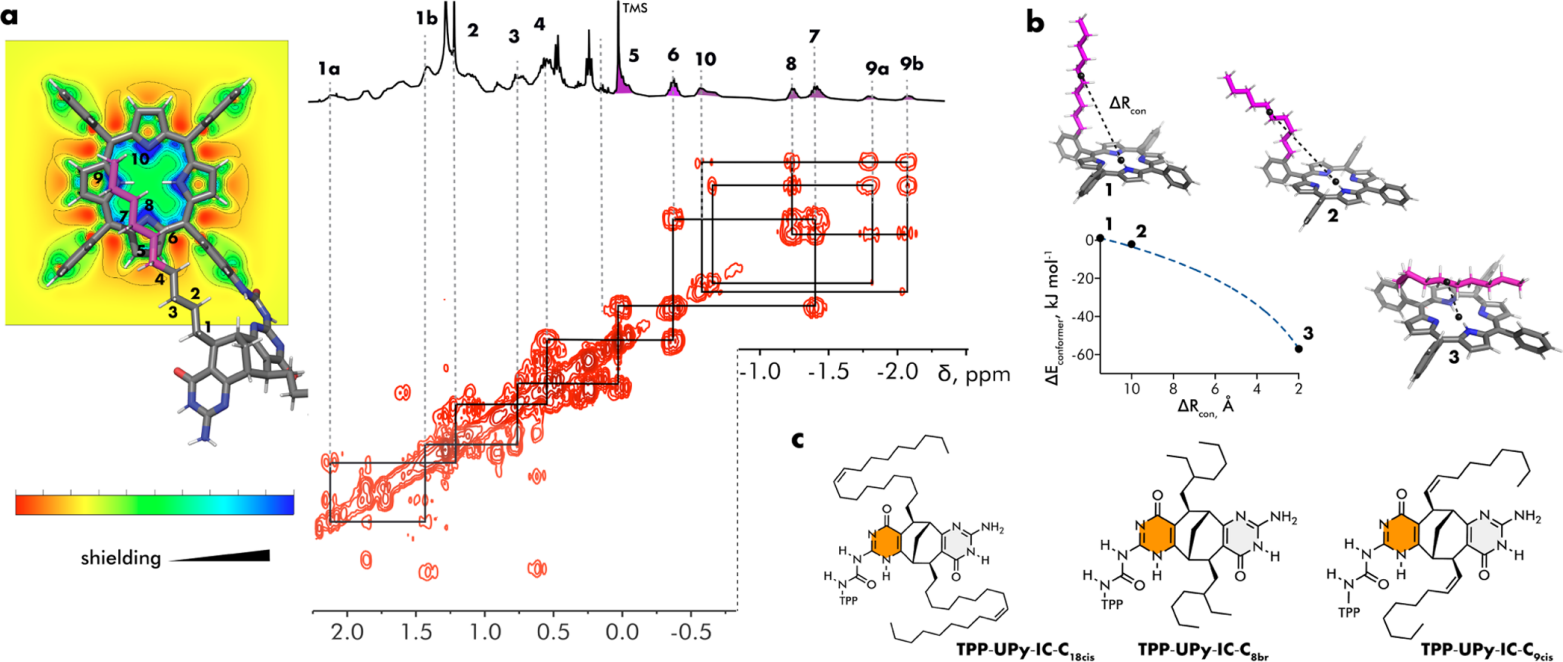
(a) Monomeric unit of the C_60_@(**TPP**-**UPy**-**IC**-**C**_10_)_4_ assembly highlighting the part of the alkyl chain involved in the C–H···π interaction (labeled purple) and the excerpt of the COSY spectrum. The **TPP** unit of the monomer is projected onto the NICS (1)*_zz_* value map at a 1.0 Å distance above the ring. (b) Selected computed conformations of the model system and the corresponding energy–distance plot. (c) Chemical structures of monomers possessing various types of solubilizing chains.

A closer look at the shielded part of the aliphatic chain in the COSY spectrum allowed the assignment of the resonances of the shielded chain (Figure 3a). The most shielded CH_2_ hydrogens are residing on the penultimate carbon atom (C9), located exactly on top of the pyrrole ring. The computed nucleus-independent chemical shifts (NICS)^42^ map of the aromatic **TPP** system also confirmed the location of the C9 methylene group over the most negative region corresponding to the strongest shielding. The C10 terminus is significantly less shielded as a result of its proximity to a more positive NICS region of the **TPP** system and also, due to partial deshielding by the orthogonal phenyl ring. Additionally, in accord to the NICS map, the C8 methylene group is less shielded than the neighboring C9 and C7 methylene groups, indicating a zig-zag conformation of the alkyl chain. Furthermore, the fact that six methylene groups are strongly shielded by the **TPP** aromatic system also indirectly confirms the fully extended conformation of the decyl chain.

Besides C_60_, the host–guest experiments with C_120_ (*i.e*. dimer of C_60_) and (**TPP**-**Upy**-**IC**-**C**_10_)_4_ indicated no complex formation, whereas the C_70_ guest provided an inclusion complex in CDCl_3_ with the characteristic NMR signature of the C–H···π interaction (Figure S43).

The generality of the herein observed C–H···π interaction between the aliphatic chain and the porphyrin π-system was also probed computationally. The truncated model comprising the **TPP** fragment with the decyl chain attached to the ortho position of one of the phenyl rings was subjected to conformational energy calculations using the B3LYP exchange–correlation functional together with the D3 version of Grimme’s dispersion and Becke-Johnson damping.^43,44^ The distance between the centers of mass of the porphyrin ring and the alkyl chain, *R*_con_, was used as a geometry parameter and plotted against the conformer energy (Figure 3b). A clear trend toward smaller *R*_con_ was observed during the conformational screening indicating a preference for an attractive interaction between the π-system and the alkyl chain. The lowest energy conformation found is characterized by a fully extended chain located on top of the porphyrin ring with *R* = 2.0 Å for the geometric parameter. The overall energy gain compared to the highest energy conformer with noninteracting chain is about 57 kJ/mol, which translates into 11 kJ/mol for one methylene group considering that five carbon atoms are in contact with the π system. Our findings are of the same order as the values found in other theoretical studies, for example, indicating the optimal distance *R* = 2.7 Å and energy *E* = 6.1 kJ/mol for the C–H (CH_4_)···π (benzene) interaction.^45^

The C–H···π interaction involving porphyrin π-donor has long been postulated to be relevant in porphyrin-containing proteins. The extensive screening of the Protein Data Bank revealed that all porphyrin rings are involved in X–H···π interactions.^46^ Among them, C–H···π interactions with the amino acid side chain C–H donors are the most prevailing, suggesting the importance of these weekly polar dispersive interactions for hemoprotein stability. To the best of our knowledge, the unambiguous evidence for the C–H···π interaction on simple porphyrin derivatives has never previously been reported, except for the solid-state structure of the highly preorganized derivative.^47^ Our system thus represents the first example of directly observable C–H···π interactions of a flexible, nonstrapped alkyl chain.

The next important question that arises is whether the observed C–H···π interaction is a necessary prerequisite for the formation of an insertion complex or is it merely a consequence of favorable geometry where the alkyl chain is brought in close contact to the porphyrin surface after **UPy** enolization. To address this issue, a series of monomers **TPP**-**UPy**-**IC**-**C**_n_ having aliphatic chains C_n_ of different lengths and shapes were synthesized (Figure 3c; for synthetic details, see the Supporting Information). Among them, there were derivatives possessing long bent oleyl (C_18cis_) and 2-*cis*-nonenyl (C_9cis_) or branched 2-ethylhexyl (C_8br_) chains. The latter compound was obtained as a mixture of diastereomers because of the racemic chain used. Unfortunately, the derivatives having shorter (<C_8_) linear alkyl chains were not soluble in chloroform.

Remarkably, the complexation experiment with C_60_ guest in CDCl_3_ showed the formation of the corresponding inclusion complex only for monomers with the oleyl chain (C_18cis_), whereas the other two derivatives remained unchanged even after prolonged heating with C_60_. The results of molecular modeling indicated that the oleyl chain, despite its bent form, can reach the porphyrin π system and engage in C–H···π interaction. Indeed, the ^1^H NMR spectrum displayed an identical fingerprint with highly shielded CH_2_ resonances and the formation of the **UPy** enol form (Figures S38 and S39). On the other hand, when the bending point of a cis-double bond is introduced next to the bicyclic scaffold, as in monomer **TPP**-**UPy**-**IC**-**C**_9cis_, none of the chain conformations is able to provide the C–H···π contact. Likewise, a 2-ethylhexyl chain is too short to reach the porphyrin ring. Altogether, these results demonstrate that **UPy** enolization and C–H···π interactions are acting cooperatively. Therefore, the enolization of the **UPy** motifs is required to provide an optimal cavity space to fit a C_60_ guest, but the energetic cost of tautomerization is not fully compensated by the complexation itself.

### Self-Sorting Experiments

**Figure 4:**
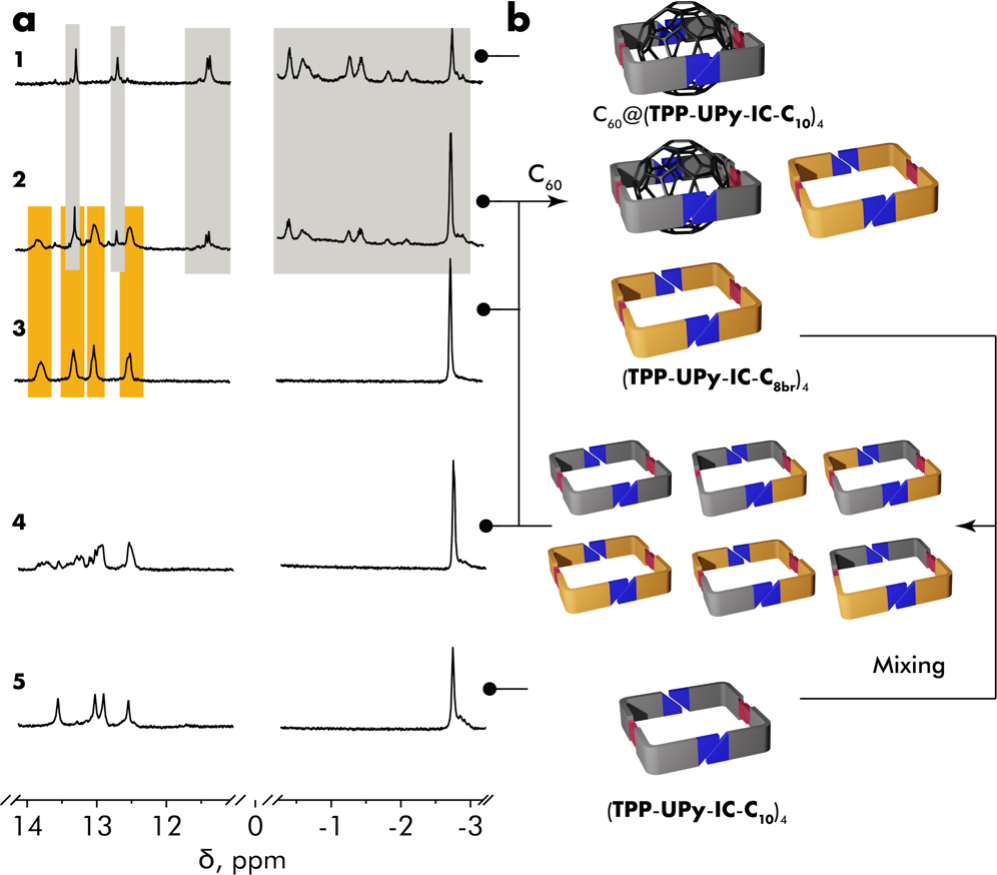
(a) ^1^H NMR spectra of the scrambled and guest-resolved mixture of monomers **TPP**-**UPy**-**IC**-**C**_10_ and **TPP**-**UPy**-**IC**-**C**_8br_. (b) Schematic representation of all species involved in the equilibrium.

The fact that C–H···π interactions are required for the inclusion complex with C_60_ to form implies that in a mixture of two monomers with different solubilizing chain lengths, the C_60_ guest should scavenge monomers with the longest chains to establish the most stable complex with a maximum number of favorable C–H···π interactions. To test this idea, we first performed a mixing experiment with equimolar amounts of **TPP**-**UPy**-**IC**-**C**_10_ and **TPP**-**UPy**-**IC**-**C**_8br_ monomers in CDCl_3_. Due to complementarity and identical monomer geometry, this combination is expected to deliver the statistical mixture of scrambled tetrameric aggregates. Because the **TPP**-**UPy**-**IC**-**C**_8br_ monomer is obtained as a mixture of diastereomers due to the chirality of the side chain, the N–H resonances in this case are broader than in **TPP**-**UPy**-**IC**-**C**_10_ (Figure 4, spectrum 3). Fortunately, the ^1^H NMR spectra of individual pure components give nonoverlapping N–H resonances allowing for easy identification of the corresponding homo-tetramers (Figure 4, spectra 3 and 5). The mixing of two monomers indeed led to a formation of a complex mixture of tetrameric aggregates as indicated by the appearance of a number of new N–H resonances (Figure 4, spectrum 4).

The addition of 0.125 equiv of C_60_ (with respect to the total amount of monomers) into the above mixture of scrambled tetramers resulted in a virtually full sorting into homo-tetramer (**TPP**-**UPy**-**IC**-**C**_8br_)_4_ and complex C_60_@(**TPP**-**UPy**-**IC**-**C**_10_)_4_ (Figure 4, spectrum 2). Such guest-induced sorting is unique in a sense that the only sorting controlling feature of the monomer is the length of the peripheral alkyl chain.^48^

### Circular Dichroism and Electron Paramagnetic Resonance Studies

To corroborate the molecular modeling results, the relative movement of porphyrin rings upon complex formation was probed qualitatively using circular dichroism (CD) spectroscopy and quantitatively by electron paramagnetic resonance pulsed dipolar spectroscopy (EPR-PDS).

The chirality of the bicyclic backbone, the excellent photophysical properties of the porphyrin, and the presence of multiple chromophores within the supramolecular aggregate renders our system suitable for the exciton-coupled circular dichroic method.^49,50^ The interaction between the excited states of chromophores in chiral environments gives rise to bisignate CD curves, i.e., exciton couplet, the sign and shapes of which are determined by the absolute skewness of interacting chromophores.^51^

**Figure 5:**
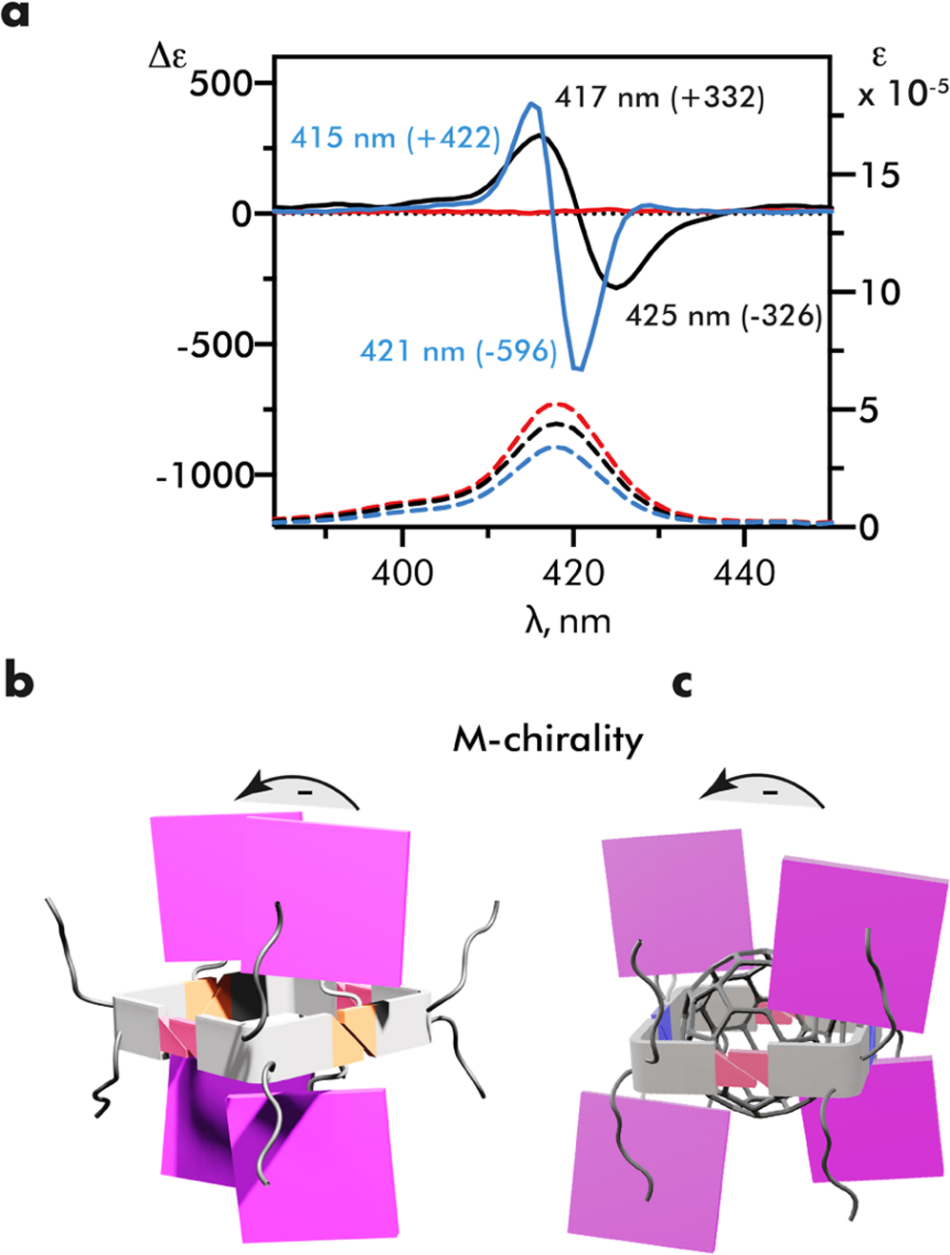
(a) Visible-region (Soret band) of CD and UV–vis spectra of tetramer (**TPP**-**UPy**-**IC**-**C**_10_)_4_ (black line) and complex C_60_@(**TPP**-**UPy**-**IC**-**C**_10_)_4_ (blue line) in CDCl_3_ (*c* = 6.0 μM). CD and UV–vis spectra of monomeric **TPP**-**UPy**-**IC**-**C**_10_ in DMSO are shown as a red line. Schematic representation of exciton chirality in empty tetramers (b) and complexes (c).

The CD spectra in the visible range were recorded for a chloroform solution of tetramer (**TPP**-**UPy**-**IC**-**C**_10_)_4_ capable of complexing a C_60_ guest. The strong negative exciton couplet is observed at the Soret band (λ_max_ = 424 nm) (Figure 5a). The negative sign of the couplet predicted from the molecular model was in agreement with the exciton chirality model (Figure 5b). Although the diagonal pairs of **TPP**s should also give the exciton couplet of the same sign, their contribution is expected to be smaller due to a longer distance. No CD band was observed in the Soret region in a competing DMSO solvent, indicating that the chirality of the bicyclic backbone is not sensed by **TPP** in the monomeric **TPP**-**UPy**-**IC**-**C**_10_.

The corresponding C_60_@**TPP**-**UPy**-**IC**-**C**_10_ complex was prepared from stoichiometric amounts of components. The UV–vis spectra showed no shift of the Soret and Q-bands upon complexation supporting a binding at the central cavity, distant from porphyrin rings. On the other hand, the CD spectrum was responsive to complex formation showing a notable increase of the couplet intensity (Figure 5a,c). In addition, both positive and negative CD bands in the C_60_@**TPP**-**UPy**-**IC**-**C**_**10**_ spectrum display the hypsochromic shift with respect to **TPP**-**UPy**-**IC**-**C**_**10**_. These spectral changes are indicative of the movement of **TPP** rings upon complexation; however, the exact geometry of the complex cannot be reliably estimated. The exciton coupling strength depends on the relative geometry in a nontrivial fashion with some calculations and experimental work suggesting a coupling maximum at 50–60°.^52−55^ Possibly, the increase of the distance between porphyrin rings in a complex is compensated by a stronger dipole exchange in more twisted geometries.^54^

The results of CD spectroscopy supported the insights obtained from molecular modeling regarding the rearrangement of the tetramer, however, only qualitatively. EPR-PDS measurements were thus conducted to quantitatively study the distances between the porphyrin centers in the system with and without the C_60_ guest. EPR-PDS is a suite of techniques that are used to measure the dipolar interactions between moieties containing unpaired electrons separated by distances of ca. 1.5 to 8+ nm; the strength of the measured dipolar interaction can be interpreted to gain information about the distance and the distance distribution, and for some rigid system, the angles between the centers containing unpaired electrons.^56,57^ The double electron–electron resonance (DEER)^58^ can be used to measure systems containing two or more permanent paramagnetic spin centers and light-induced triplet–triplet electron resonance (LITTER)^59^ can be used to measure dipolar interactions between two chromophores with photogenerated triplet states. In the case of C_60_@(**TPP**-**UPy**-**IC**-**C**_10_)_4_, LITTER was used to measure the dipolar interaction between light-induced spin-active triplet states of the free-base porphyrin chromophores.^59^

**Figure 6:**
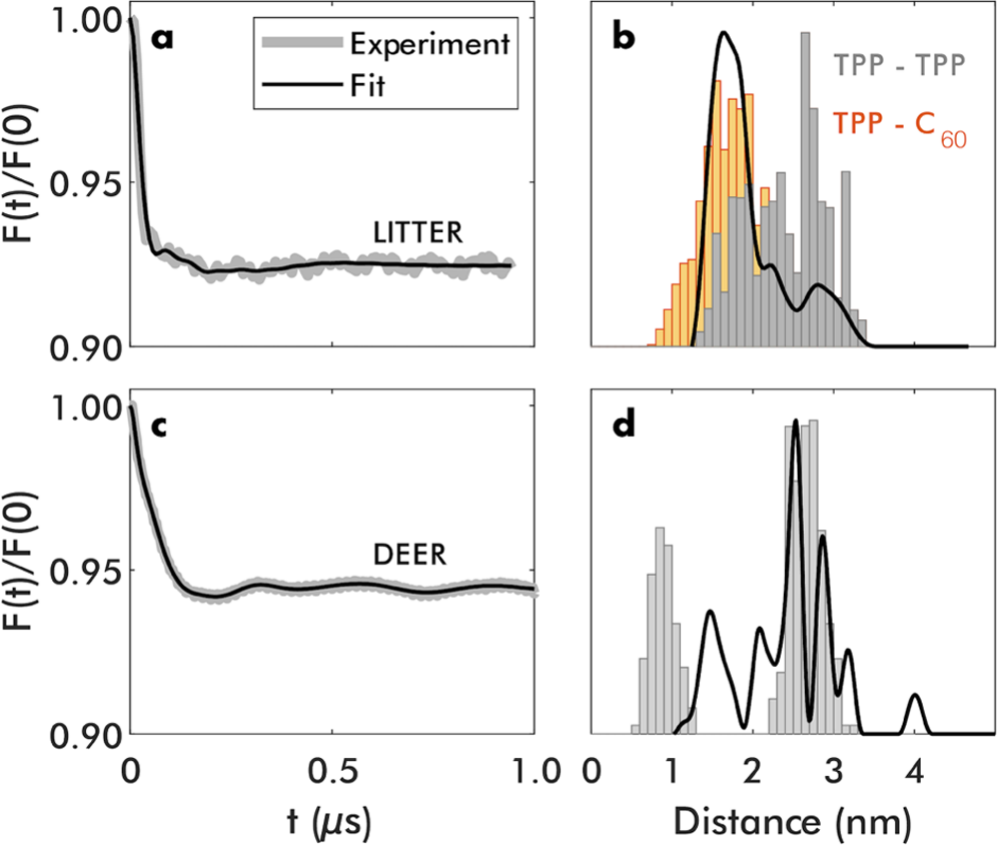
Background-corrected EPR-PDS traces (gray) and fits (black) for LITTER measured on C_60_@(**TPP**-**UPy**-**IC**-**C**_10_)_4_ (a) and DEER measured (**TPP**-**UPy**-**IC**-**C**_10_)_4_ (c). Corresponding spin–spin distance distributions obtained from the fits (black lines) and expected distances from the molecular models (colored bars) for (b) LITTER and (d) DEER. Individual distances in the histograms have been weighted by the electron spin densities on the involved atoms, as determined from DFT calculations. The contributions from **TPP**-**TPP** and **TPP**-C_60_ distances to each bar have been indicated separately in panel (b) in gray and ocher, respectively, weighted by the corresponding extinction coefficients and triplet quantum yields. Histograms and distance distributions are normalized to their absolute maximum.

The orientation-averaged LITTER trace (Figure 6a) and the corresponding distance distribution (Figure 6b; for interporphyrin distances, see Figure 2), extracted using a modified DeerAnalysis algorithm^60^ (see Supporting Information for form details), show distances in the same range as predicted from the molecular model of this system. The excitation of the system with C_60_ induced the formation of triplet states on both the porphyrins and the C_60_ (Figure S45). Therefore, in the LITTER trace analysis, it is expected to see both distances, i.e., between the two porphyrin moieties and between the porphyrin and the C_60_. These distances predicted by the model are plotted in Figure 6b in gray and ocher, respectively. While the distance ranges observed experimentally are a good match to the expected model, deviations in the expected intensity across this distance range may be a result of different phase memory times (*T*_m_) of the porphyrin triplet state used for detection in the presence and absence of other triplet state moieties, and a function of the trace length used; shorter distances are often relatively amplified when the recorded trace is short.^61^

In the other case of a system without bound C_60_, the *T*_m_ of the porphyrin triplet state was so short that it was not possible to detect a spin-echo signal, prohibiting the use of LITTER. Instead, this system was metalated with Cu(II), and DEER was used to measure the interspin distances between the Cu(II) ions coordinated into the porphyrins.^58^ The distance distribution resulting from the orientationally averaged DEER trace and the obtained distance distribution (Figure 6c,d) are in good agreement with that predicted from the model (gray bars) in the region above 2.0 nm. The model predicts an additional short distance centered at 0.8 nm due to the effective stacking of porphyrin moieties. DEER has a lower detection limit of ca. 1.5 nm.^62^ It is likely that the experimentally determined distances between 1.0 and 2.0 nm are a result of a partial detection of the frequency contributions corresponding to distances <1.0 nm predicted from the model. In this way, the observed EPR-PDS qualitatively and quantitatively corroborates the proposed model of the system.

### Photophysical Studies

The unique geometry of the supramolecular pentad C_60_@(**TPP**-**UPy**-**IC**-**C**_10_)_4_ prompted us to further investigate potential interchromophore interactions and to gain more insight into energy or electron transfer processes characteristic to such systems.^63^ The majority of porphyrin–fullerene systems reported so far are built either by appending the fullerene derivatives to metalated porphyrin using pyridine–metal coordination bond,^63^ covalently linking chromophores^64^ or complexing the fullerene in between porphyrin rings.^65^ Few studies have also been reported on H-bonded porphyrin–fullerene dimers.^66^ One dyad with remotely bound C_60_ assembled using the van der Waals interaction has also been characterized.^67^

The photophysical characterization of the tetramer was performed using chloroform and 1,2-dichlorobenzene solution in which the formation of the inclusion complex with C_60_ was unambiguously confirmed by NMR (Figure S49). The fluorescence yields of the empty tetramer Φ_F_ = 5.9% in chloroform were similar to the one of the complex, Φ_F_ = 4.4%, corresponding well to a typical value of the parent **TPP**.^68^ This strongly suggests the absence of energy or electron transfer in the system. Similar results were obtained for 1,2-dichlorobenzene solution. Fluorescence transients were almost identical for both (**TPP**-**UPy**-**IC**-**C**_10_)_4_ and C_60_@(**TPP**-**UPy**-**IC**-**C**_10_)_4_, displaying monoexponential decay with lifetimes of τ = 9.3 ns and τ = 8.2 ns and τ = 8.1 ns and τ = 8.0 ns in 1,2-dichlorobenzene and chloroform, respectively (Figures S50 and S51). Transient absorption spectroscopy further provided evidence for the absence of electronic communication between **TPP** and C_60_ chromophores in chloroform showing identical time-absorption profiles for both species and no spectral signatures of **TPP**-C_60_^1^ or **TPP**^•+^-C_60_^•–^ (Figures S53 and S54). Similar results were also obtained for the C_70_@(**TPP**-**UPy**-**IC**-**C**_10_)_4_ complex in CDCl_3_.

In light of a wide range of geometries and distances that have been used to obtain efficient porphyrin–fullerene dyads, the lack of chromophore communication in our system is very unusual. Further studies will be required to explain this phenomenon. Yet, such photosilent fullerene switches can find use in designing responsive dyads where the interaction of two chromophores is turned on upon C_60_ complexation.

## Conclusions

We have demonstrated the first example of the host–guest system based on a dynamic H-bonded receptor operating by a cooperative action of tautomerization and C–H···π interactions. The tetramer assembled by sterically induced homodimerization of 2H- and 4H-bonding isocytosine and ureidopyrimidinone units, respectively, exhibits cavity plasticity toward the C_60_ guest via tautomerization. The accommodation of the guest, however, requires compensation of otherwise energetically costly tautomerization and is only occurring in the monomers equipped with sufficiently long solubilizing chains, capable of establishing C–H···π contacts with the porphyrin surface. Our system represents an interesting case of dynamic supramolecular assemblies where peripheral C–H···π interactions, otherwise regarded as very weak, have a decisive role in guiding the guest-induced rearrangement process, which results in a movement of porphyrin rings by a distance of 1.0 nm. The tautomerization and corresponding geometrical changes in the system were studied and corroborated using both CD and EPR-PDS measurements. The latter technique provided quantitative results on the porphyrin–porphyrin and, for the first time, porphyrin–fullerene distances. Remarkably, despite the close distance between the porphyrin ring and the C_60_ guest, no energy or electron transfer between these chromophores was observed.

The strong complexation of the C_60_ guest enabled by C–H···π interactions was successfully explored for the purpose of separating the mixture of scrambled hetero-tetrameric aggregates into homo-tetramers based only on the length of the solubilizing chain.

The herein-reported principle to control host–guest chemistry can be implemented in the future allosteric systems with photoresponsive side chains to enable light modulation or dynamic covalent side chains for constructing chemically triggered receptors. From a fundamental standpoint, the induced fit operating through C–H···π interactions is offering new ways to investigate this important noncovalent interaction and studies aiming to extend this principle to π-systems beyond porphyrin are currently ongoing.

## Data Availability

Data supporting this study are provided as supporting information accompanying this paper and available on request from the authors.

## Supporting Information Available

Experimental procedures and copies of NMR spectra and molecular models of the assemblies (PDF)
