# Supplementary material for: Fullerene Complexation in a Hydrogen-Bonded Porphyrin Receptor via Induced-Fit: Cooperative Action of Tautomerization and C–H···π Interactions
Source: J Am Chem Soc. 2022 Dec 22;145(1):455–64. doi: 10.1021/jacs.2c10668 (PMC9837862; doi:10.1021/jacs.2c10668)
Supplement: Supplementary file 1 — ja2c10668_si_001.pdf [file ja2c10668_si_001.pdf]

Supporting Information

Fullerene Complexation in a Hydrogen-Bonded Porphyrin Receptor via Induced-Fit: Cooperative Action of Tautomerization and C-H...π Interactions

Augustina Jozeliūnaitė[a], Algirdas Neniškis[a], Arnau Bertran[b], Alice M. Bowen[c], Marilena di Valentin[d], Steponas Raišys[e], Paulius Baronas[e], Karolis Kazlauskas[e], Linas Vilčiauskas[a,f] and Edvinas Orentas[a]*

[a] Institute of Chemistry, Vilnius University, LT-03225 Vilnius, Lithuania

[b] Centre for Advanced Electron Spin Resonance and Inorganic Chemistry Laboratory, Department of Chemistry, University of Oxford, OX1 3QR, Oxford, United Kingdom

[c] Department of Chemistry, Photon Science Institute and The National EPR Research Facility, The University of Manchester, Manchester M13 9PL, United Kingdom

[d] Department of Chemical Sciences, University of Padova, 35131 Padova, Italy; Centro Interdipartimentale di Ricerca “Centro Studi di Economia e Tecnica dell’energia Giorgio Levi Cases”, 35131 Padova, Italy

[e] Institute of Photonics and Nanotechnology, Vilnius University, Saulėtekio av. 3, LT-10257 Vilnius, Lithuania

[f] Center for Physical Sciences and Technology (FTMC), Saulėtekio al. 3, LT-10257 Vilnius, Lithuania

E-mail: edvinas.orentas@chf.vu.lt

General experimental

Synthesis and characterization. Reagents were purchased at the highest commercial quality and used without further purification. Dry tetrahydrofuran was distilled from sodium/benzophenone ketyl, dry dichloromethane, pyridine and triethylamine were distilled from CaH_2. Yields refer to chromatographically and spectroscopically homogeneous material. Reactions were monitored by thin layer chromatography (TLC) carried out on 0.25 mm Merck silica plates ($60F_{254}$), using UV light as the visualizing agent and/or vanillin and heat as a developing agent. Flash silica gel chromatography was performed using Fluorochem or Merck silica gel (60, particle size 0.043 – 0.063 mm). NMR spectra were recorded on Bruker DRX400 instruments in $CDCl_3$ (unless stated otherwise) and were calibrated using residual undeuterated solvent as an internal reference (d-chloroform: ^{1}H NMR δ = 7.26 ppm, ^{13}C NMR δ = 77.16 ppm, d_6-DMSO ^{1}H NMR δ = 2.50 ppm, ^{13}C NMR δ = 39.52 ppm). The following abbreviations were used to explain NMR peak multiplicities: s = singlet, d = doublet, t = triplet, q = quartet, m = multiplet, br = broad and combinations thereof. High resolution mass spectra (HRMS) were recorded on an Agilent LC/MSD TOF mass spectrometer by electrospray ionization time-of-flight (ESI-TOF) reflectron experiments.

Diffusion ordered NMR spectroscopy (DOSY). DOSY experiments were performed on a 400 MHz Bruker Avance NMR spectrometer equipped with an Accustar z-axis gradient amplifier and an ATMA BBO probe with a z-axis gradient coil. All experiments were run using insert tubes and without spinning to avoid convection. All calculations were performed using standard applications in Bruker Topspin and MestReNova software. Diffusion was measured at 25 °C using standard Bruker pulse sequence, stegp1s, employing a stimulated echo sequence and 1 spoil gradient with a diffusion gradient, δ, set to 2 ms and the diffusion time, Δ, to 120 ms. The rectangular gradient pulses applied ranged from 2%-98% of the maximum gradient output of 48.15 Gauss/cm. The number of gradient steps was set to be 32. Individual rows of the quasi-2-D diffusion databases were phased, baseline corrected and aligned. At least three peaks were analyzed for each compound to obtain the average.

Circular dichroism spectroscopy (CD). The CD and UV spectra were recorded with a JASCO J-815 spectrometer (Tokyo, Japan) at 20 °C in a 1.0 cm cell with scaning rate of 50 nm/min. The CD spectra were measured in millidegrees and normalized into $\Delta\varepsilon_{max}$ [L mol^{-1} cm^{-1}]/λ[nm] units.

Abbreviations: DCM – dichloromethane, DMSO – dimethyl sulfoxide, EA – ethyl acetate, MeOH – methanol, 2-MTHF – 2-methyltetrahydrofuran, PE – petrol ether (b.p. 40- 65°), Py – pyridine, TFA – trifluoroacetic acid, THF – tetrahydrofuran.

SYNTHESIS

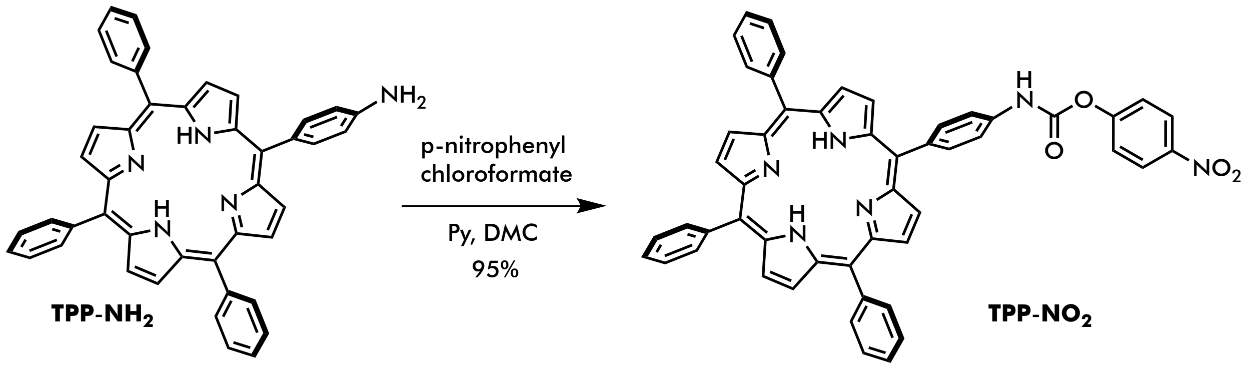

Compound **TPP-NO₂**. A mixture of **TPP-NH₂**[S1] (400 mg, 0.635 mmol, 1.0 equiv), 4-nitrophenylchloroformate (192 mg, 0.953 mmol, 1.50 equiv) and pyridine (0.1 mL, 1.27 mmol, 2.0 eq) in DCM (10 mL) was stirred at room temperature for 1.5 hours. The reaction mixture was diluted with water and extracted with DCM. The organic phase was washed with water and brine, dried over anhydrous Na_2SO_4, and evaporated to dryness. The crude was purified by column chromatography on silica gel (PE : $CHCl_3$ 1:1 , R_f = 0.25) to afford 477 mg (95 %) of **TPP-NO₂** as a purple glass.
Spectral data were in accordance with a literature.[S2]

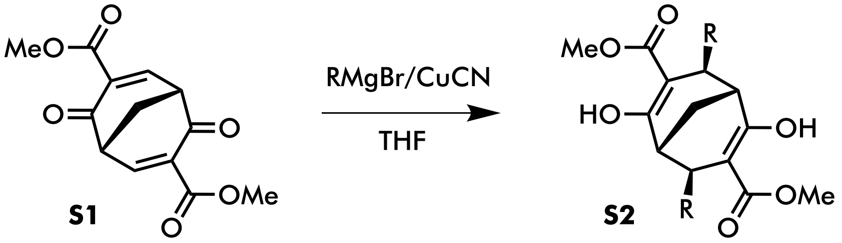

General procedure A for 1,4-addition of R-MgBr to enone S1. Under inert atmosphere Mg turnings (1.05 equiv) in anhydrous THF were activated by addition of a I_2 crystal and refluxing for 30 min until brown color of iodine faded away. A solution of R-Br (1.0 equiv) in THF was added dropwise over 15 min at room temperature. Then, the reaction mixture was refluxed for 30 min until Mg dissolved. After cooling down to room temperature, the mixture was diluted with anhydrous THF to afford 0.5 M R-MgBr solution.
To a suspension of CuCN (4.2 equiv) in anhydrous THF 0.5 M R-MgBr solution (4.0 equiv) was added dropwise over 10 min at -30 °C. The mixture was stirred for 10 min at -30 °C and 15 min at room temperature resulting in deep brown solution. The mixture was cooled to −78 °C and a solution of bicyclo[3.3.1]nona-3,7-diene-2,6-dione (+) **S1**[S2] (1.0 equiv) in anhydrous THF was added dropwise. After 10 min, the cooling bath was removed, and the reaction mixture was quenched with 1.0 M HCl solution. The mixture was diluted with H_2O and extracted with EA. Combined organic phase was diluted with H_2O (100 mL) and white precipitate was removed by filtration through CELITE. Organic phase was washed with sat. $NaHCO_3$ solution and brine, dried over anhydrous Na_2SO_4 and evaporated to dryness. The crude was purified by column chromatography on silica gel (PE : EA) to afford **S2a-c**.

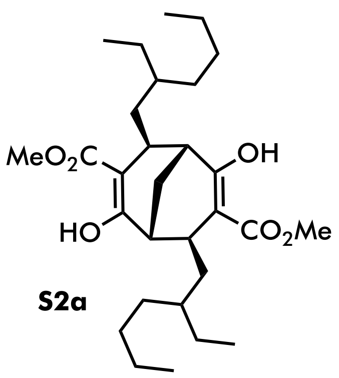

S2a

Compound S2a. Following general procedure **A** using CuCN (427 mg, 4.77 mmol), 0.5 M 2-ethylhexylmagnesium bromide solution (9.1 mL, 4.54 mmol) and **S1** (300 mg, 1.13 mmol). Purification by column chromatography on silica gel (PE : EA 100:1 , R_f = 0.11) afforded 424 mg (76 %) of **S2a** as a white solid.

^{1}H NMR (400 MHz, $CDCl_3$): δ 12.28 (s, 1H), 12.25 (s, 1H), 3.73 (s, 6H), 2.71 (dt, *J* = 10.9, 2.1 Hz, 2H), 2.45 (d, *J* = 3.6 Hz, 2H), 1.82 (t, *J* = 3.1 Hz, 2H), 1.54 – 1.08 (m, 22H), 0.95 – 0.81 (m, 12H).

^{13}C NMR (101 MHz, $CDCl_3$): δ 174.48, 174.46, 173.36, 101.03, 100.99, 51.41, 51.36, 37.54, 37.52, 37.35, 37.21, 36.62, 36.05, 35.93, 35.49, 35.36, 33.62, 31.63, 29.45, 27.84, 27.07, 24.29, 24.27, 23.39, 23.21, 17.95, 17.93, 14.36, 14.30, 11.52, 9.56.

HRMS-ESI$^+$: m/z $[M-H]^-$ calcd. for 491.3378; found 491.3386.

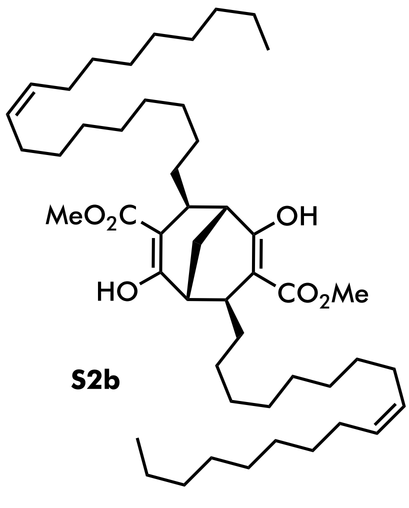

S2b

Compound S2b. Following general procedure **A** using CuCN (409 mg, 4.56 mmol), 0.5 M oleylmagnesium bromide solution (8.7 mL, 4.35 mmol) and **S1** (287 mg, 1.09 mmol). Purification by column chromatography on silica gel (PE : EA 60:1 , R_f = 0.18) afforded 779 mg (93 %) of **S2b** as a white solid.

^{1}H NMR (400 MHz, $CDCl_3$): δ 12.28 (s, 2H), 5.35 (t, *J* = 4.9 Hz, 4H), 3.74 (s. 6H), 2.61 (dt, *J* = 10.0, 2.1 Hz, 2H), 2.46 (q, *J* = 2.6 Hz, 2H), 2.02 (q, *J* = 7.1, 6.6 Hz, 8H), 1.79 (t, *J* = 3.1 Hz, 2H), 1.68 – 1.58 (m, 2H), 1.50 – 1.39 (m, 2H), 1.38 – 1.20 (m, 48H), 0.88 (t, *J* = 6.7 Hz, 6H).

^{13}C NMR (101 MHz, $CDCl_3$): δ 174.28, 173.42, 130.10, 130.00, 100.97, 51.54, 37.59, 36.11, 33.79, 32.76, 32.06, 29.95, 29.93, 29.86, 29.82, 29.67, 29.50, 29.47, 29.34, 28.07, 27.38, 22.84, 18.27, 14.25.

HRMS-ESI$^+$: m/z $[M+H]^+$ calcd. for 767.6184 ; found 767.6211.

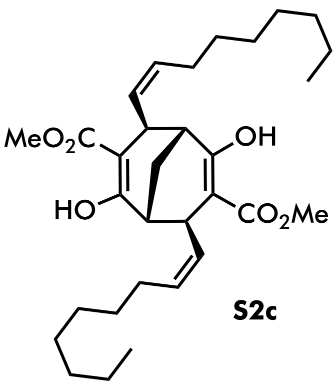

S2c

Compound S2c. Following general procedure **A** using CuCN (527 mg, 5.88 mmol), 0.5 M (Z)-1-nonenmagnesium bromide solution (11.2 mL, 5.60 mmol) and **S1** (370 mg, 1.40 mmol). Purification by column chromatography on silica gel (PE : EA 100:1 , R_f = 0.37) afforded 721 mg (99 %) of **S2c** as a white solid.

^{1}H NMR (400 MHz, $CDCl_3$): δ 12.28 (s, 2H), 5.46 – 5.29 (m, 4H), 3.69 (s, 6H), 3.65 (dd, *J* = 8.7, 1.8 Hz, 2H), 2.42 (q, *J* = 2.5 Hz, 2H), 2.27 – 2.16 (m, 4H), 1.88 (t, *J* = 3.1 Hz, 2H), 1.47 – 1.21 (m, 20H), 0.95 – 0.85 (m, 6H).

^{13}C NMR (101 MHz, $CDCl_3$): δ 173.51, 173.37, 131.86, 131.42, 99.69, 51.56, 40.54, 35.54, 32.03, 29.70, 29.51, 29.43, 27.44, 22.84, 19.39, 14.26, 14.23.

HRMS-ESI$^-$: m/z $[M-H]^-$ calcd. for 515.3367; found 515.3394.

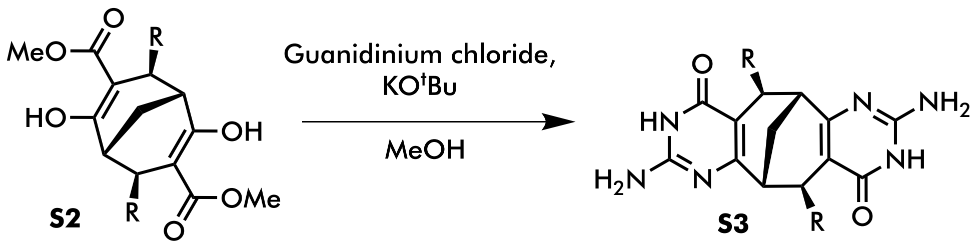

General procedure B for the synthesis of isocytosines. A mixture of the corresponding ketoester **S2** (1.0 equiv), guanidinium chloride (5.0 equiv) and KO^{t}Bu (5.0 equiv) in MeOH (5.0 mL) was heated at 100 °C for the indicated time. Then reaction mixture was diluted with 1.0 M HCl solution and the solid obtained was filtered, washed with water and MeOH to afford **S3**. Alternatively, the reaction mixture was diluted with 1.0 M HCl and extracted with $CHCl_3$. The solvent was evaporated and the residue was triturated with MeOH. The spectral characterization of **S3** was not possible due to its poor solubility and it was used directly without further purification.

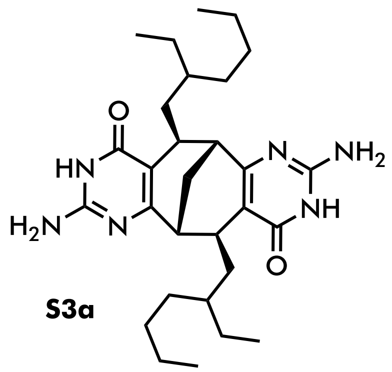

S3a

Compound S3a. Following general procedure **B** using **S2a** (390 mg, 0.79 mmol), guanidinium chloride (378 mg, 3.96 mmol) and KO^{t}Bu (444 mg, 3.96 mmol) for 36 hours. Yield: 319 mg (79 %).

HRMS-ESI$^+$: m/z $[M+H]^+$ calcd. for 511.3755; found 511.3753.

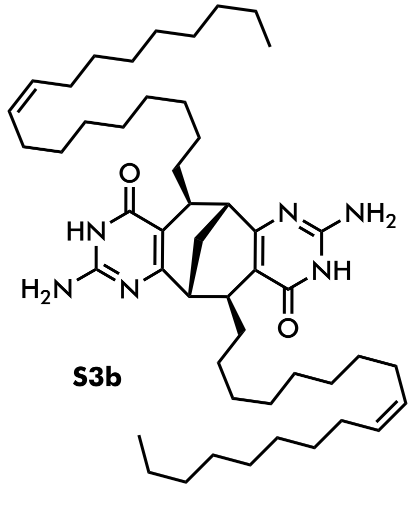

S3b

Compound S3b. Following general procedure **B** using **S2b** (760 mg, 0.99 mmol), guanidinium chloride (472 mg, 4.94 mmol) and KO^{t}Bu (554 mg, 4.94 mmol) for 24 hours. Yield: 686 mg (88 %).

HRMS-ESI$^+$: m/z [M+H]$^+$ calcd. for 787.6572; found 787.6570.

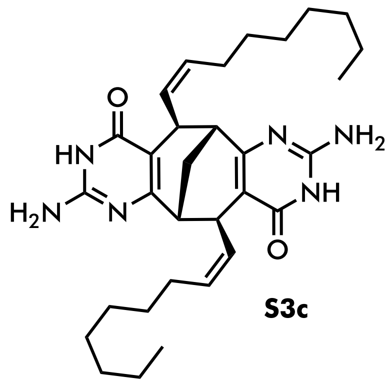

S3c

Compound S3c. Following general procedure **B** using **S2c** (715 mg, 1.39 mmol), guanidinium chloride (662 mg, 6.93 mmol) and KO^{t}Bu (777 mg, 6.93 mmol) for 24 hours. Yield: 401 mg (54 %).

HRMS-ESI$^+$: m/z [M+H]$^+$ calcd. for 535.3755; found 535.3765.

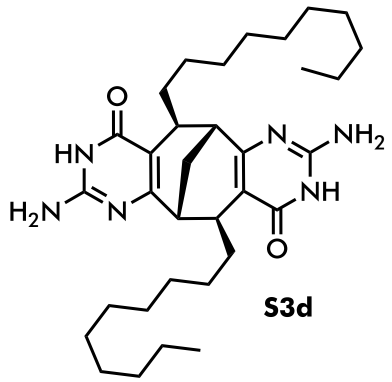

S3d

Compound **3d** was prepared following the reported procedure.[S3]

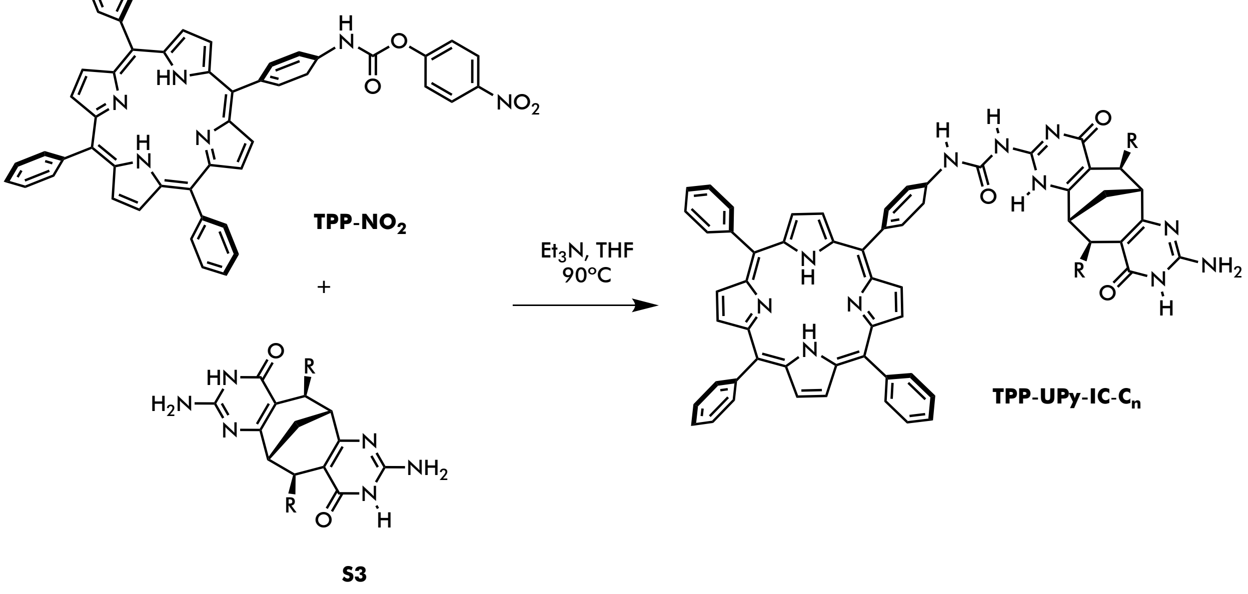

General procedure C for the synthesis of TPP-monoureas. Under inert atmosphere a mixture of corresponding **S3** (1.0 equiv) and freshly distilled anhydr. NEt_3 (10.0 equiv) in anhydr. THF was heated at 90 °C for 10 minutes until **S3** dissolved. Then, the reaction mixture was cooled down to room temperature and **TPP-NO$_2$** (1.10 equiv) was added. The reaction was heated at 90 °C for 24 hours. After cooling down to room temperature, the solvent was removed under reduced pressure. The crude was dissolved in toluene and filtered through CELITE and evaporated. The crude was subjected to size-exclusion column using toluene as an eluent. Subsequently, it was additionally purified by column chromatography on silica gel ($CHCl_3$: MeOH) to afford product **TPP-UPy-IC-C$_n$** as a purple glass.

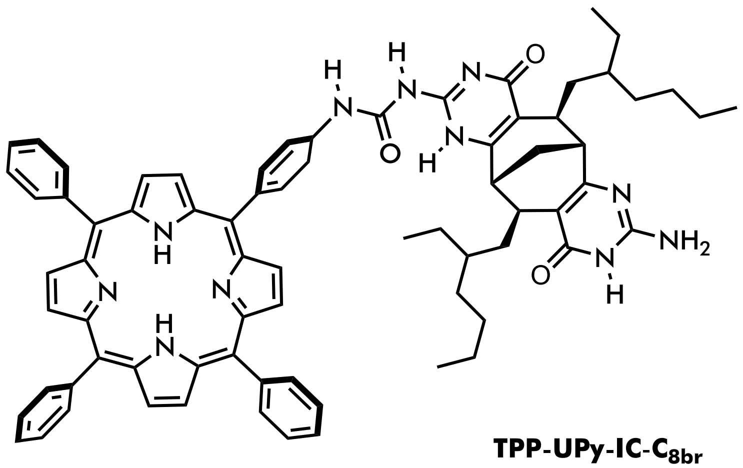

TPP-UPy-IC-C$_{8br}$

Compound TPP-UPy-IC-C$_{8br}$. Following general procedure **C** using **S3a** (100 mg, 0.20 mmol), NEt_3 (0.27 mL, 1.96 mmol) and **TPP-NO$_2$** (171.2 mg, 0.22 mmol). Purification by column chromatography on silica gel ($CHCl_3$: MeOH 100:1→50:1, R_f = 0.15) afforded 102 mg (44 %) of **TPP-UPy-IC-C$_{8br}$**.

^{1}H NMR (400 MHz, $CDCl_3$): δ 13.79 (s, 1N-H), 13.32 (s, 1N-H), 13.03 (s, 1N-H), 12.52 (s, 1N-H), 8.97 (s, 2H), 8.87 – 8.63 (m, 6H), 8.33 – 8.16 (m, 6H), 8.12 – 7.93 (m, 4H), 7.88 – 7.71 (m, 4H), 7.59 – 7.31 (m, 5H), 4.71 (d, J = 15.9 Hz, 2N-H), 3.12 (s, 1H), 3.03 (d, J = 10.1 Hz, 1H), 2.89 (d, J = 20.6 Hz, 2H), 2.24 – 2.05 (m, 3H), 1.97 – 1.78 (m, 3H), 1.54 – 1.37 (m, 6H), 1.37 – 1.16 (m, 10H), 1.12 (s, 2H), 1.05 – 0.70 (m, 12H), 0.61 (s, 2H), -2.72 (s, TPP 2N-H).

^{13}C NMR (101 MHz, DMSO-d_6): δ 160.75, 151.68, 151.00, 145.68, 145.59, 139.25, 138.33, 136.61, 130.61, 129.07, 129.01, 128.60, 124.83, 123.33, 123.31, 123.14, 121.97, 120.41, 113.70, 112.95, 77.33, 77.01, 76.69, 36.81, 36.22, 35.66, 33.39, 32.08, 29.18, 29.05, 27.17, 26.87, 24.63, 23.26, 22.96, 22.91, 17.20, 14.05, 13.98, 13.91, 11.08, 10.91, 8.74.

HRMS-ESI$^+$: m/z $[M+H]^+$ calcd. for 1166.6127; found 1166.6189.

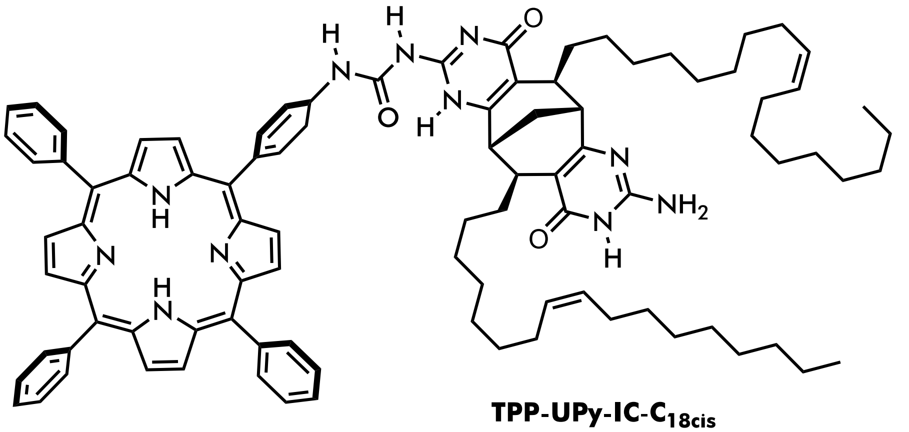

TPP-UPy-IC-C$_{18cis}$

Compound TPP-UPy-IC-C$_{18cis}$. Following general procedure **C** using **S3b** (70 mg, 0.089 mmol), NEt_3 (0.12 mL, 0.89 mmol) and **TPP-NO$_2$** (78 mg, 0.098 mmol). Purification by column chromatography on silica gel ($CHCl_3$: MeOH 100:1→70:1→50:1→20:1, R_f = 0.12) afforded 14 mg (11 %) of **TPP-UPy-IC-C$_{18cis}$**.

^{1}H NMR (400 MHz, $CDCl_3$): δ 13.65 (s, 1N-H), 13.06 (s, 1N-H), 12.95 (s, 1N-H), 12.60 (s, 1N-H), 9.06 – 8.94 (m, 2H), 8.92 – 8.73 (m, 6H), 8.37 – 8.06 (m, 10H), 7.89 – 7.72 (m, 5H), 7.64 – 7.44 (m, 4H), 5.43 – 5.26 (m, 1H), 5.22 – 4.99 (m, 2H), 4.86 (s, 2N-H), 4.82 – 4.58 (m, 1H), 3.09 – 2.77 (m, 5H), 2.18 – 1.82 (m, 14H), 1.80 – 1.58 (m, 9H), 1.53 – 1.33 (m, 14H), 1.18 – 0.98 (m, 18H), 0.91 – 0.86 (m, 6H), 0.86 – 0.67 (m, 6H), -2.70 (s, TPP 2N-H).

^{13}C NMR (101 MHz, $CDCl_3$+TFA): δ 162.04, 152.93, 151.37, 150.63, 145.66, 145.57, 139.07, 139.04, 138.28, 137.64, 136.85, 130.78, 130.03, 129.99, 129.90, 129.33, 129.30, 128.79, 128.67, 124.83, 123.54, 123.46, 121.80, 120.95, 118.88, 113.60, 43.60, 38.36, 32.96, 32.75, 32.60, 31.89, 31.72, 29.75, 29.70, 29.62, 29.54, 29.50, 29.30, 29.27, 29.24, 29.21, 29.18, 29.13, 29.04, 28.99, 28.95, 28.83, 27.57, 27.20, 22.65, 22.58, 22.55, 21.98, 21.78, 17.14, 14.01, 13.97.

HRMS-ESI$^+$: m/z $[M+H]^+$ calcd. for 1442.8944 ; found 1442.8833.

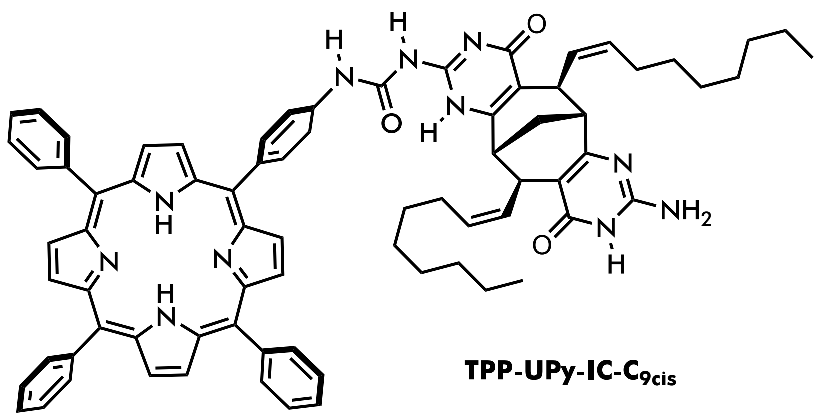

TPP-UPy-IC-C$_{9cis}$

Compound TPP-UPy-IC-C$_{9cis}$. Following general procedure C using **S3c** (100 mg, 0.19 mmol), NEt_3 (0.26 mL, 1.87 mmol) and **TPP-NO$_2$** (164 mg, 0.21 mmol). Purification by column chromatography on silica gel ($CHCl_3$:MeOH 100:1→70:1→50:1→20:1, R_f = 0.12) afforded 68 mg (31 %) of **TPP-UPy-IC-C$_{9cis}$**.

^{1}H NMR (400 MHz, $CDCl_3$): δ 13.46 (s, 1N-H), 13.44 (s, 1N-H), 12.91 (s, 1N-H), 12.60 (s, 1N-H), 9.04 – 8.92 (m, 2H), 8.92 – 8.74 (m, 6H), 8.34 – 8.12 (m, 8H), 8.14 – 8.01 (m, 2H), 7.89 – 7.69 (m, 5H), 7.59 – 7.41 (m, 4H), 5.86 – 5.35 (m, 4H), 4.91 (s, 2N-H), 4.06 (d, *J* = 8.5 Hz, 1H), 3.94 (d, *J* = 8.5 Hz, 1H), 2.90 (s, 1H), 2.82 (s, 1H), 2.69 – 2.39 (m, 4H), 2.30 – 1.93 (m, 4H), 1.30 – 1.19 (m, 6H), 1.15 – 0.96 (m, 8H), 0.95 – 0.70 (m, 4H), 0.65 – 0.47 (m, 4H), 0.39 – 0.22 (m, 2H), -2.70 (s, TPP 2N-H).

^{13}C NMR (101 MHz, $CDCl_3$+TFA): δ 161.33, 151.55, 150.94, 145.69, 145.67, 145.64, 145.62, 139.19, 139.13, 138.33, 137.94, 137.26, 136.70, 136.51, 130.67, 129.12, 128.63, 124.04, 123.41, 123.29, 121.83, 120.49, 117.16, 112.09, 77.32, 77.00, 76.69, 35.86, 35.56, 35.40, 31.80, 31.77, 30.82, 29.50, 29.43, 29.36, 29.31, 29.25, 29.23, 29.20, 29.08, 28.02, 27.95, 27.79, 22.61, 22.57, 18.02, 14.03, 13.99, 13.94, 13.91.

HRMS-ESI$^+$: m/z $[M+H]^+$ calcd. for 1190.6127; found 1190.615

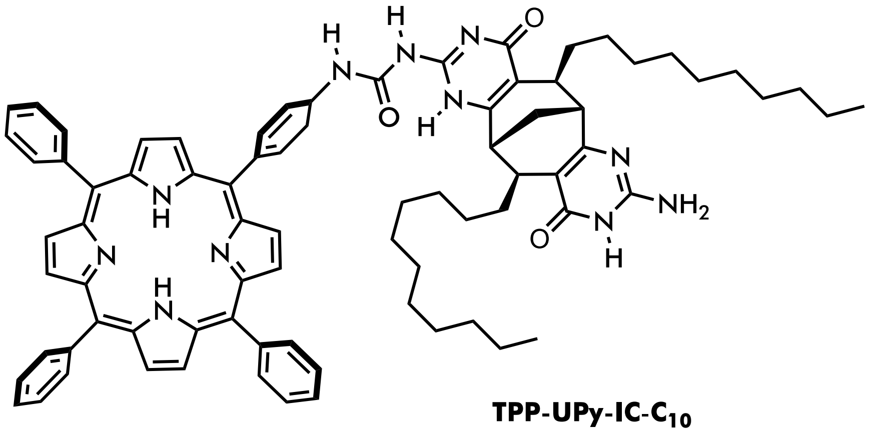

TPP-UPy-IC-C$_{10}$

Compound TPP-UPy-IC-C_{10}. Following general procedure **C** using **S3d** (55 mg, 0.097 mmol), NEt_3 (0.14 mL, 0.97 mmol) and **TPP-NO_2** (85 mg, 0.11 mmol) for 24 hours. Purification by column chromatography on silica gel ($CHCl_3$: MeOH 70:1→50:1→30:1→20:1, R_f = 0.08) afforded 102 mg (44 %) of **TPP-UPy-IC-C_{10}**.

^{1}H NMR (400 MHz, $CDCl_3$): δ 13.60 (s, 1N-H), 13.07 (s, 1N-H), 12.94 (s, 1N-H), 12.59 (s, 1N-H), 8.98 (s, 2H), 8.92 – 8.76 (m, 7H), 8.24 (br. s, 6H), 8.12 (br. s, 4H), 7.79 (s, 4H), 7.52 (s, 4H), 4.89 (s, 2N-H), 3.16 – 2.72 (m, 4H), 2.27 (br. s, 2H), 2.11 (br. s, 4H), 1.56 – 1.01 (m, 24H), 0.94 – 0.73 (m, 4H), 0.70 – 0.40 (m, 8H), 0.26 (s, 2H), -2.71 (s, TPP 2N-H).

^{13}C NMR (101 MHz, $CDCl_3$+TFA): δ 161.07, 151.89, 151.75, 151.59, 150.79, 145.64, 145.58, 143.11. 139.18, 139.12, 138.32, 138.08, 136.63, 130.68, 129.13, 128.62, 123.39, 123.20, 121.97, 120.55, 118.62, 113.69, 38.24, 39.09, 32.97, 32.68, 31.88, 29.80, 29.69, 29.62, 29.61, 29.56, 29.54, 29.28, 29.26, 29.22, 27.55, 22.64, 17.22, 14.02, 14.00.

HRMS-ESI$^+$: m/z $[M+H]^+$ calcd. for 1222.6753; found 1222.6729.

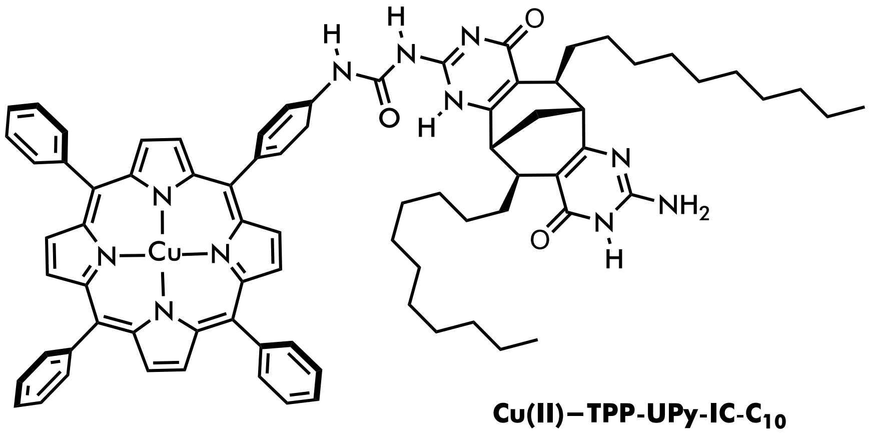

Cu(II)–TPP-UPy-IC-C_{10}

Compound Cu(II)-TPP-UPy-IC-C_{10}. Under inert atmosphere a mixture of **TPP-UPy-IC-C_{10}** (5 mg, 0.0041 mmol, 1.0 equiv) and copper (II) acetate (0.8 mg, 0.045 mmol, 1.1 equiv) in $CHCl_3$ was heated at 50 °C for 12 hours. After cooling down to room temperature, the solvent was removed under reduced pressure. The crude was purified by column chromatography on silica gel ($CHCl_3$: MeOH 50:1 → 20:1, R_f = 0.46), then dissolved in minimum amount of $CHCl_3$, precipitated with MeOH, filtered and washed with MeOH to afford 4.1 mg (78 %) of **Cu(II)-TPP-UPy-IC-C_{10}** as a red solid.

HRMS-ESI$^+$: m/z $[M+H]^+$ calcd. for 1345.6988; found 1345.6994.

Copies of NMR spectra

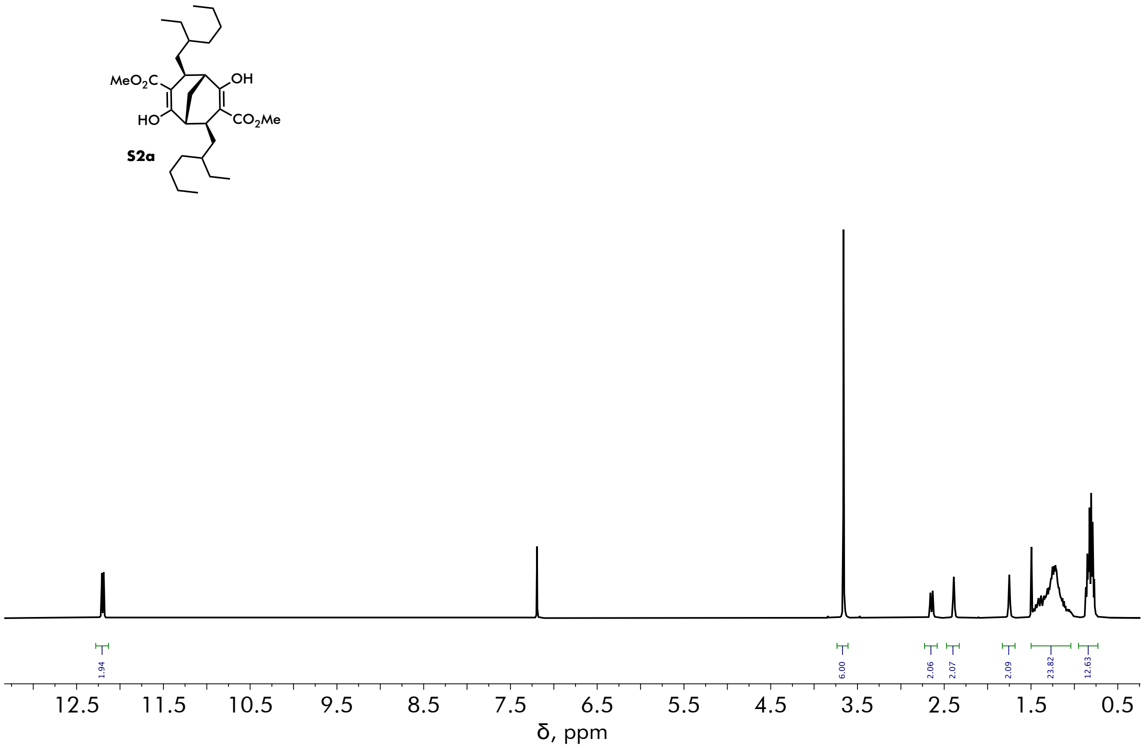

Figure S1. ^{1}H NMR ($CDCl_3$, 400 MHz) of **S2a**.

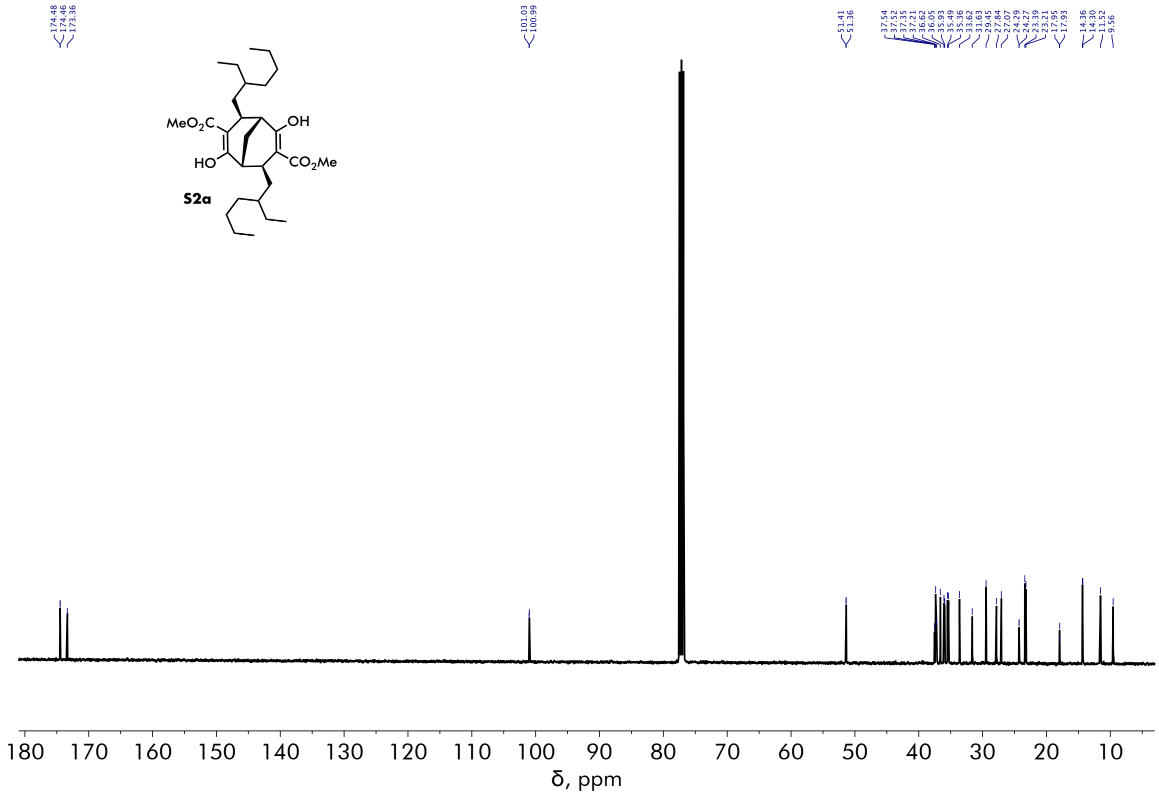

Figure S2. ^{13}C NMR ($CDCl_3$, 101 MHz) of **S2a**.

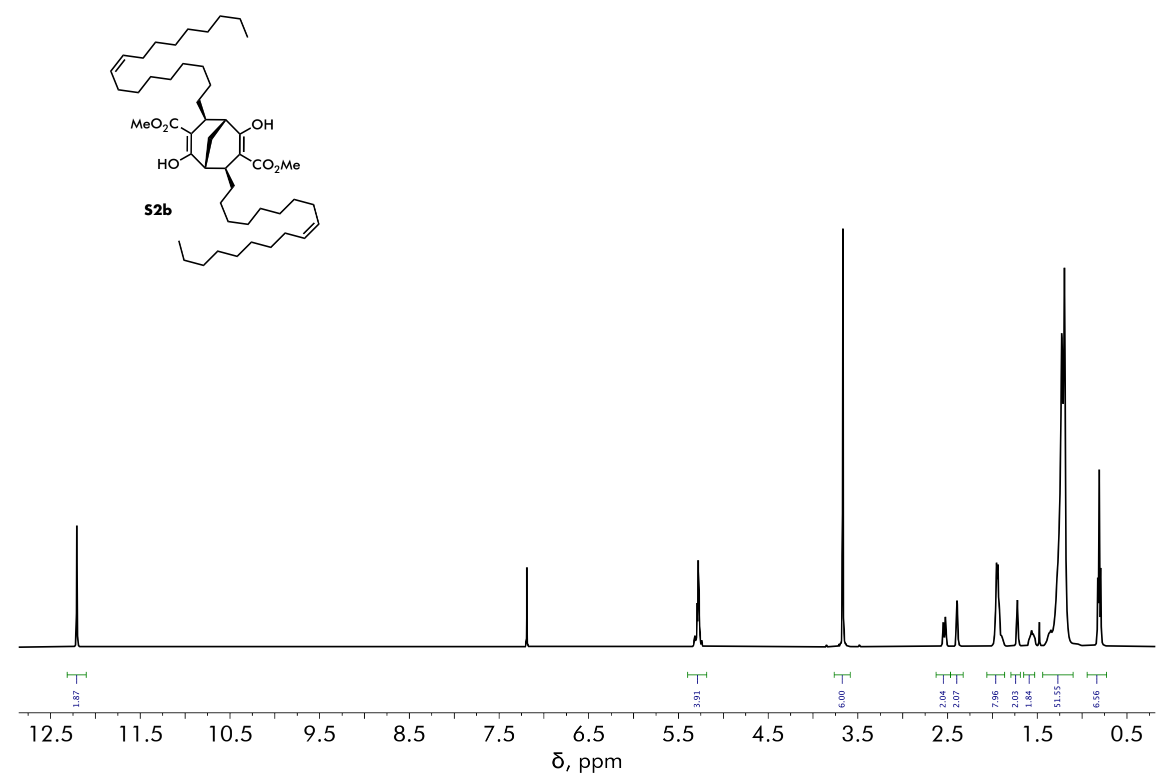

Figure S3. ^{1}H NMR ($CDCl_3$, 400 MHz) of **S2b**.

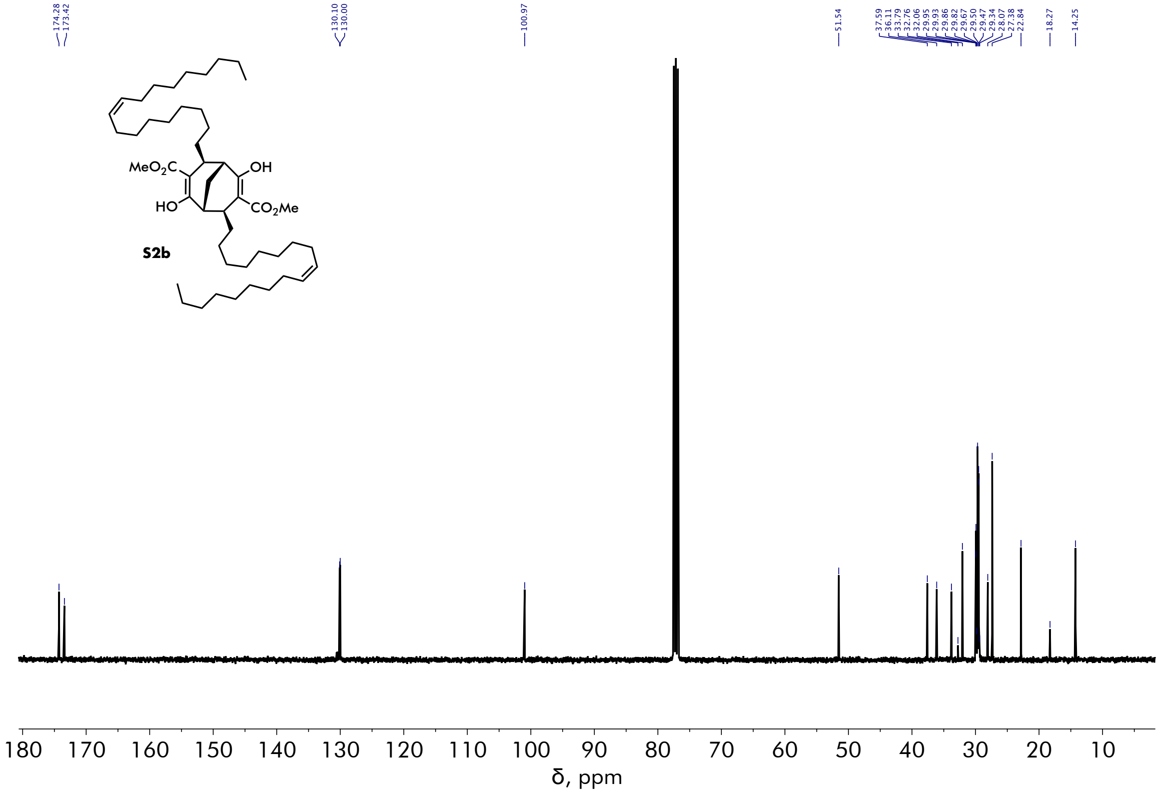

Figure S4. ^{13}C NMR ($CDCl_3$, 101 MHz) of **S2b**.

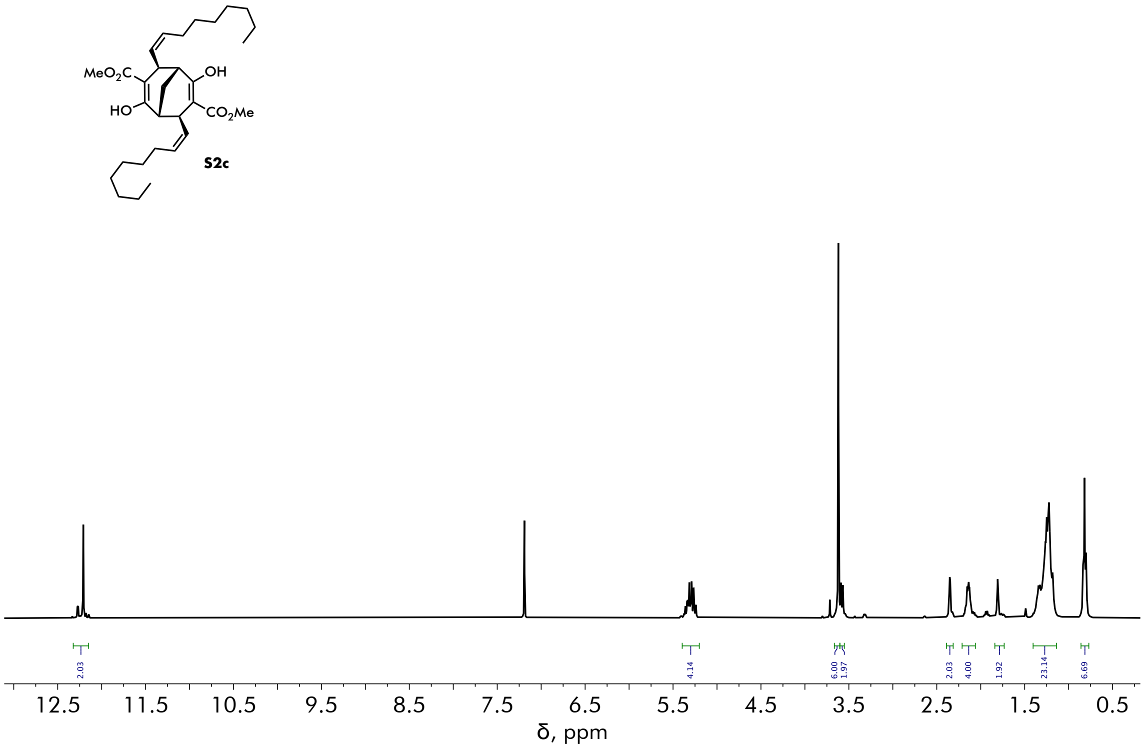

Figure S5. ^{1}H NMR ($CDCl_3$, 400 MHz) of **S2c**.

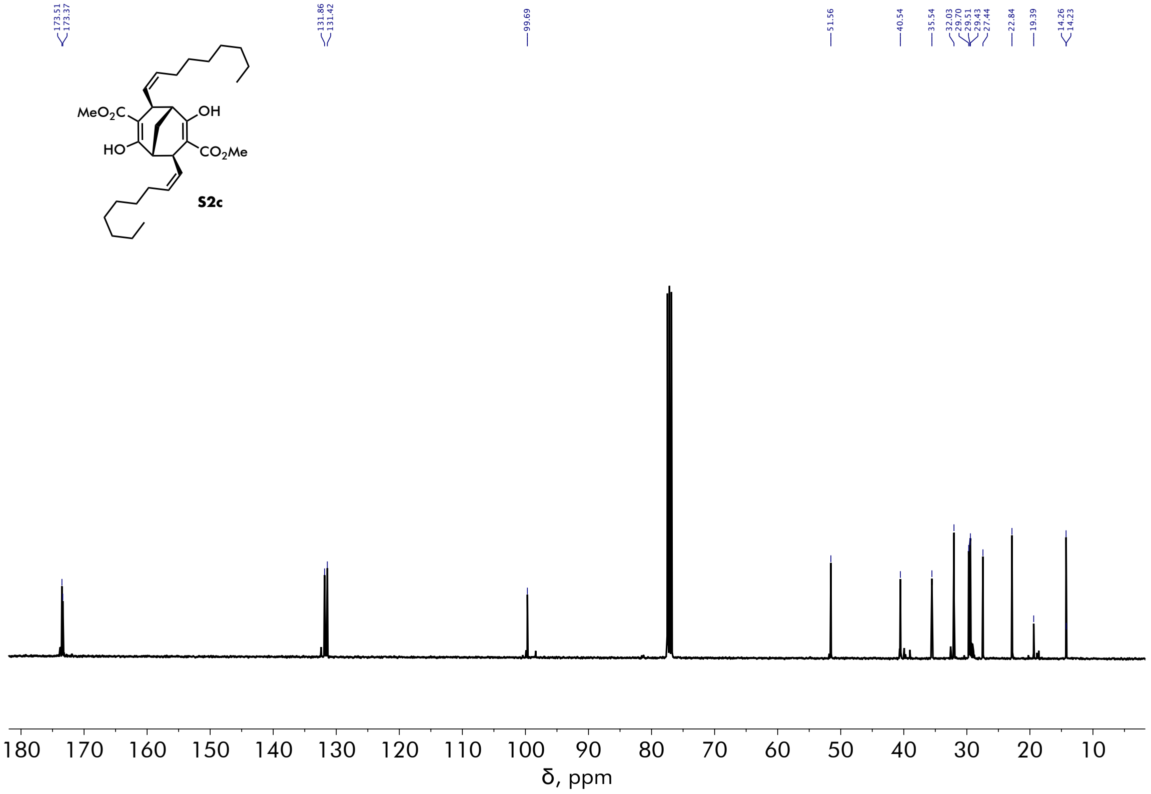

Figure S6. ^{13}C NMR ($CDCl_3$, 101 MHz) of **S2c**.

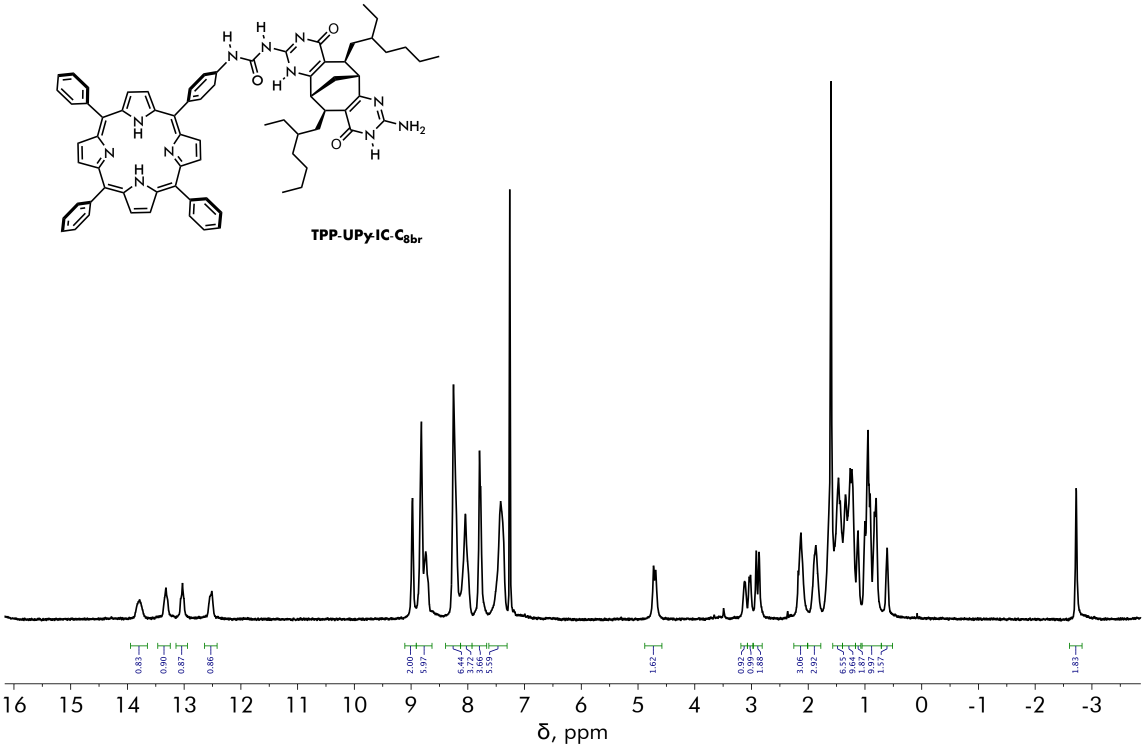

Figure S7. ^{1}H NMR ($CDCl_3$, 400 MHz) of (**TPP-UPy-IC-C$_{8br}$**)$_4$.

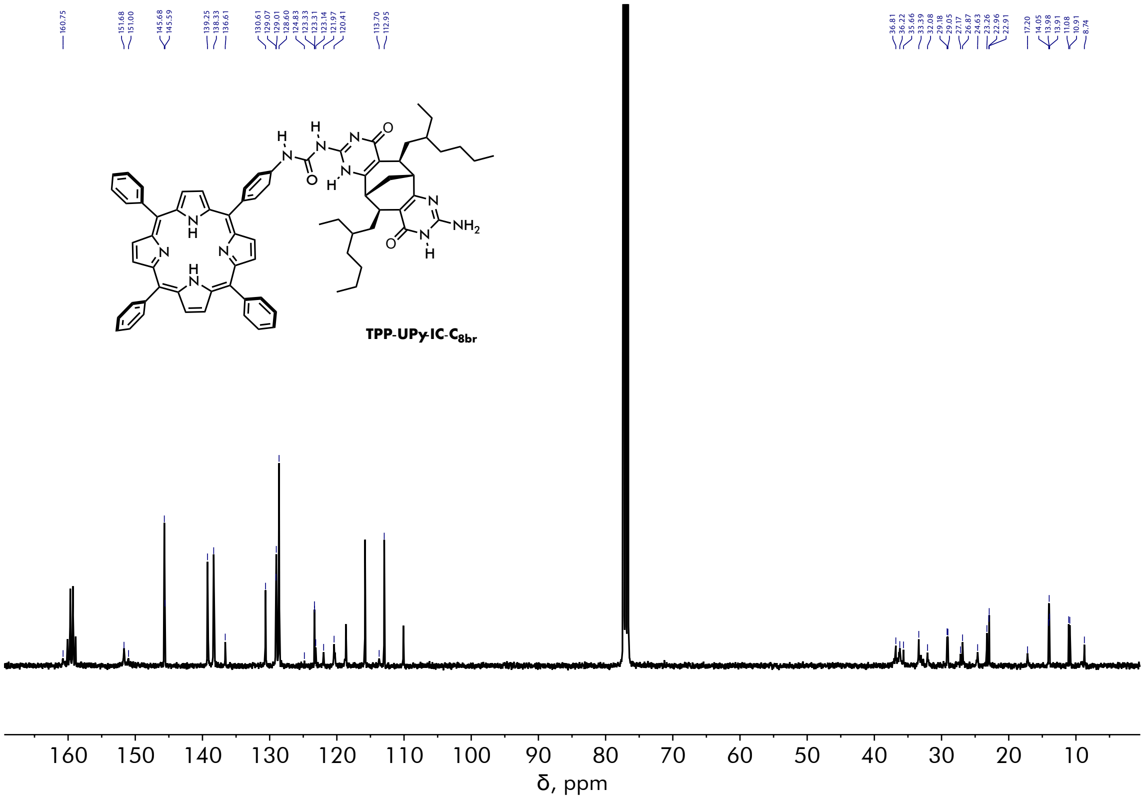

Figure S8. ^{13}C NMR ($CDCl_3$/TFA, 101 MHz) of (**TPP-UPy-IC-C$_{8br}$**)$_4$.

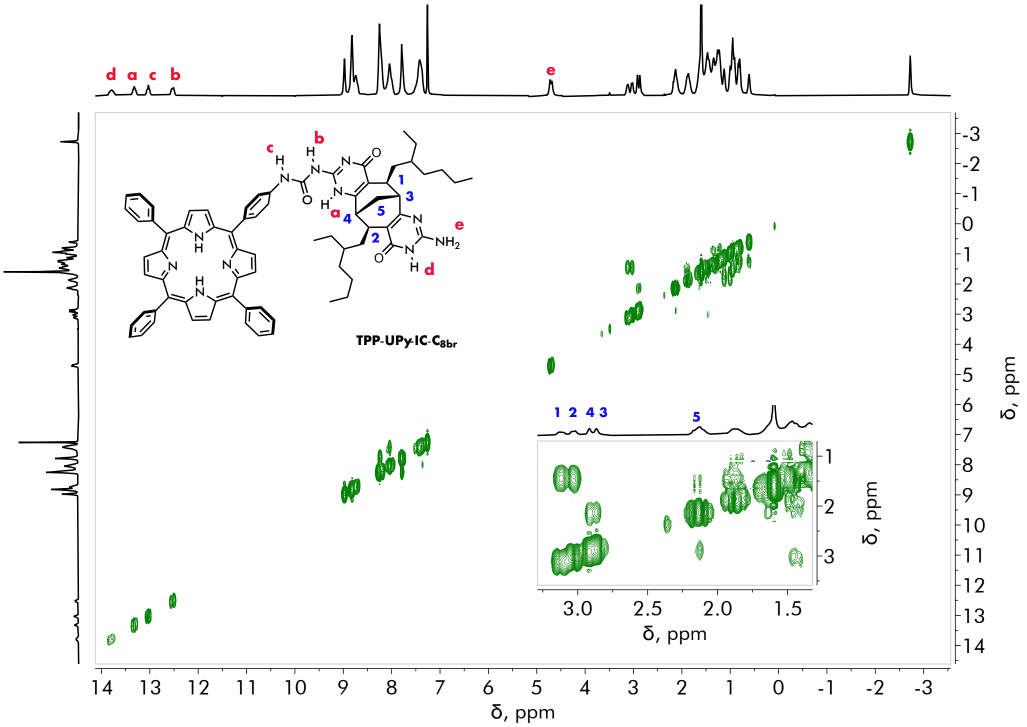

Figure S9. COSY spectrum of (**TPP-UPy-IC-C$_{8br}$**)$_4$.

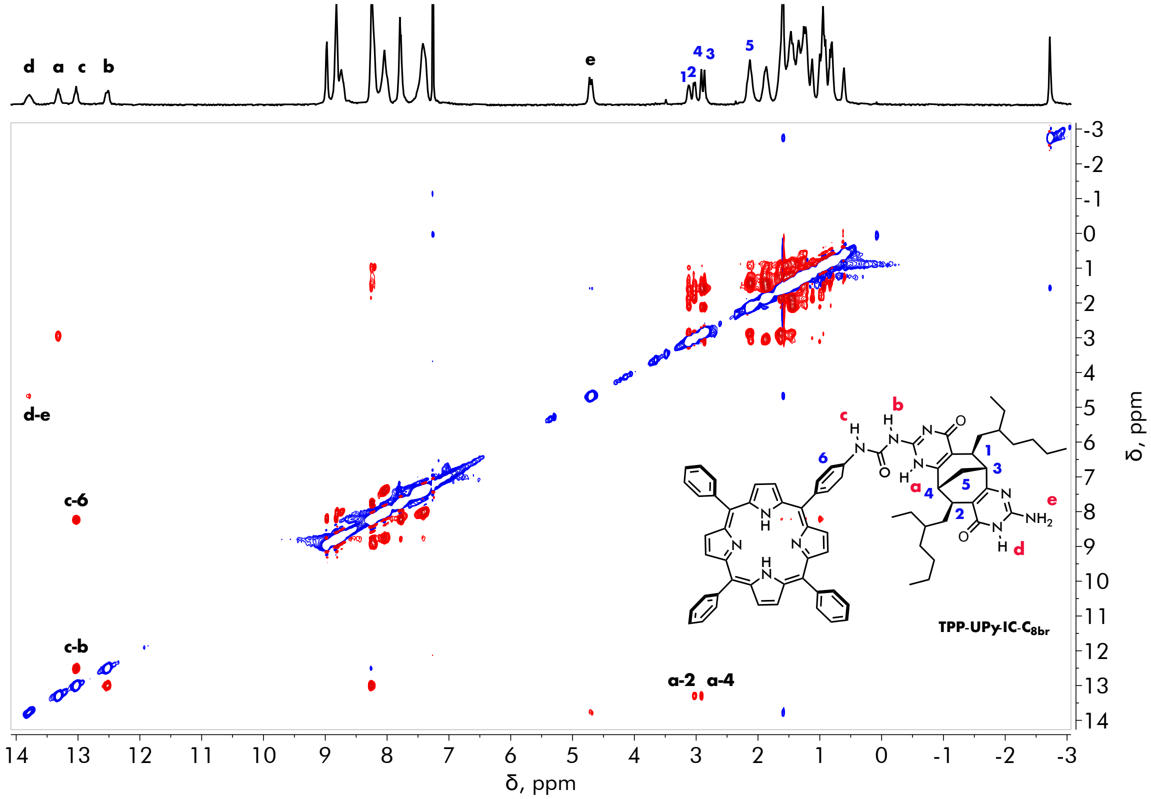

Figure S10. ROESY spectrum of (**TPP-UPy-IC-C$_{8br}$**)$_4$.

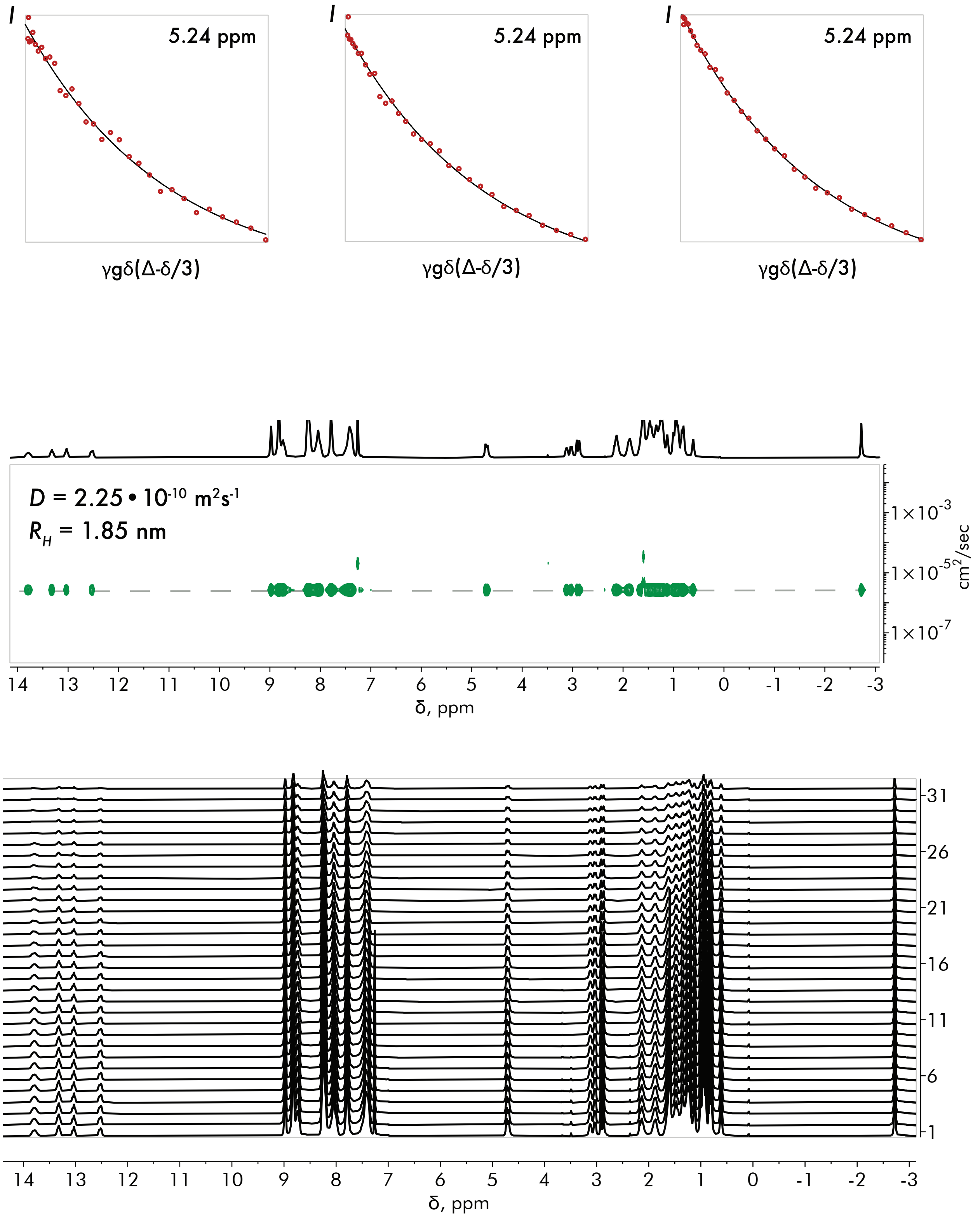

Figure S11. DOSY ($CDCl_3$, 400 MHz) spectrum of (**TPP-UPy-IC-C$_{8br}$**)$_4$ (fitting of signal decay to Stejskal-Tanner equation (top), DOSY trace (middle) and stacked spectra for each data point (bottom)).

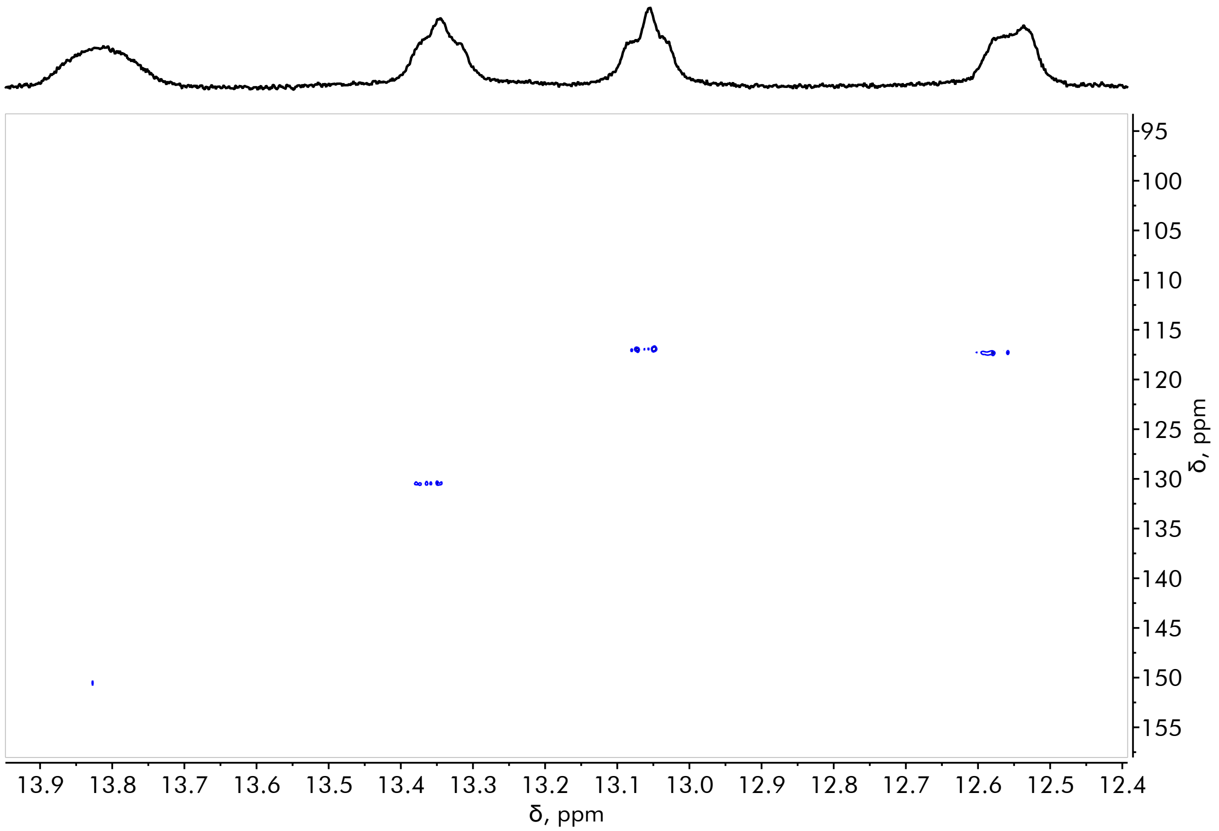

Figure S12. ^{1}H-^{15}N HSQC ($CDCl_3$, 400 MHz) spectrum of (**TPP**-**UPy**-**IC**-**C$_{8br}$**)$_4$.

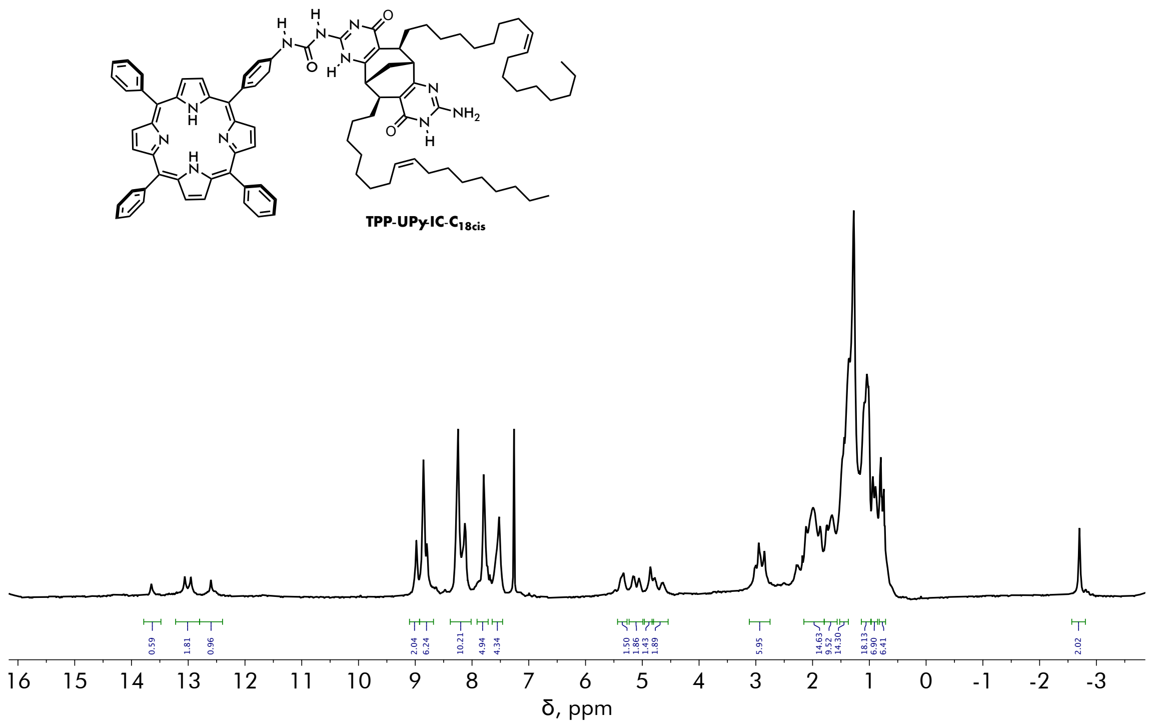

Figure S13. ^{1}H NMR ($CDCl_3$, 400 MHz) of (**TPP-UPy-IC-C$_{18cis}$**)$_4$.

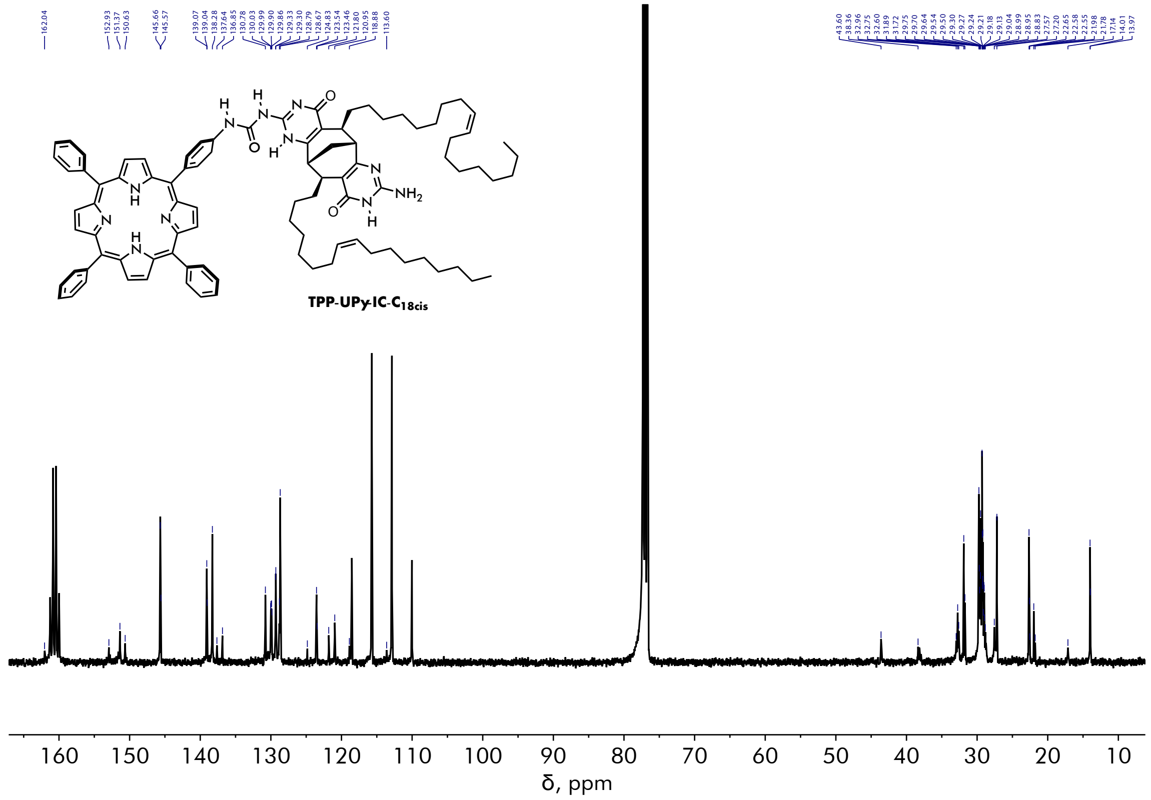

Figure S14. ^{13}C NMR ($CDCl_3$/TFA, 101 MHz) of **TPP-UPy-IC-C$_{18cis}$**.

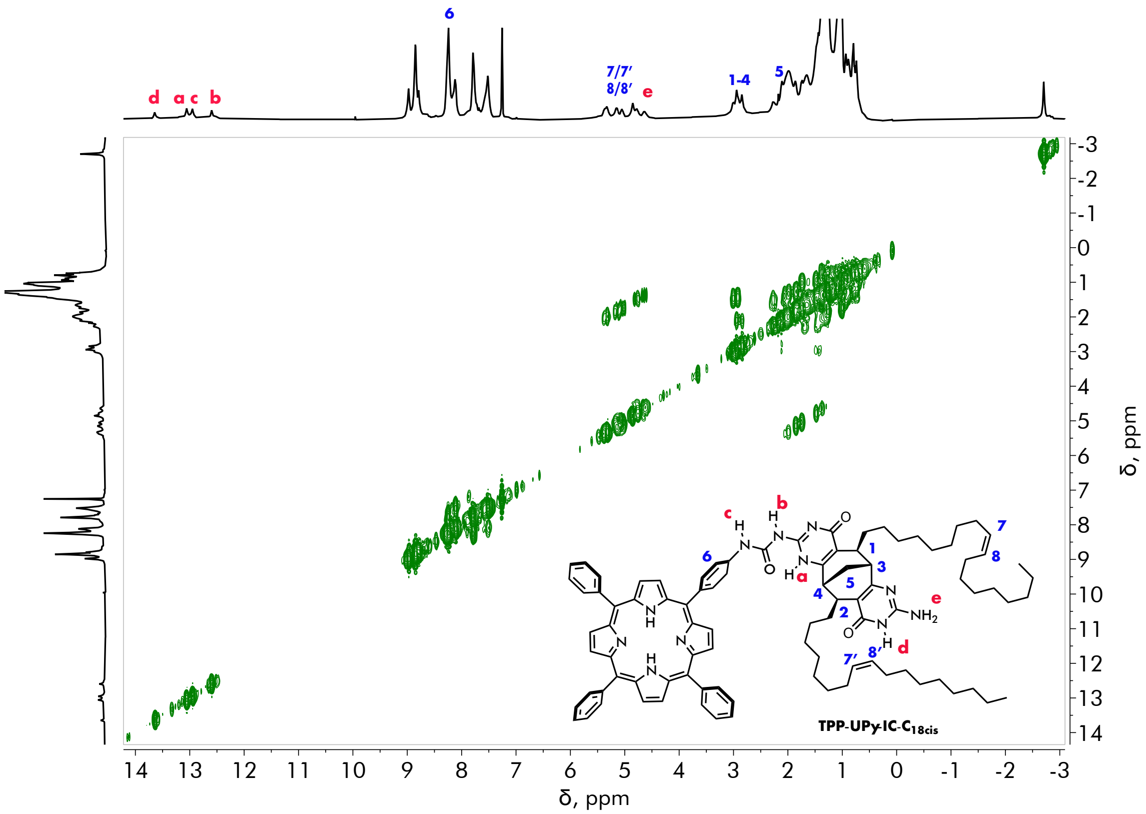

Figure S15. COSY spectrum of (**TPP-UPy-IC-C$_{18cis}$**)$_4$.

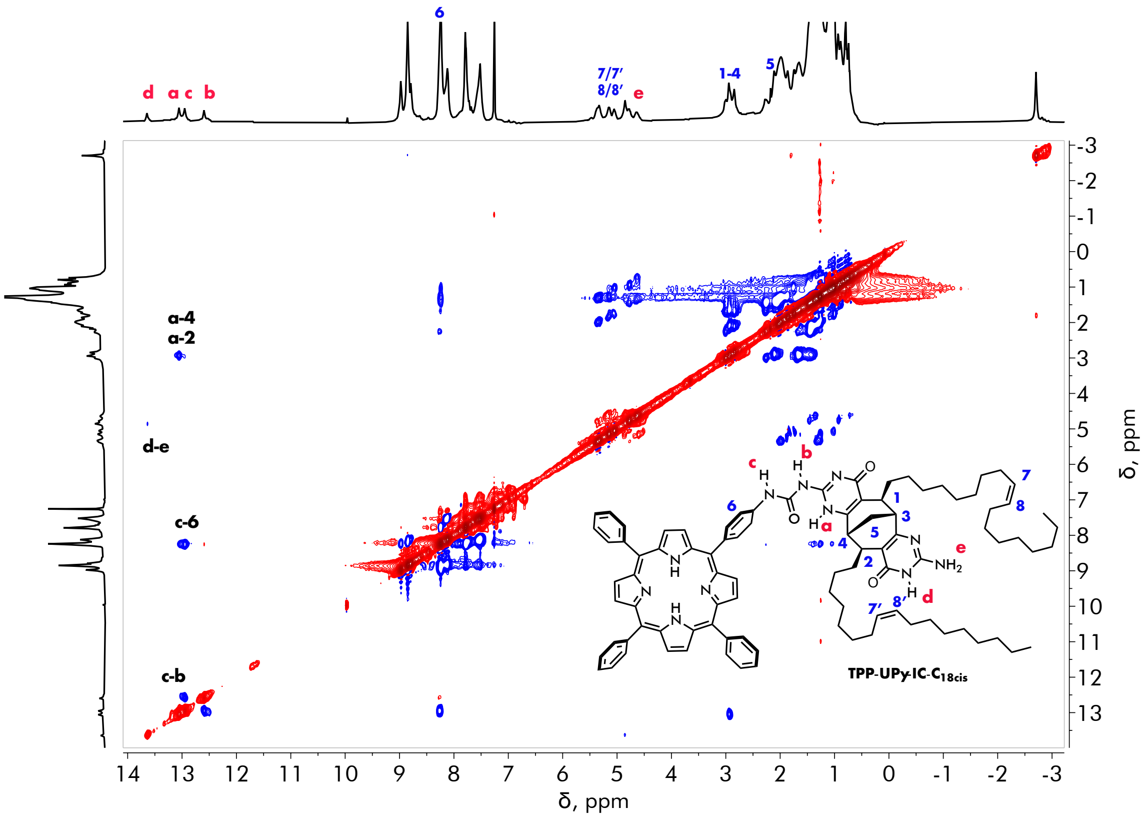

Figure S16. ROESY spectrum of (**TPP-UPy-IC-C$_{18cis}$**)$_4$.

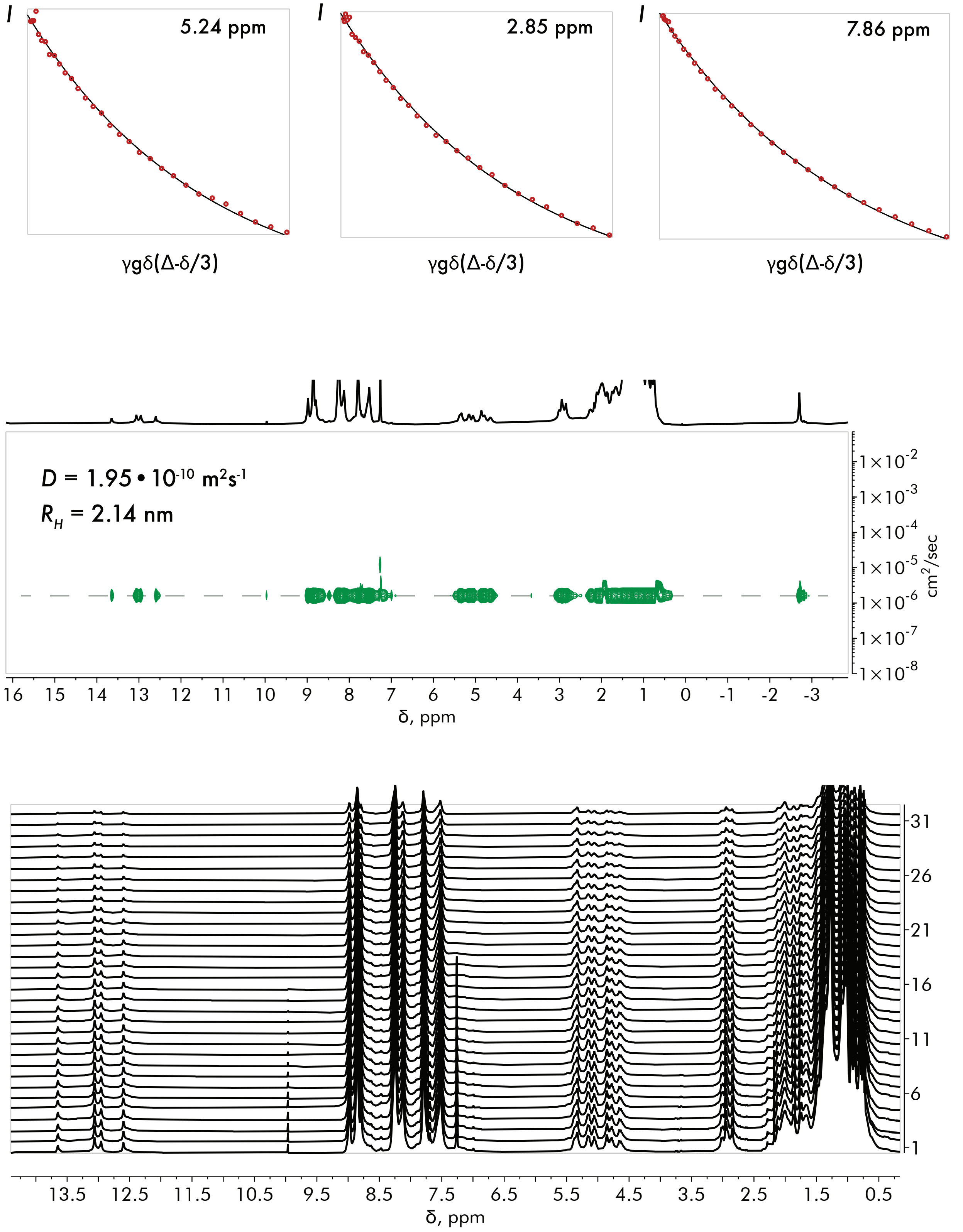

Figure S17. DOSY ($CDCl_3$, 400 MHz) spectrum of (**TPP**-**UPy**-**IC**-**C$_{18cic}$**)$_4$ (fitting of signal decay to Stejskal-Tanner equation (top), DOSY trace (middle) and stacked spectra for each data point (bottom)).

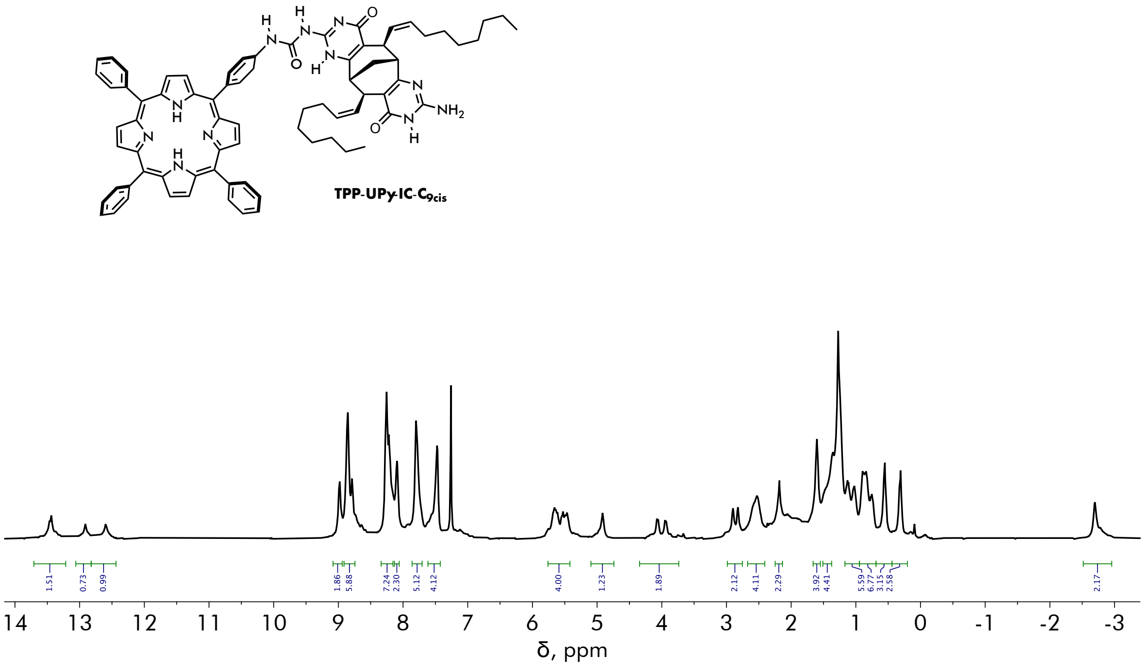

Figure S18. ^{1}H NMR ($CDCl_3$, 400 MHz) of (**TPP-UPy-IC-C$_{9cis}$**)$_4$.

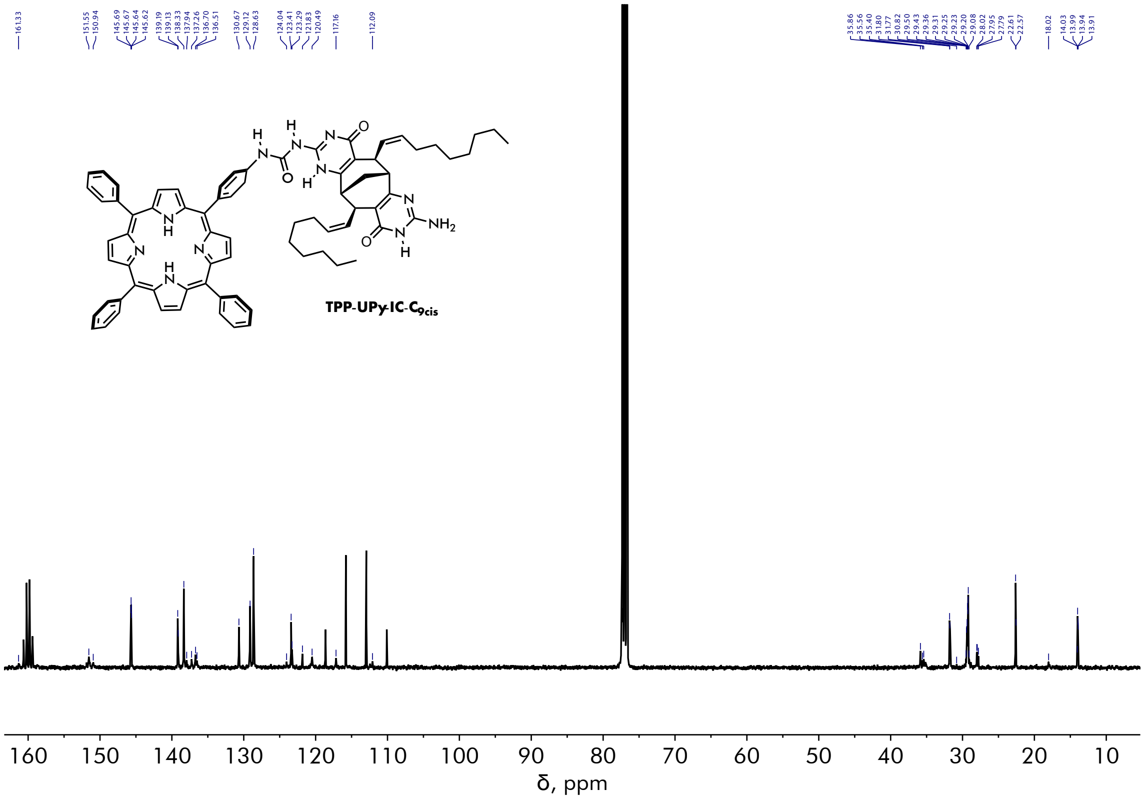

Figure S19. ^{13}C NMR ($CDCl_3$/TFA 101 MHz) of **TPP-UPy-IC-C$_{9cis}$**.

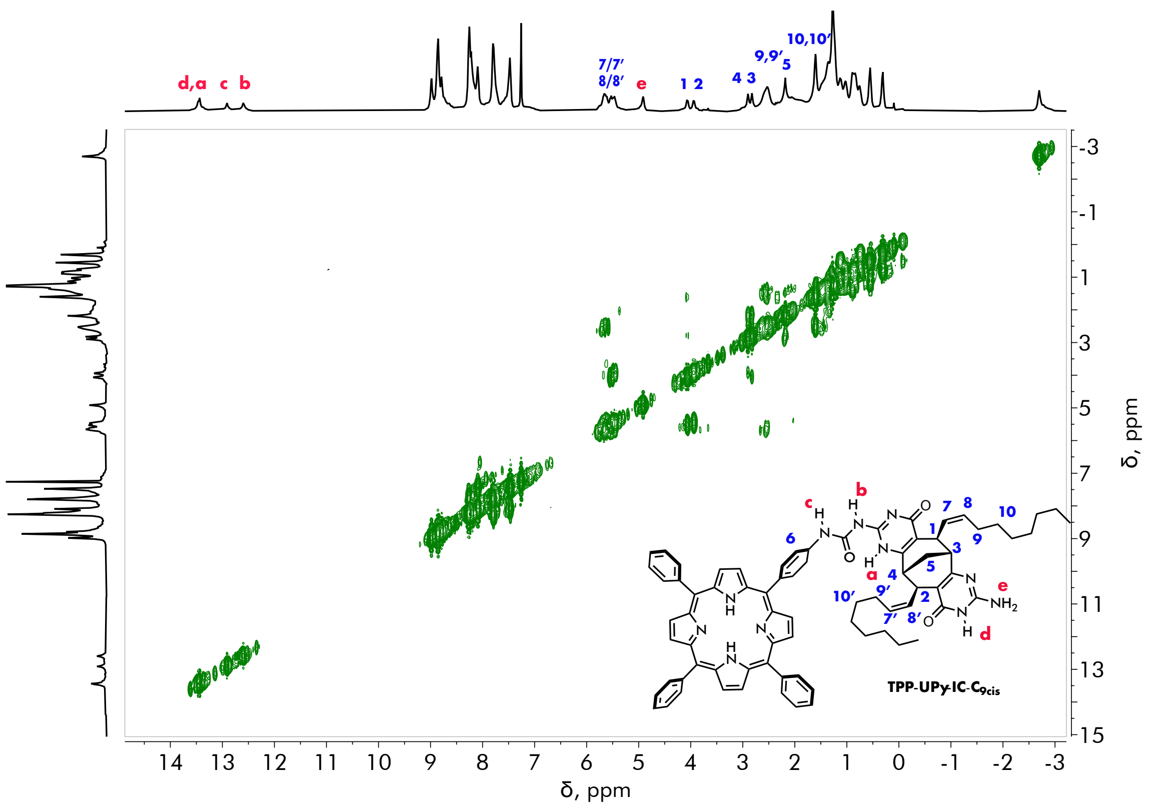

Figure S20. COSY spectrum of (**TPP-UPy-IC-C$_{9cis}$**)$_4$.

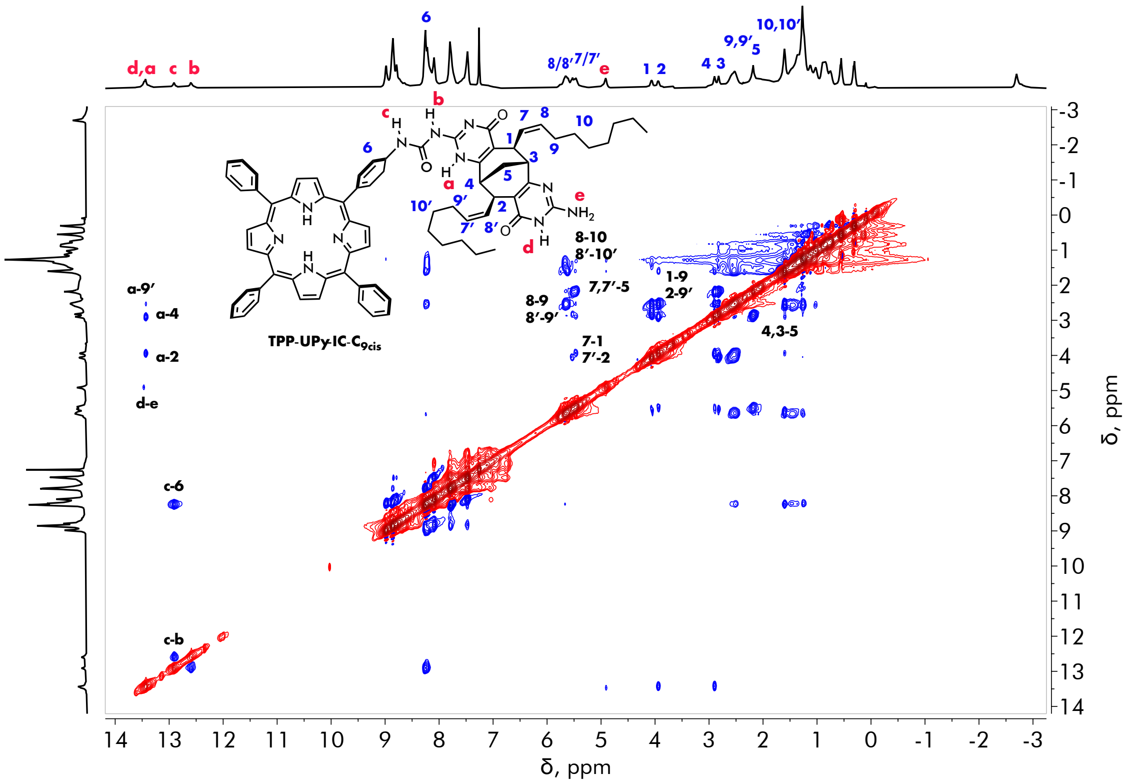

Figure S21. ROESY spectrum of (**TPP-UPy-IC-C$_{9cis}$**)$_4$.

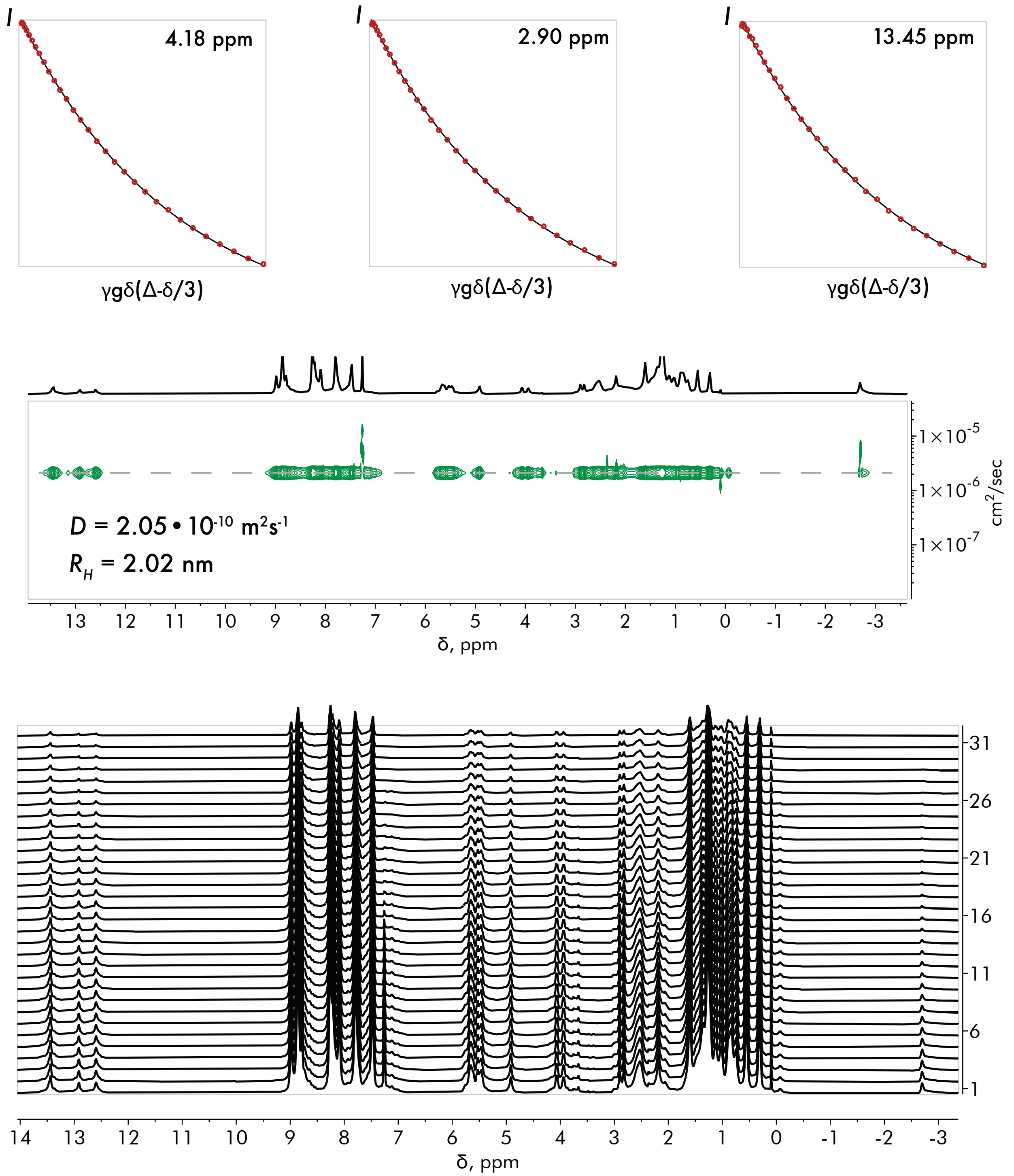

Figure S22. DOSY ($CDCl_3$, 400 MHz) spectrum of (**TPP-UPy-IC-C$_{9cis}$**)$_4$ (fitting of signal decay to Stejskal-Tanner equation (top), DOSY trace (middle) and stacked spectra for each data point (bottom)).

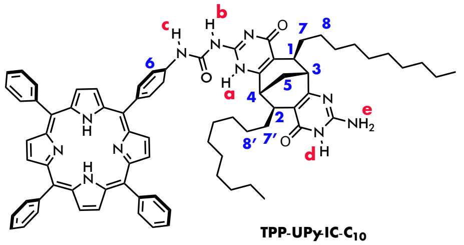

TPP-UPy-IC-C$_{10}$

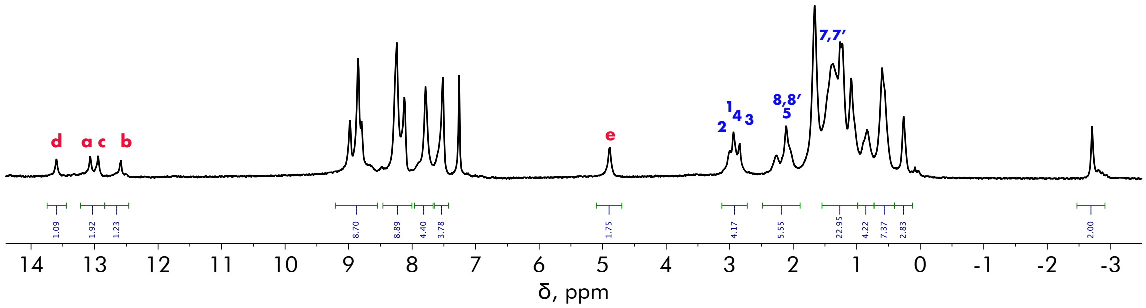

Figure S23. ^{1}H NMR ($CDCl_3$, 400 MHz) spectrum of (**TPP-UPy-IC-C$_{10}$**)$_4$.

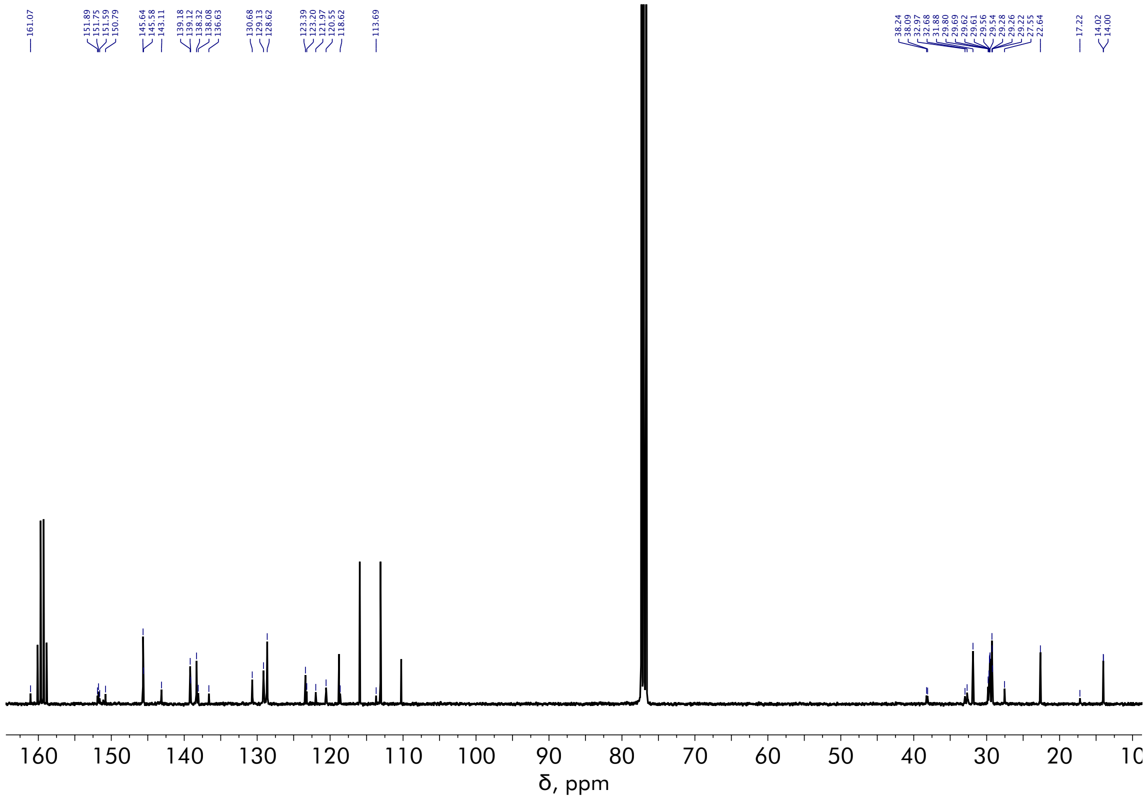

Figure S24. ^{13}C NMR ($CDCl_3$/TFA, 101 MHz) spectrum of **TPP-UPy-IC-C$_{10}$**.

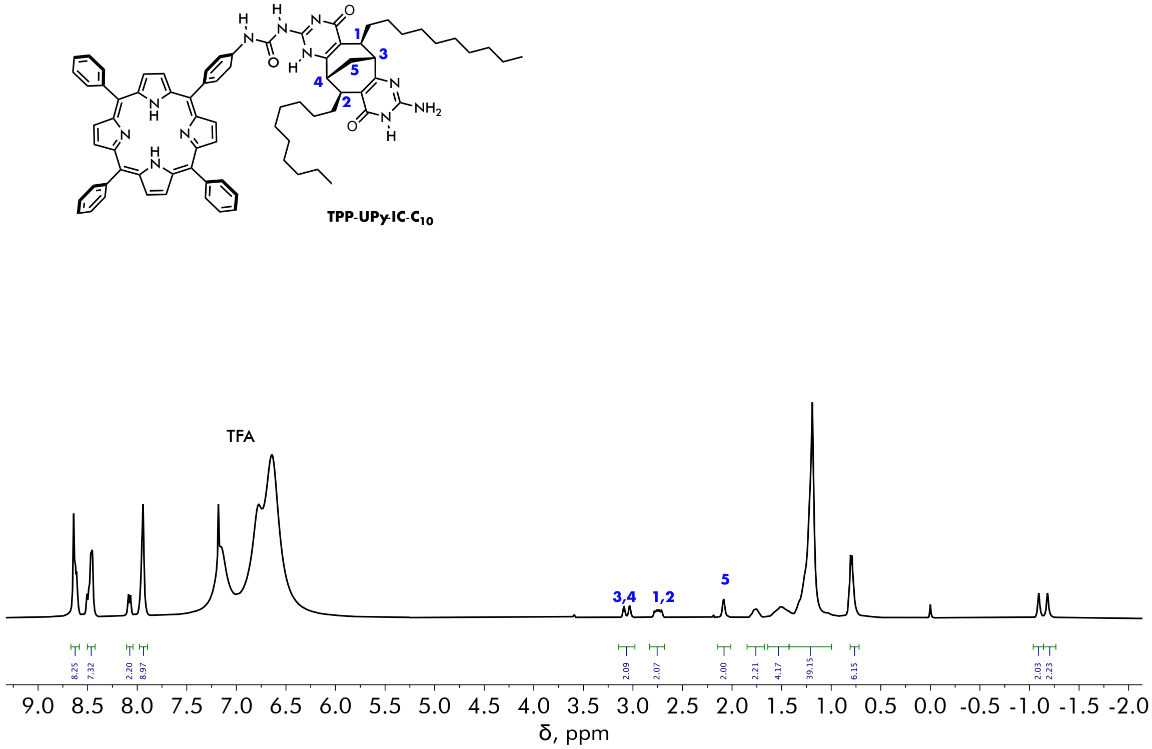

Figure S25. ^{1}H NMR ($CDCl_3$/TFA, 400 MHz) spectrum of **TPP-UPy-IC-C₁₀**.

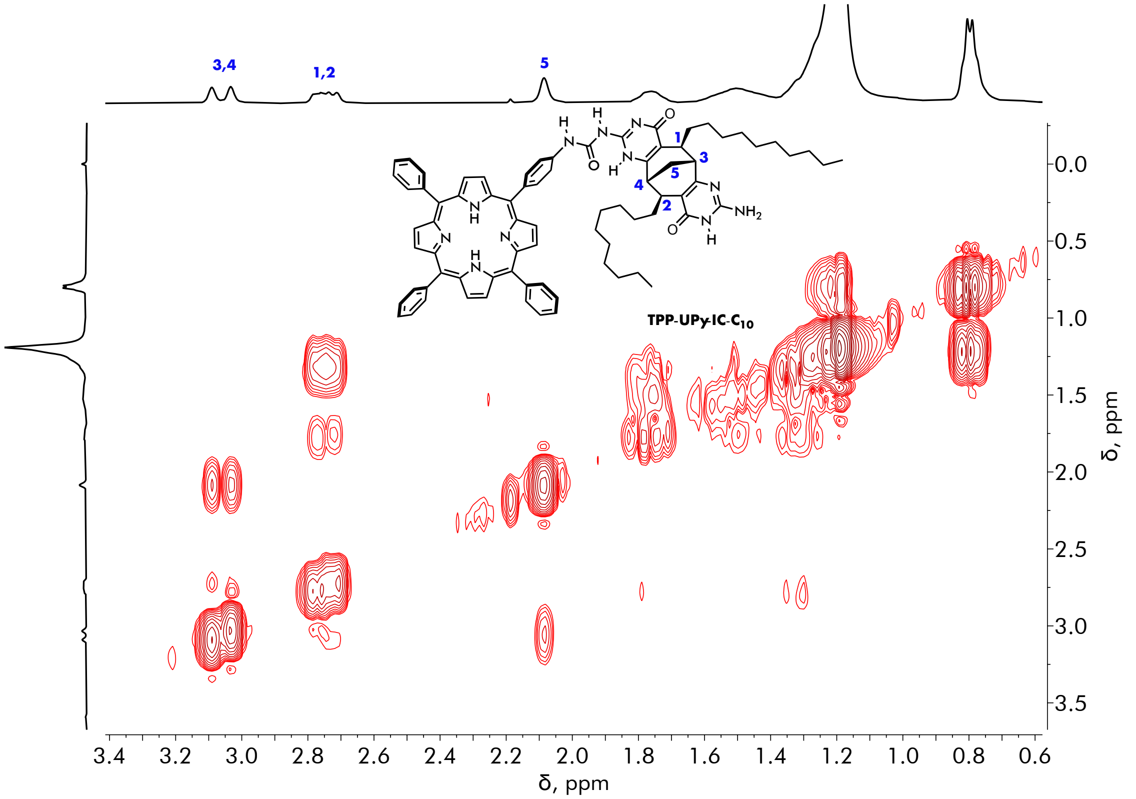

Figure S26. COSY ($CDCl_3$/TFA, 400 MHz) spectrum of **TPP-UPy-IC-C$_{10}$**.

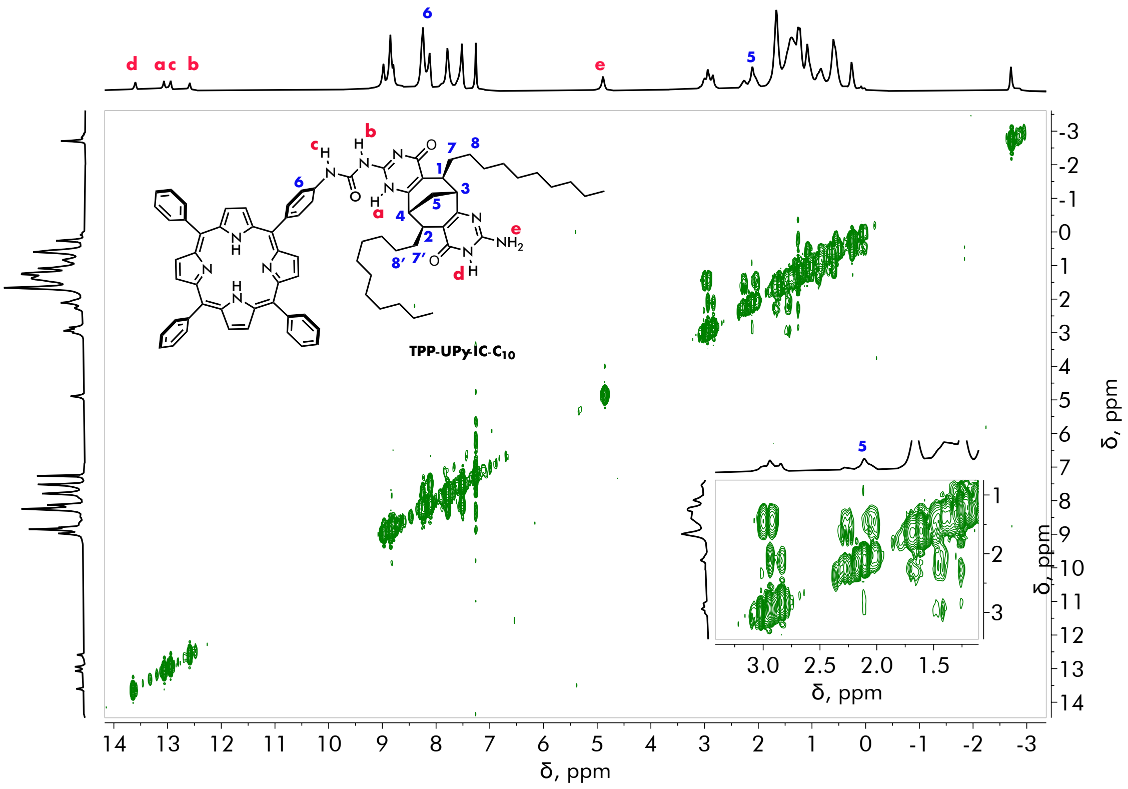

Figure S27. COSY ($CDCl_3$, 400 MHz) spectrum of (**TPP-UPy-IC-C10**)4.

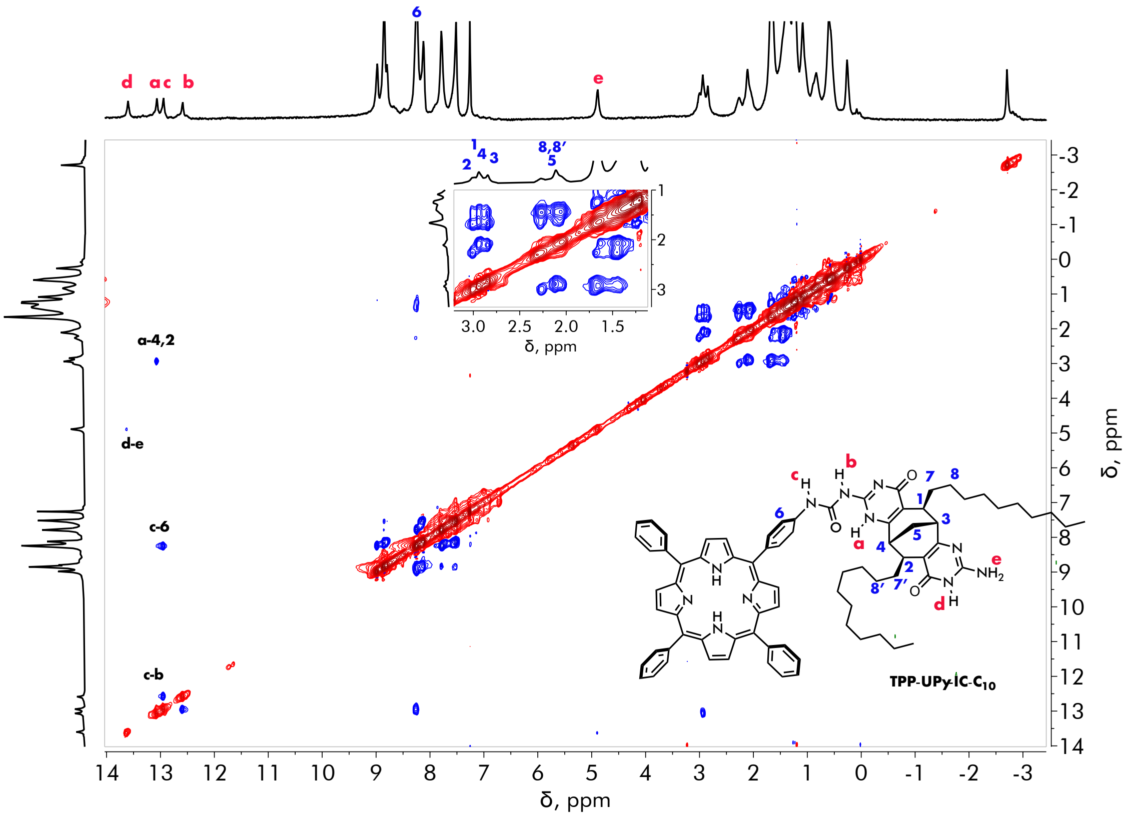

Figure S28. ROESY ($CDCl_3$, 400 MHz) spectrum of (**TPP-UPy-IC-C10**)4.

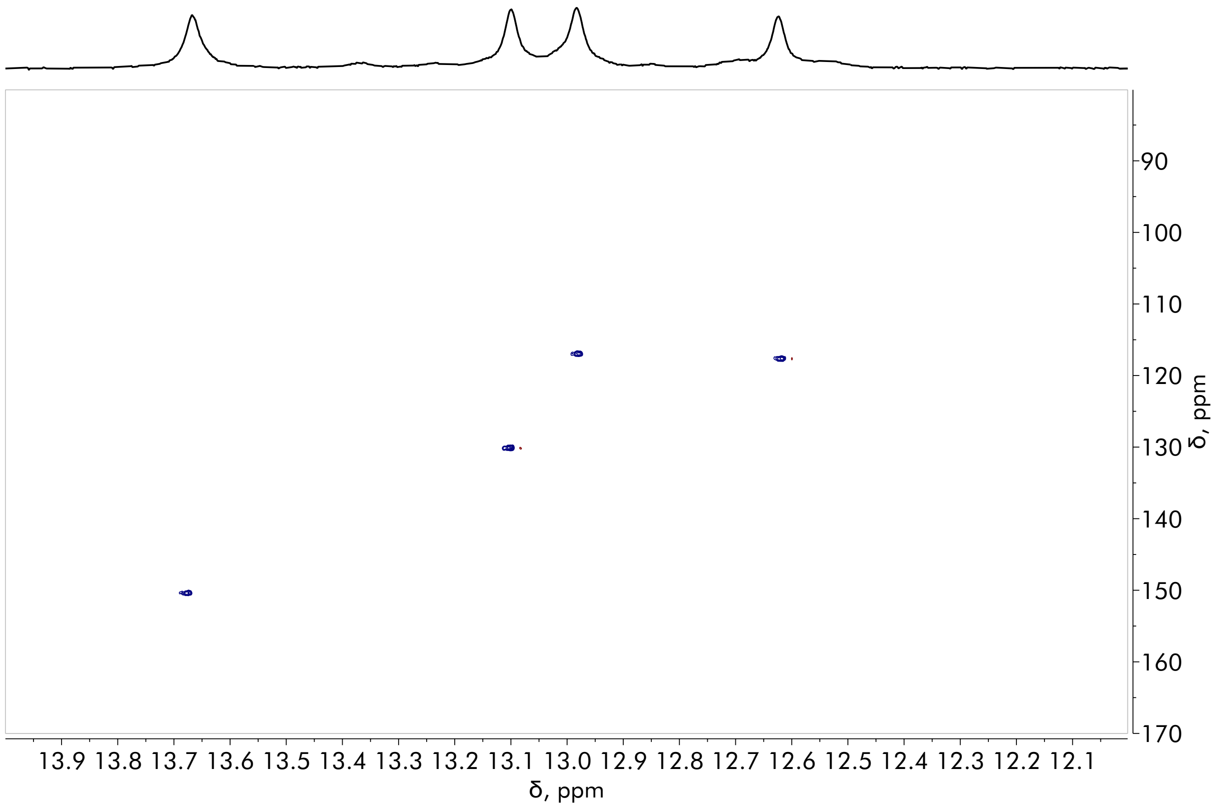

Figure S29. ^{1}H-^{15}N HSQC ($CDCl_3$, 400 MHz) spectrum of (**TPP-UPy-IC-C$_{10}$**)$_4$.

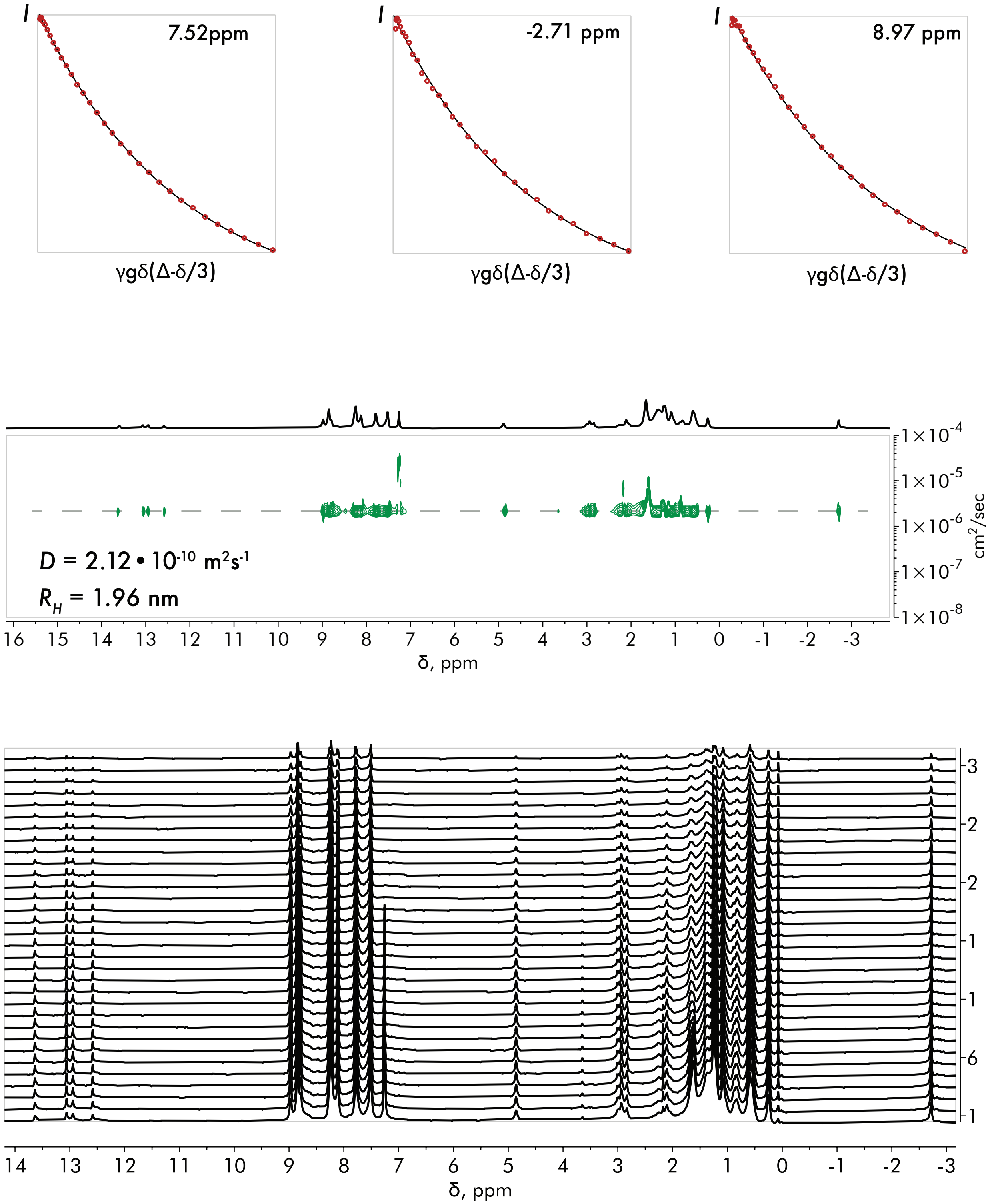

Figure S30. DOSY ($CDCl_3$, 400 MHz) spectrum of (**TPP-UPy-IC-C_{10}**)$_4$ (fitting of signal decay to Stejskal-Tanner equation (top), DOSY trace (middle) and stacked spectra for each data point (bottom)).

NMR characterization of C_{60}@(**TPP-UPy-IC-C$_{10}$**)$_4$ complex

H-bonding interfaces. The most downfield resonance **c** (δ = 13.30 ppm) (Figure S31) gives NOE cross-peaks with protons **b**, **6** and **2**. The absence of signal for proton **c** in the ^{1}H-^{15}N HSQC spectrum strongly suggest that the proton **c** is -O-H proton of the enolic form of UPy (Figure S32). The correlation with N-H proton **b** further supports its involvement in quadruple DADA-ADAD H-bonding array. Moreover, as indicated in molecular model, the distance between proton **c** and the proton **2**, is within 3.1-3.6 Å giving rise to the observed NOE dipole coupling (Figure S31b). The resonance at 12.69 ppm was assigned as proton **a** based on the cross-peaks with protons **1** and **6**. Protons **b** and **d** were assigned based on the NOE interaction of proton **b** with proton **c** as stated above, and also considering ^{15}N chemical shifts in the ^{1}H-^{15}N HSQC spectrum (Figure S33).

Bicyclic scaffold and C10 chains. Besides of highly clustered alkyl chain resonances, well separated signals were observed for allyl and bridgehead position protons in the region 3.10-2.30 ppm. The latter protons **3** and **4** were observed as apparent singlets at δ = 3.05 ppm and δ = 2.55 ppm, respectively, typical for these protons. Their assignment was made based NOE interaction in the ROESY spectrum. Namely, the correlation of proton **1** with proton **a** indicates that the left part of the overlapping multiplet belongs to UPy side of the bicyclic core. Likewise, proton **c** correlates with right side of the multiplet. The assignment of the bridgehead protons **3** and **4** can be made from the observed cross-peak of the allylic protons **1** and **2**, respectively. Both protons **3** and **4** also show scalar coupling with bridge protons **5** in COSY spectrum.

The assignment of C10 alkyl chain on the UPy side, involved in C-H···π interaction was based on the analysis of the COSY spectrum. Starting from the proton **1**, one may easily follow the connectivity along the chain (Figure S31). Proton **1** appears as well separated pair of diastereotopic protons, typical to protons at this position. Similarly, scalar correlation of the downfield protons eventually allows joining together two parts of the chain. Resonance at δ = -0.64 ppm shows only one-way scalar coupling and thus it must correspond to the terminal methyl group (protons **10**, Figure S31).

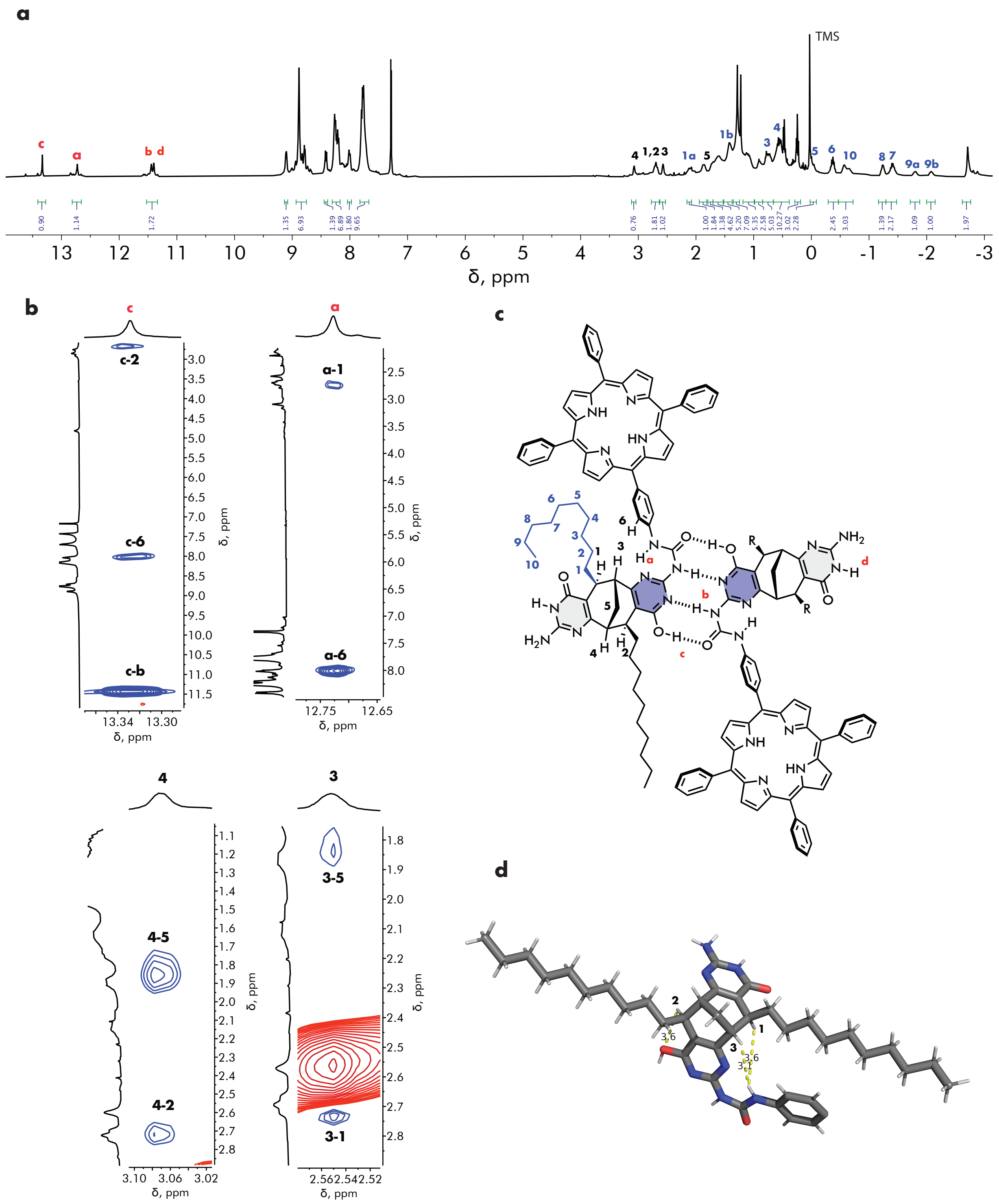

Figure S31. (a) ^{1}H NMR ($CDCl_3$, 400 MHz) of C_{60}@(**TPP-UPy-IC-C$_{10}$**)$_4$. Sample preparation: 4 mg of **TPP-UPy-IC-C$_{10}$** was dissolved in 0.55 mL $CDCl_3$. To this solution 0.25 equiv of C_{60} was added and the mixture was heated at 50 °C. Alternatively, the mixture can be left at room temperature for 48 hours with occasional shaking. (b) Selected parts of the ROESY spectrum of C_{60}@(**TPP-UPy-IC-C$_{10}$**)$_4$. (c) Schematic representation of dimeric unit with **UPy-UPy** H-bonding interface. (d) Molecular model of of **TPP-UPy-IC-C$_{10}$** monomer with distances (in Å) indicated.

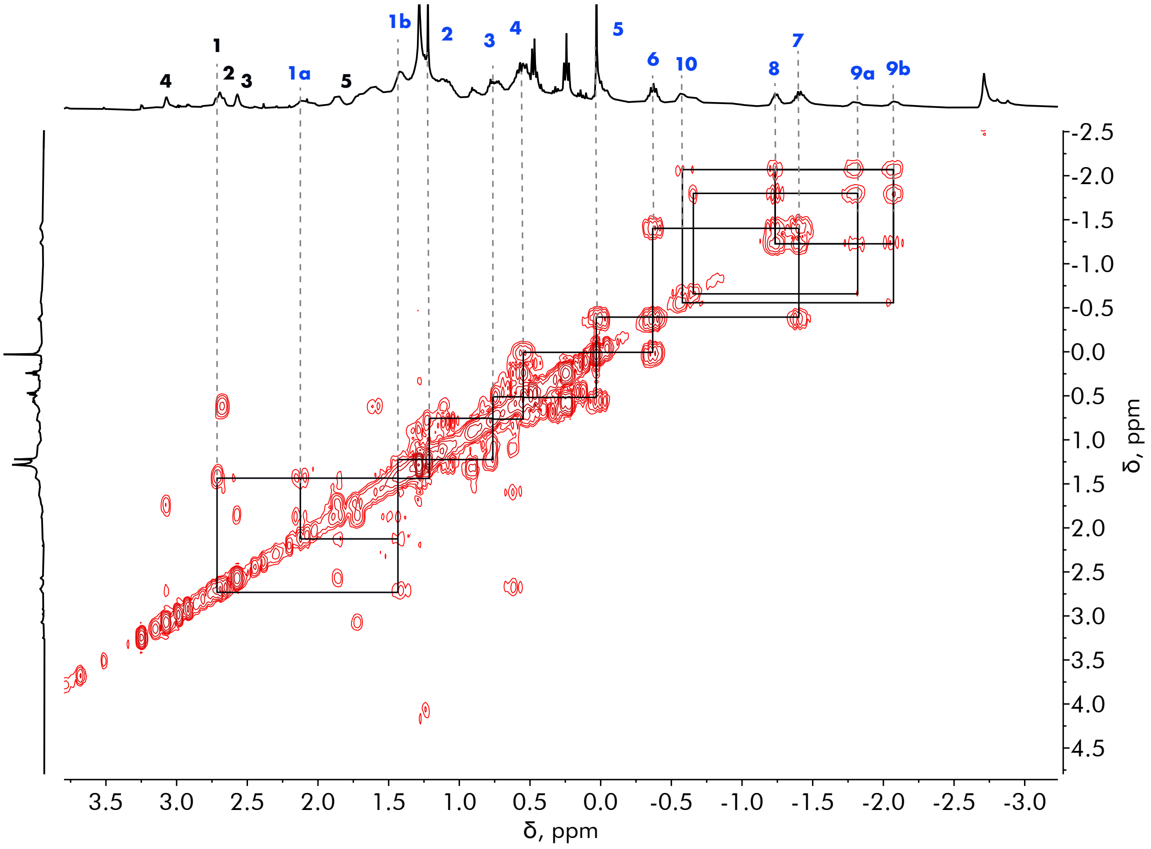

Figure S32. COSY ($CDCl_3$, 400 MHz) spectrum of C_{60}@(**TPP-UPy-IC-C$_{10}$**)$_4$.

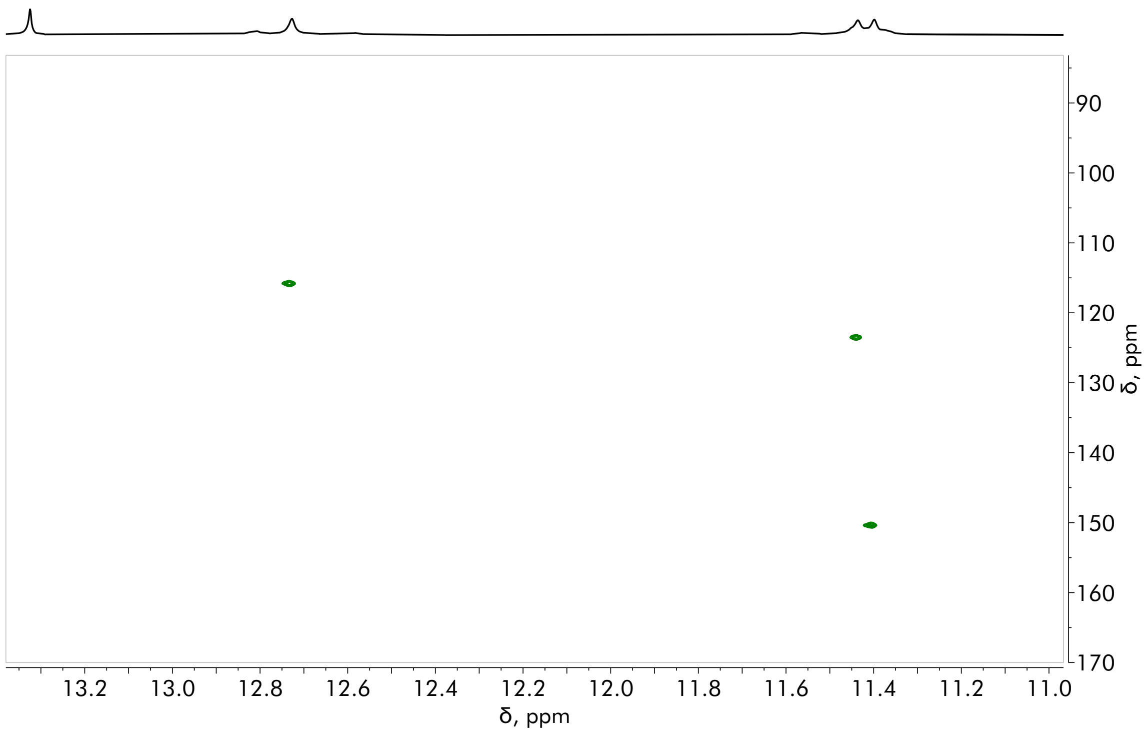

Figure S33. ^1H-^{15}N HSQC spectrum of C_{60}@(**TPP-UPy-IC-C$_{10}$**)$_4$.

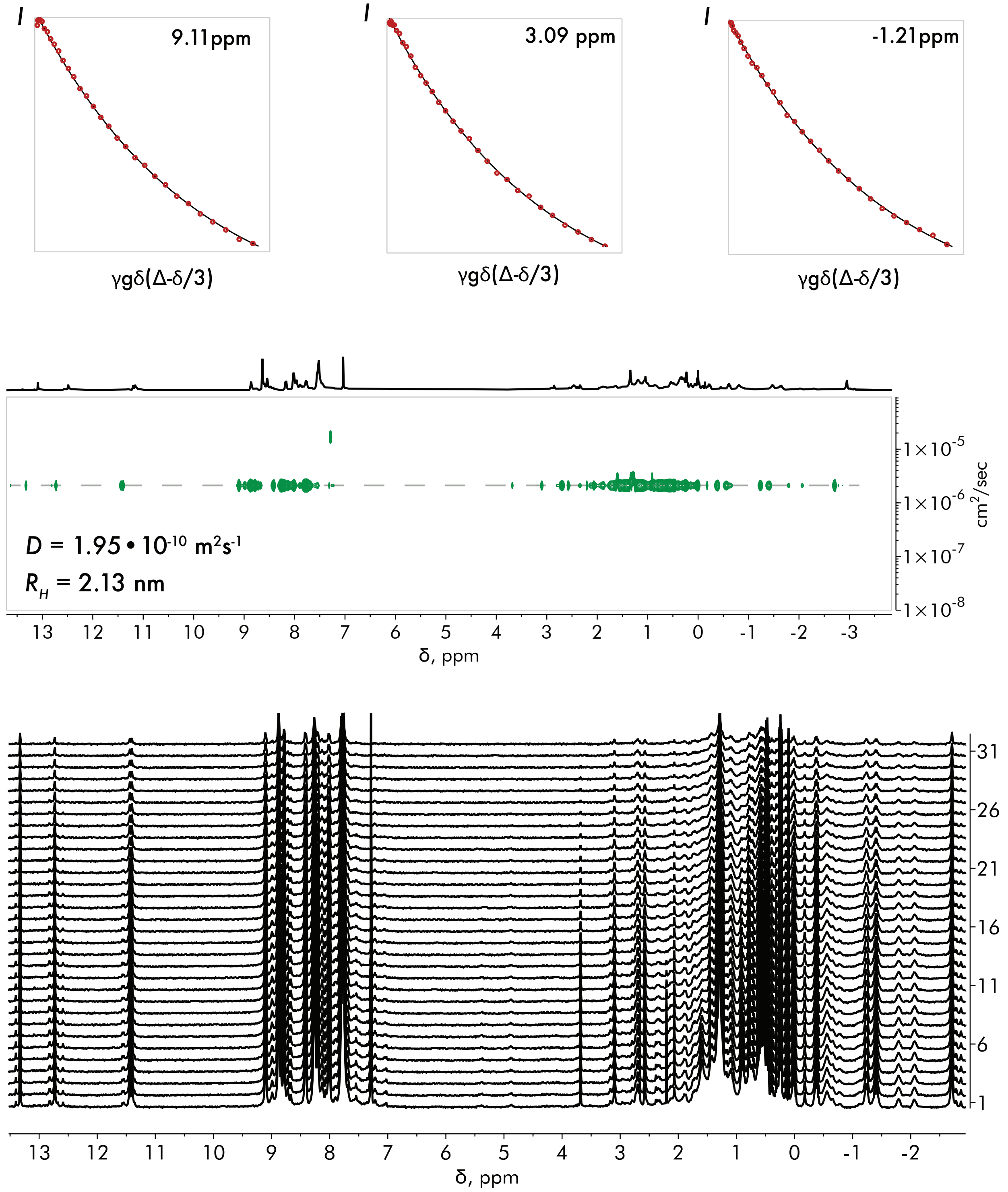

Figure S34. DOSY ($CDCl_3$, 400 MHz) spectrum of C_{60}@(**TPP-UPy-IC-C$_{10}$**)$_4$ (fitting of signal decay to Stejskal-Tanner equation (top), DOSY trace (middle) and stacked spectra for each data point (bottom)).

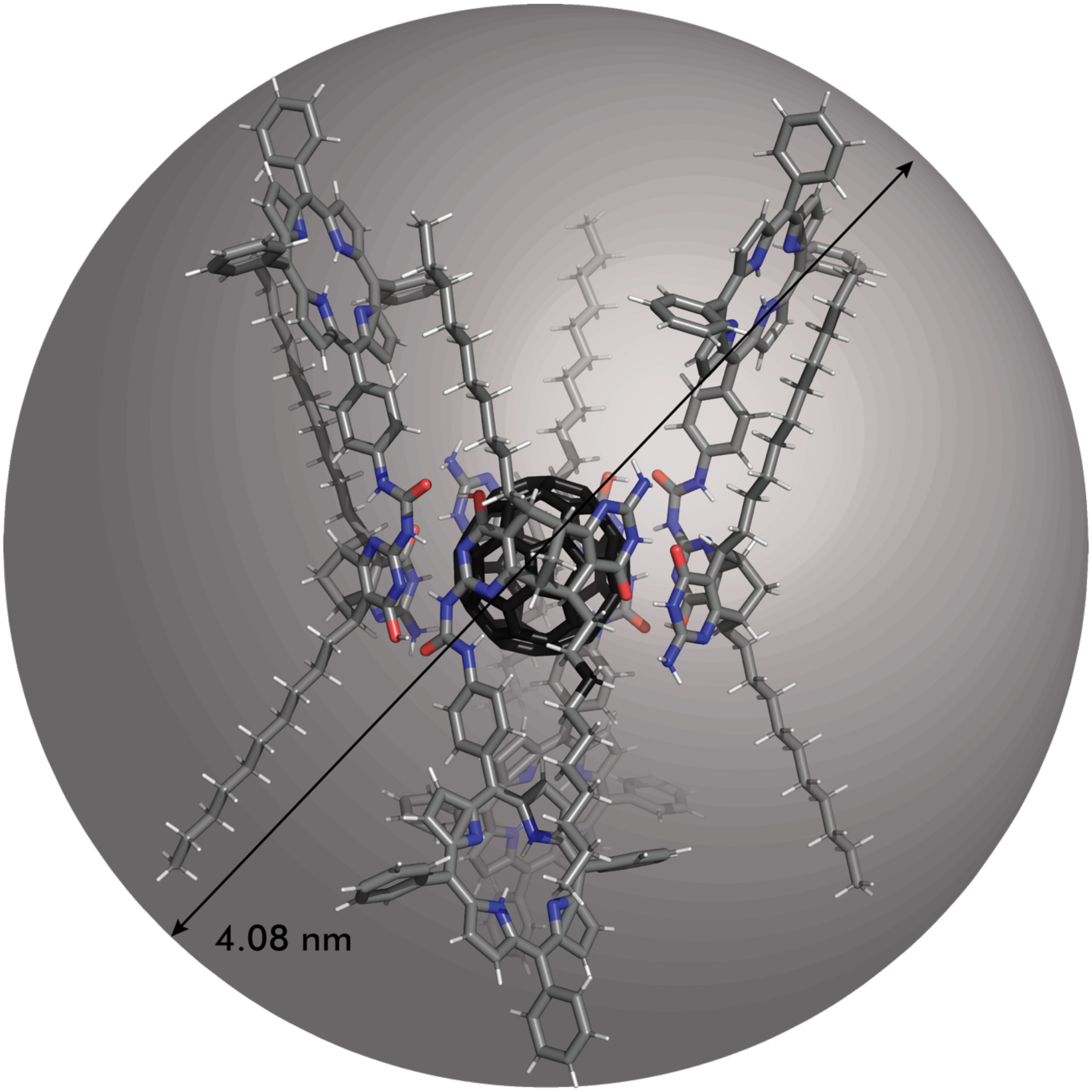

Figure S35. Molecular models (MM) of C_{60}@(**TPP-UPy-IC-C_{10}**)$_4$ tetramer and calculated maximum diameter.

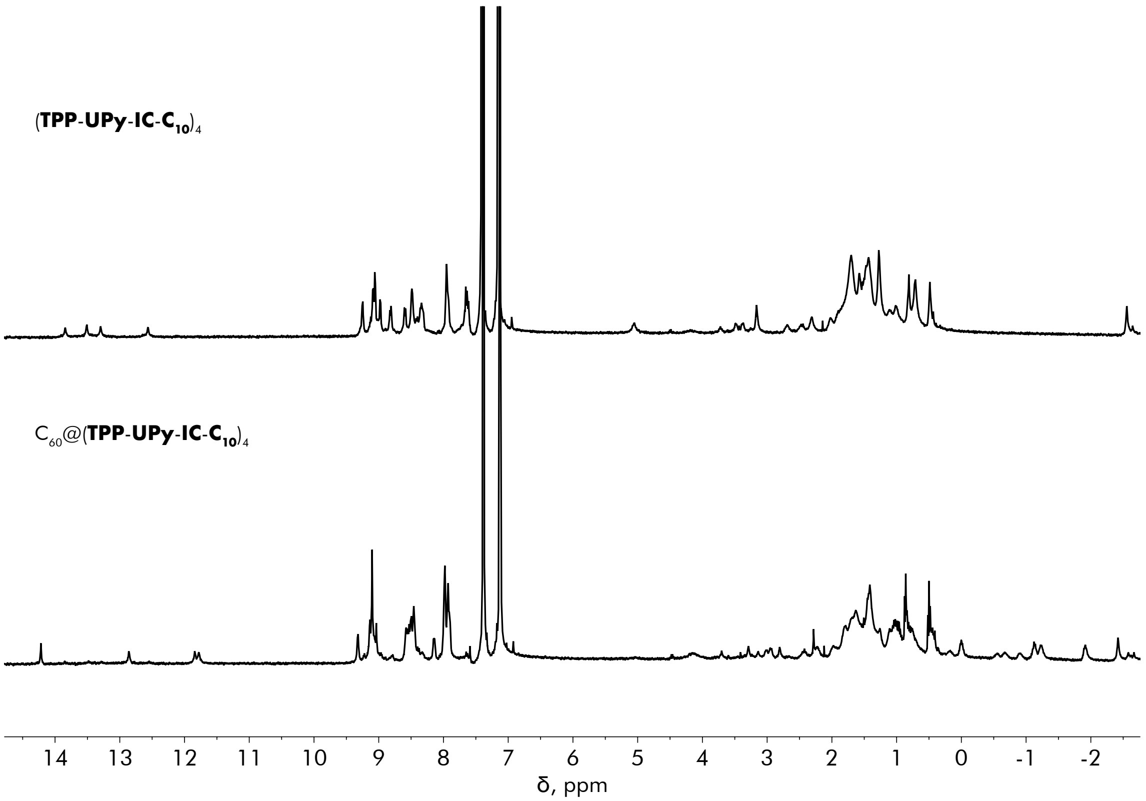

Figure S36. ¹H NMR (d_4-1,2-dichlorobenzene, 400 MHz) spectrum of (**TPP-UPy-IC-C$_{10}$**)$_4$ (top) and C_{60}@(**TPP-UPy-IC-C$_{10}$**)$_4$ (bottom). Sample preparation: 1.0 mg **TPP-UPy-IC-C$_{10}$** and 0.25 equiv of C_{60} was heated at 80 °C in d_4-1,2-dichlorobenzene (0.46 mL) overnight.

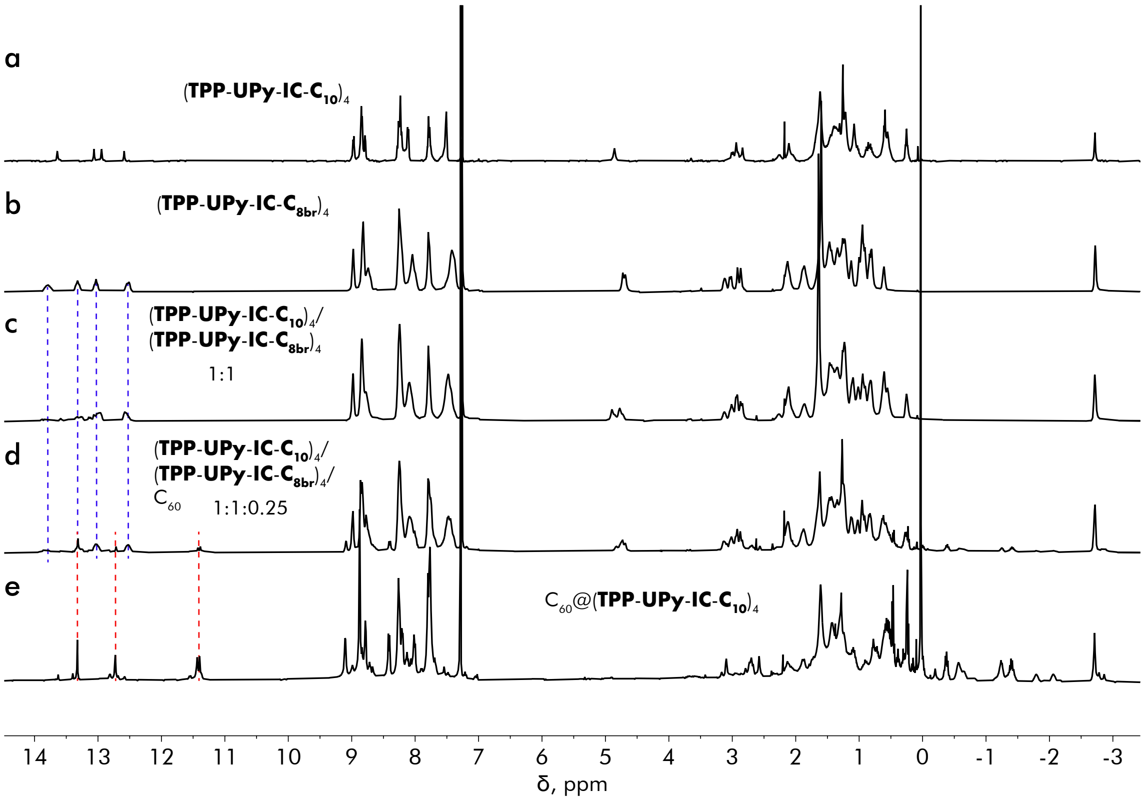

Figure S37. ¹H NMR ($CDCl_3$, 400 MHz) spectra of C_{60}-mediated self-sorting of **TPP-UPy-IC-C$_{10}$** and **TPP-UPy-IC-C$_{8br}$** monomers.

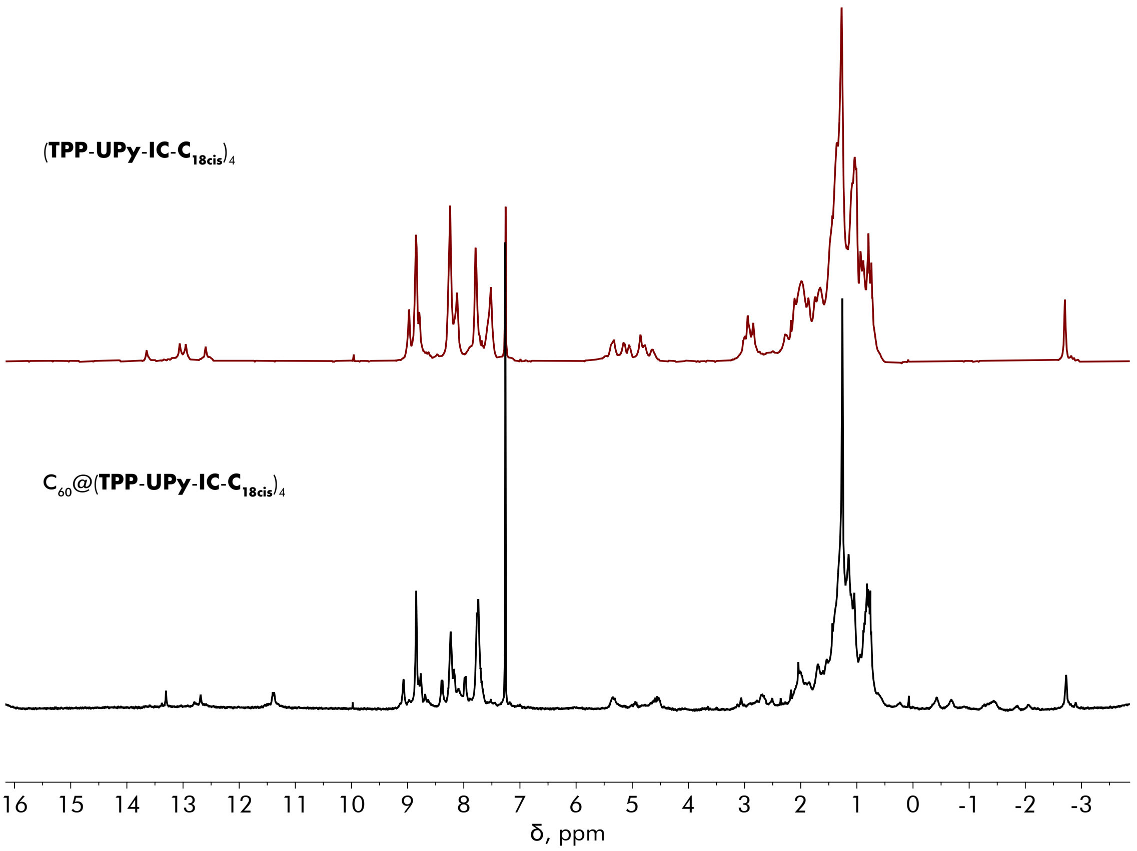

Figure S38. ^{1}H NMR ($CDCl_3$, 400 MHz) spectrum of (**TPP-UPy-IC-C$_{18cis}$**)$_4$ (top) and C_{60}@(**TPP-UPy-IC-C$_{18cis}$**)$_4$ (bottom).

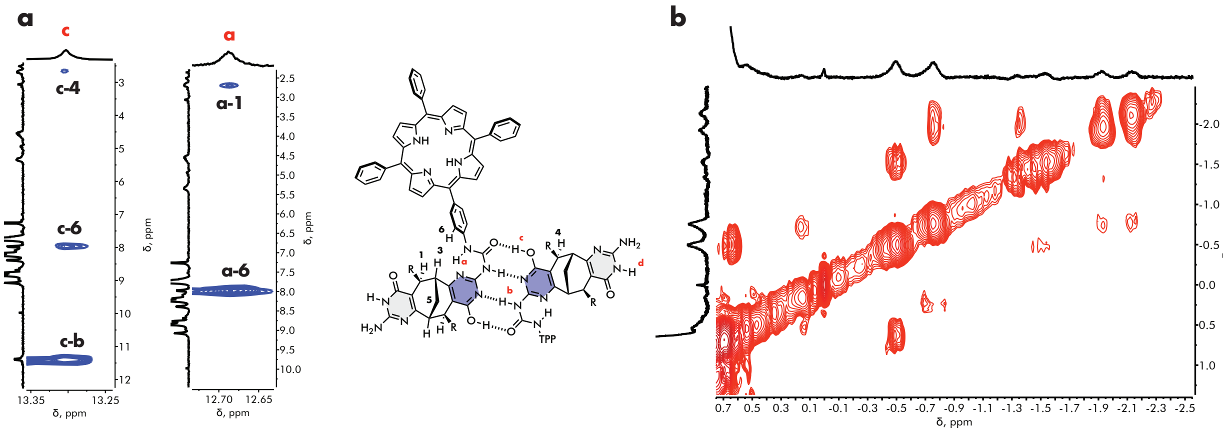

Figure S39. Part of ROESY (a) and COSY (b) spectrum of C_{60}@(**TPP-UPy-IC-C$_{18cis}$**)$_4$.

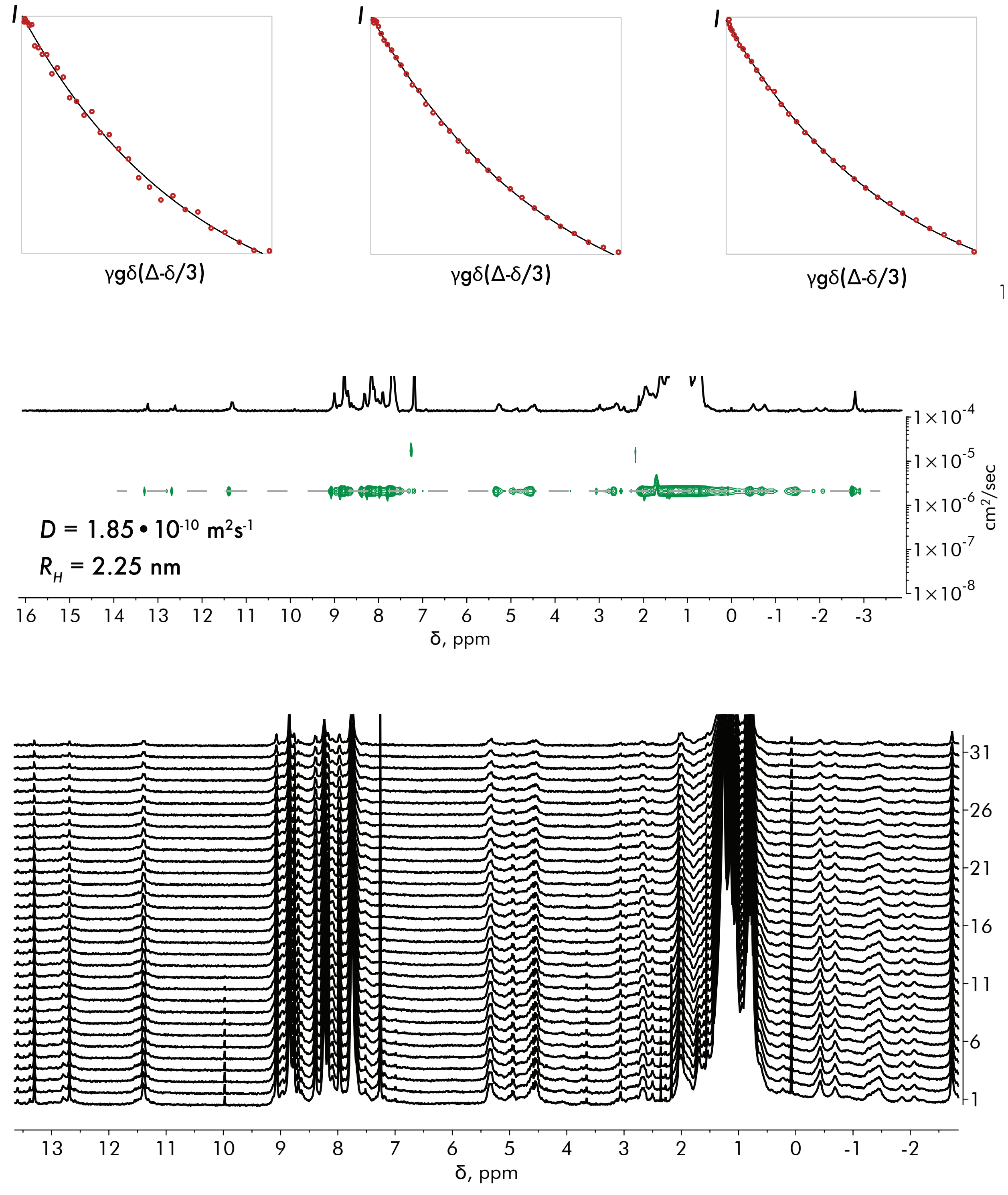

Figure S40. DOSY (CDCl3, 400 MHz) spectrum of C_{60}@(**TPP-UPy-IC-C$_{18cis}$**)$_4$.

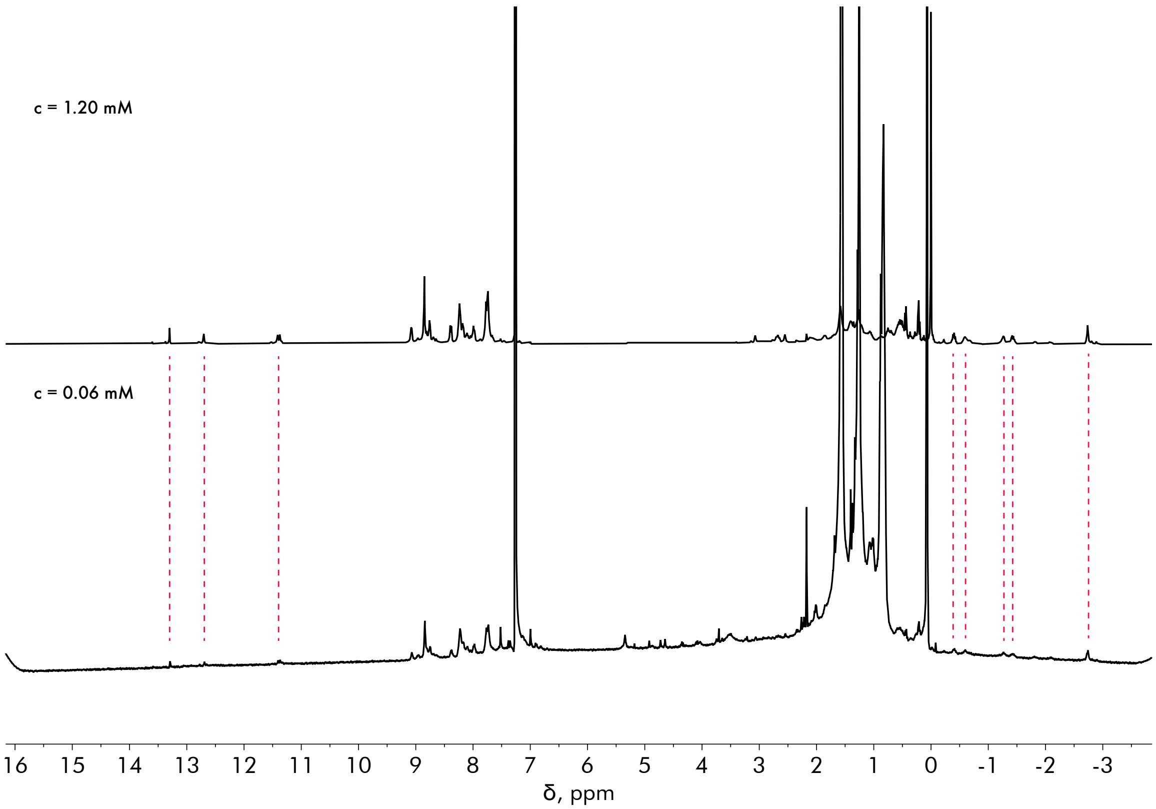

Figure S41. Dilution ^{1}H NMR ($CDCl_3$, 400 MHz) of C_{60}@(**TPP-UPy-IC-C$_{10}$**)$_4$. Resonances in the region $\delta = 2.0 - 5.5$ ppm are due to impurities from $CDCl_3$ and plastic syringe (number of scans NS = 20 000).

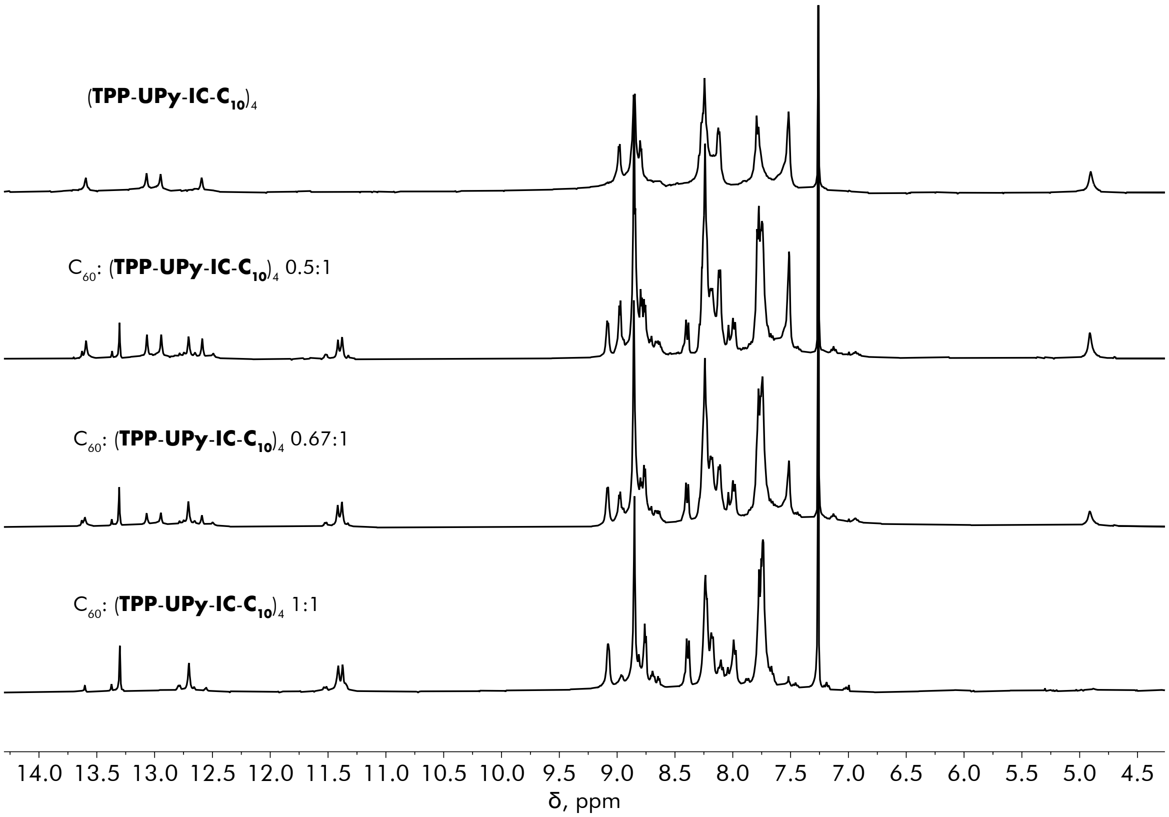

Figure S42. Titration^{1}H NMR ($CDCl_3$, 400 MHz) of (**TPP-UPy-IC-C$_{10}$**)$_4$ with C_{60}.

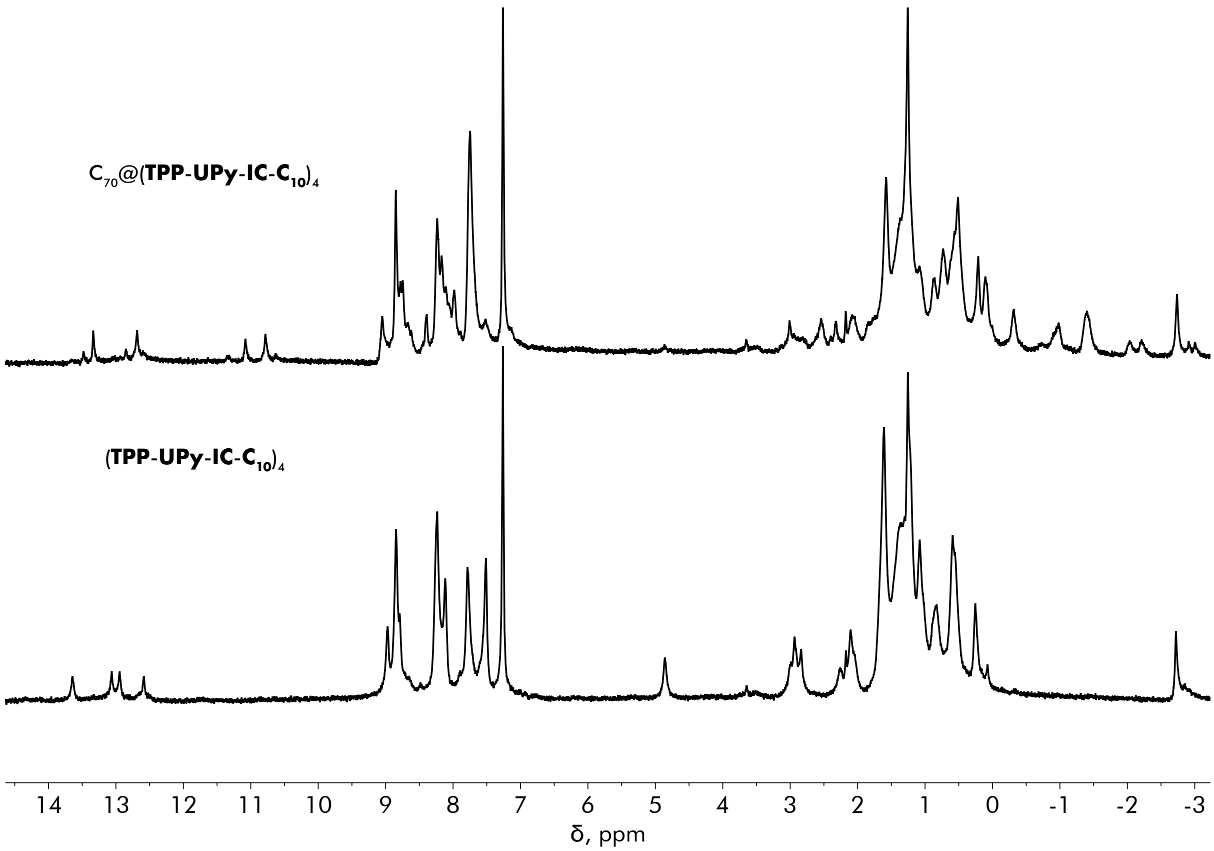

Figure S43. ^{1}H NMR ($CDCl_3$, 400 MHz) spectrum of (**TPP-UPy-IC-C$_{10}$**)$_4$ (bottom) and C$_{70}$@(**TPP-UPy-IC-C$_{10}$**)$_4$ (top). Sample preparation: 4.0 mg **TPP-UPy-IC-C$_{10}$** and 0.25 equiv of C$_{70}$ was heated at 50 °C in $CDCl_3$ (0.46 mL) for few hours.

Electron paramagnetic resonance spectroscopy

EXPERIMENTAL METHODS

All solvents were purchased from Sigma Aldrich at > 99% purity. Visible absorption spectra of the compounds in toluene: 2-MTHF (1:4) were acquired in 10 mm quartz cuvettes using a UV-Vis spectrophotometer (Cary 60, Agilent) (Figure S44).

Samples for Light-Induced Triplet–Triplet Electron Resonance (LITTER) spectroscopy were prepared at a porphyrin concentration of 80 µM in d_8-toluene: 2-MTHF (1:4) in 3.9 mm outer diameter (OD) quartz tubes and were degassed by 3 freeze-pump-thaw cycles in a Schlenk line.

Samples for Double Electron–Electron Resonance (DEER) spectroscopy were prepared at a Cu-porphyrin concentration of 320 µM in the same solvent mixture in 1.6 mm OD quartz tubes.

LITTER[S4] was performed at X-band (microwave frequency = 9.7 GHz) and a temperature of 20 K in a pulsed spectrometer (ElexSys E680, Bruker) using an over-coupled dielectric resonator (EN 4118X-MD5, Bruker) and an Oxford Instruments (CF935) cryostat. Laser 1 (OPOlette 355 SE, Opotek) was directed into the resonator through the optical window of the cryostat, while laser 2 (VersaScan OPO, GWU) was delivered via a 1 mm x 6 m optical fibre (FT1000-EMT, Thorlabs) directly inserted inside the sample tube.[S5] Both lasers were set at a wavelength of 517 nm, corresponding to the most intense maximum of the porphyrin Q-band region determined by UV-Vis , with 1 mJ per flash. Excitation in the Q-band region is preferred over excitation in the Soret band to prevent excess photo-bleaching of the sample. A pulse delay generator was used to externally trigger both lasers and the spectrometer at a repetition rate of 20 Hz and one shot per point, and the microwave pulses were moved forward in time with respect to the fixed laser flashes. LITTER traces were acquired using the pulse sequence shown in Figure S46 (a): laser 1 – DAF – $\pi/2 - \tau - \pi - \tau'$ – laser 2 – τ'' – echo,[S4] with a length of 12 ns for the π pulse. A $\tau = \tau' + \tau''$ = 1 µs was used and the DAF was incremented from an initial value of 2 µs in steps of 8 ns. The delay between flashes was t_{pp} = 7 µs. Measurements were carried out at two different values of the external magnetic field, (c.a. 339.6 and 385.8 mT).

DEER[S6] was performed at Q-band (microwave frequency = 33.9 GHz) and a temperature of 15 K in a pulsed spectrometer (ElexSys E580, Bruker) using an overcoupled dielectric resonator (EN 5107D2, Bruker) and an Oxford Instruments cryostat. DEER traces were acquired using the 4-pulse refocused-echo version of the experiment, shown in Figure S47 (a): $\pi/2 - \tau_1 - \pi - \tau' - \pi_{pump} - \tau'' - \pi - \tau_2$ – echo, with a length of 10 ns for the π detection pulse and 8 ns for the π pump pulse. Delays τ_1 = 0.2 µs and τ_2 = 1.5 µs were used, with $\tau_1 + \tau_2 = \tau' + \tau''$, and the pump pulse was moved with respect to the detection pulses in steps of 4 ns. Measurements were carried out at 7 different values of the external magnetic field, (c.a. 1081.5, 1105.9, 1130.3, 1154.7, 1167.0, 1179.2 and 1189.5 mT).

For both LITTER and DEER, the raw dipolar traces acquired at different field positions were phase- and background-corrected and averaged weighted by the corresponding spectral intensities to obtain an orientation-independent form factor, which was then analysed via Fourier Transform and Tikhonov Regularization using the Matlab® *DeerAnalysis2019* routine to

extract the corresponding distance distribution.[S7] A g-factor correction was applied to the determined distance distributions, as previously described, using the following expression[S8]

$$r_{corrected} = r\left(\frac{g_{eff,pump}\, g_{eff,det}}{2.0023^2}\right)^{\frac{1}{3}}$$

where $g_{eff,pump}$ and $g_{eff,det}$ are the effective g-values of the pump and detection spin centres, calculated as averages weighted by the corresponding spectral intensities.

Echo-detected field-swept spectra were acquired using a standard Hahn echo pulse sequence (π/2 – τ – π – τ – echo), in the same conditions as the LITTER and DEER experiments, respectively. In the former case, the Hahn echo sequence was preceded by a laser flash (517 nm, 2 mJ per flash, 20 Hz).

Time-resolved EPR (TR-EPR) was carried out at X-band and 20 K in a critically coupled resonator following laser excitation (517 nm, 2 mJ per flash, 20 Hz), without field modulation or phase sensitive detection. The signal was averaged between 0.5 and 0.8 μs after the laser flash, around the intensity maximum of the time trace.

COMPUTATIONAL METHODS

Spin density calculations were performed *in vacuo* using Gaussian® 16 (revision A.03).[S9] Electronic spin densities were obtained by single-point calculations using the B3LYP functional, with basis sets Def2SVP for H, C, N and O, and Def2TZVP for Cu. The spin multiplicity was set to doublet and triplet for CuTPP and TPP, respectively. The electronic spin densities in the C_{60} triplet state were approximated to be equally distributed over the 60 atoms for the purposes of spin-spin histogram generation. Atomic Mulliken spin densities were used in all cases.

Pulse excitation profiles were simulated using the Matlab® *EasySpin* toolbox.[S10] DEER dipolar spectra were simulated using the following expression for the dipolar frequency

$$\omega_{dd} = \frac{\mu_B^2 \mu_0 g_A g_B}{4\pi h\, r_{AB}^3}\left(1 - 3\cos^2\theta\right)$$

and the powder average factor $\sin\theta$, where $g_A = g_{eff,pump}$, $g_B = g_{eff,det}$, r_{AB}^3 is the spin-spin distance, and θ is the angle between the static magnetic field and the spin-spin vector.[S8]

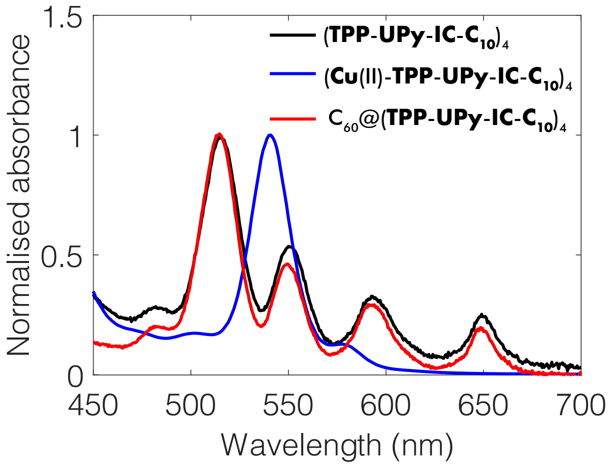

Figure S44. Visible absorption spectra of the empty cage (black), Cu-metallated empty cage (blue) and cage with C_{60} (red) recorded at room temperature.

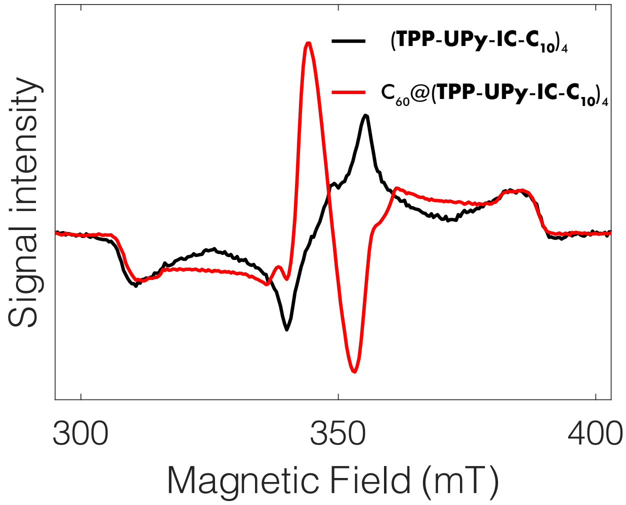

Figure S45. Time-resolved EPR spectra of the empty cage (black) and cage with C_{60} (red) acquired after laser excitation at 517 nm. Both spectra have been normalised to the intensity of the high-field shoulder. Differences arise between the two spectra due to the presence of the C_{60} triplet spectrum in the C_{60}@(**TPP-UPy-IC-C$_{10}$**)$_4$ sample.

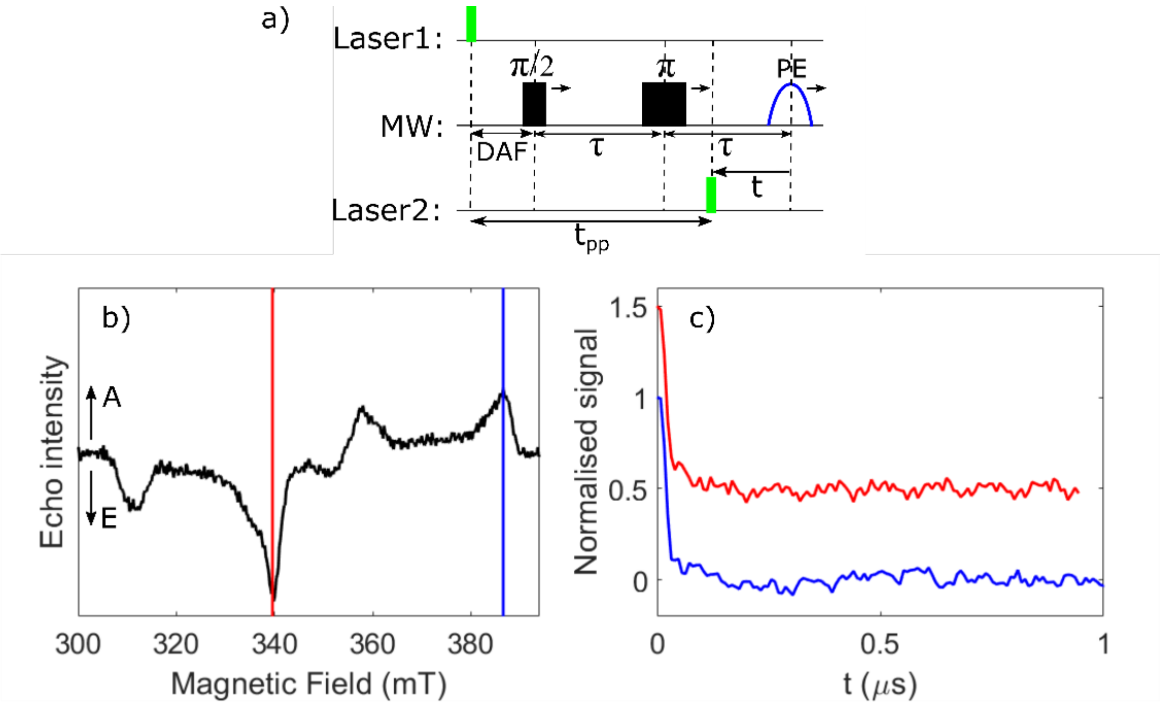

Figure S46. (a) LITTER sequence. (b) Echo-detected field-swept EPR spectrum of the tetraporphyrin cage with C_{60} measured after laser excitation at 517 nm, showing the field positions where LITTER traces were acquired (vertical lines). (c) Background-corrected and modulation depth-normalised LITTER traces acquired at the indicated field positions.

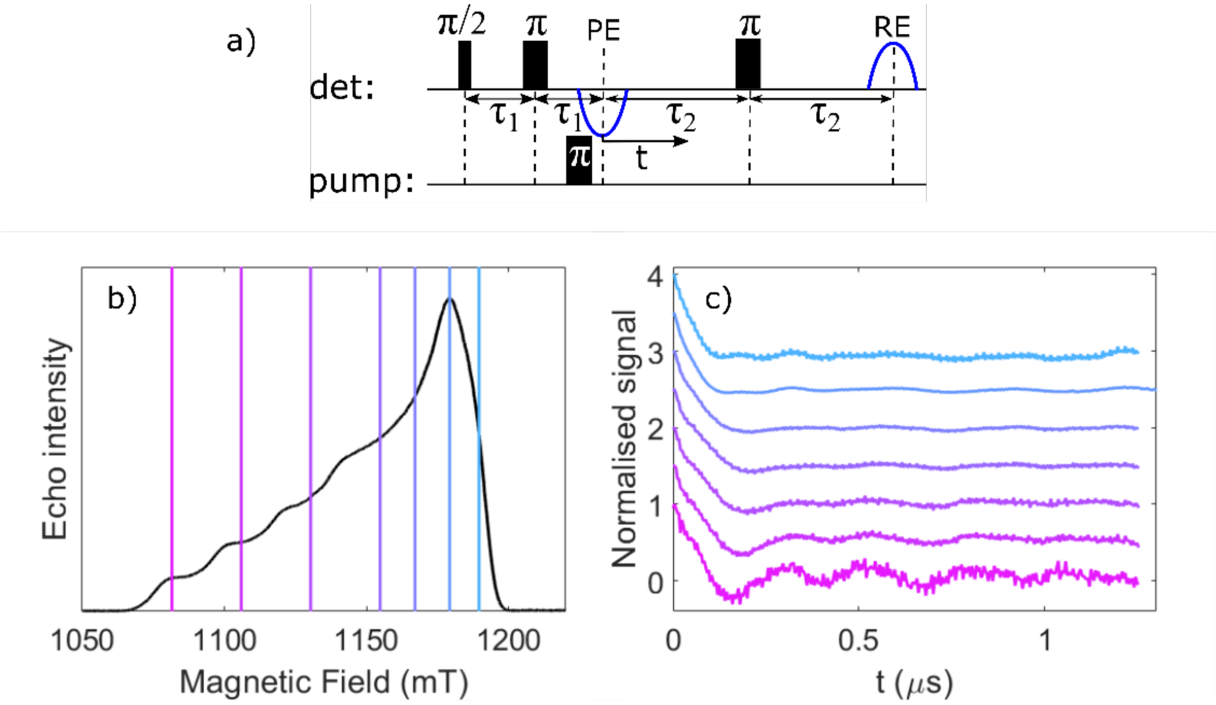

Figure S47. (a) 4-pulse DEER sequence. (b) Echo-detected field-swept EPR spectrum of the metalated cage measured in the dark, showing the field positions where DEER traces were acquired (vertical lines). (c) Background-corrected and modulation depth-normalised DEER traces acquired at the indicated field positions.

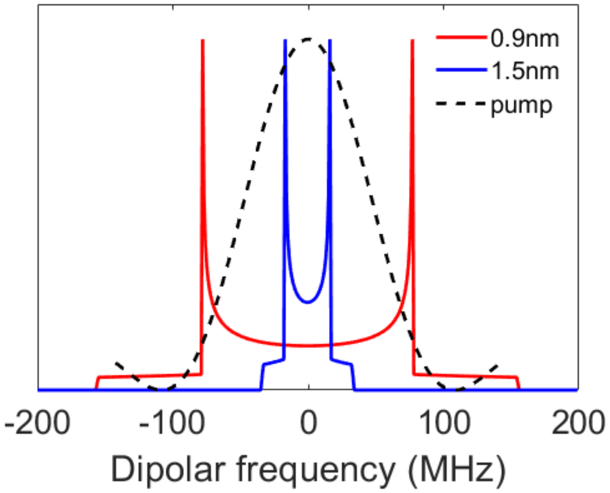

Figure S48. Simulated DEER dipolar spectra for spin-spin distances of 0.9 nm (red, corresponding to the left maximum of the histogram in Fig. 6 (d)) and 1.5 nm (blue, corresponding to the left maximum of the DEER-derived distance distribution in Fig. 6 (d)). The excitation profile of the 8 ns pump pulse used in the DEER experiment is shown as dashed line. The Pake pattern for the 0.9 nm distance will be significantly supressed at the edges by the pulse bandwidth and the resulting form will likely be interpreted as a slightly longer distance in an analysis using the DeerAnalysis 2019 routine.

Photophysical studies

Steady-state absorption spectra were recorded on a UV–vis–NIR spectrophotometer Lambda 950 (Perkin-Elmer). Fluorescence transients of the films were measured by using a time-correlated single-photon counting system PicoHarp 300 (PicoQuant), which utilized a pulsed semiconductor laser diode (repetition rate – 2.5 MHz, pulse duration - 70 ps, emission wavelength - 510 nm) as an excitation source. Fluorescence quantum yield was estimated by utilizing relative comparative method and using an integrating sphere (Sphere Optics) coupled to the CCD spectrometer PMA-12 (Hamamatsu) via an optical fiber, the 150 W xenon arc lamp (LOT-Oriel) coupled to a single monochromator 9030 (Sciencetech) was used as an excitation source.

Femtosecond transient absorption measurements were carried out using Harpia spectrometer pumped with Pharos-SP laser and Orpheus parametric amplifier system (Light Conversion). Samples were excited with 650 nm pulses (repetition rate 10 kHz, pulse duration 190 fs). Probe source was white light continuum pulses generated by focusing the 1030 nm pulses in purified water flowing inside quartz cuvette coupled to home-build flow system.

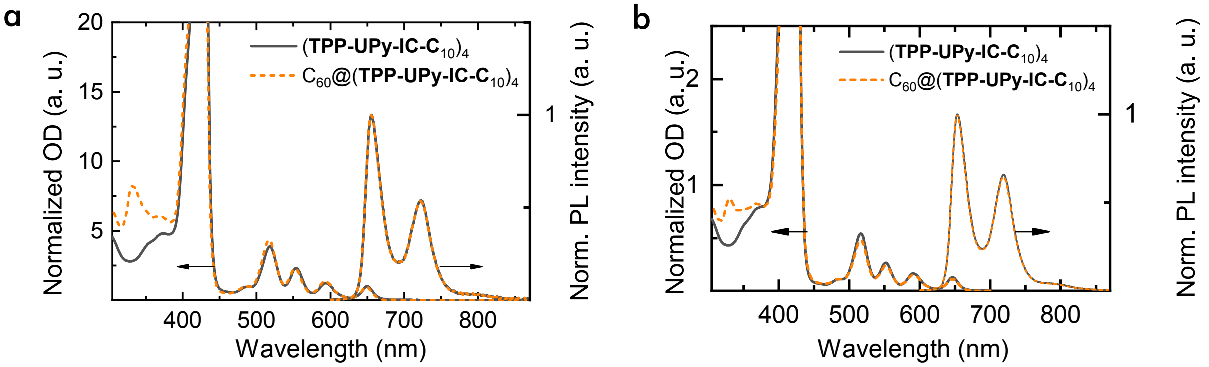

Figure S49. UV-vis and fluorescence spectra of (**TPP**-**UPy**-**IC**-$\mathbf{C_{10}}$)$_4$ and C_{60}@(**TPP**-**UPy**-**IC**-$\mathbf{C_{10}}$)$_4$ in 1,2-dichlorobenzene (a) and chloroform (b).

Fluorescence quantum yield was calculated using relative comparative method. Sulforhodamine-101 showing Φ_F=100% was used as a reference. Φ_F was calculated by:

$$\Phi_{\mathrm{F}} = \Phi_{\mathrm{F}}^{\mathrm{ref}}\left(\frac{I}{I_{\mathrm{ref}}}\right)\left(\frac{1-10^{-OD_{\mathrm{ref}}}}{1-10^{-OD}}\right)\left(\frac{n}{n_{\mathrm{ref}}}\right)^2$$

where *I* is spectral integral, *OD* – optical density, *n* – refractive index. Ref. denotes reference.

Table S1. Parameters of emission quantum yield measurements by relative comparative method.

	λ_{ex} nm	λ_{em} nm	*I*	*OD*	Solvent	*n*	Φ_F %

Reference:							
Sulforhodamine-101	550	595	$7.24 \cdot 10^6$	0.05	ethanol	1.36	100
Sample:							
$(TPP\text{-}UPy\text{-}IC\text{-}C_{10})_4$	550	653	$1.56 \cdot 10^6$	0.26	chloroform	1.45	**5.9**
$C_{60}@(TPP\text{-}UPy\text{-}IC\text{-}C_{10})_4$	550	653	$7.95 \cdot 10^5$	0.16	chloroform	1.45	**4.4**
$(TPP\text{-}UPy\text{-}IC\text{-}C_{10})_4$	550	656	$6.72 \cdot 10^5$	0.11	1.2-dichlorobenzene	1.55	**6.0**
$C_{60}@(TPP\text{-}UPy\text{-}IC\text{-}C_{10})_4$	550	656	$3.53 \cdot 10^5$	0.12	1.2-dichlorobenzene	1.55	**2.9**

λ_{ex} – excitation wavelength, λ_{em} – emission peak, OD – optical density, n – refractive index, Φ_F – quantum yield.

Additionally, Φ_F values were confirmed by utilizing the integrating sphere method.

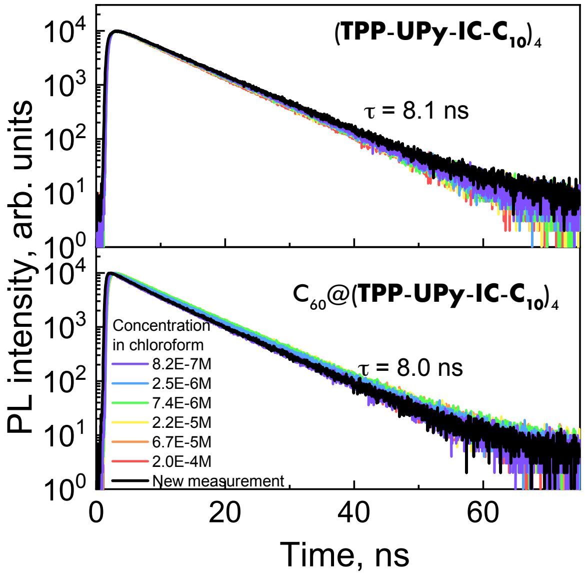

Figure S50. Fluorescence transients of (**TPP-UPy-IC-C$_{10}$**)$_4$ (top) and C_{60}@(**TPP-UPy-IC-C$_{10}$**)$_4$ (bottom) in chloroform.

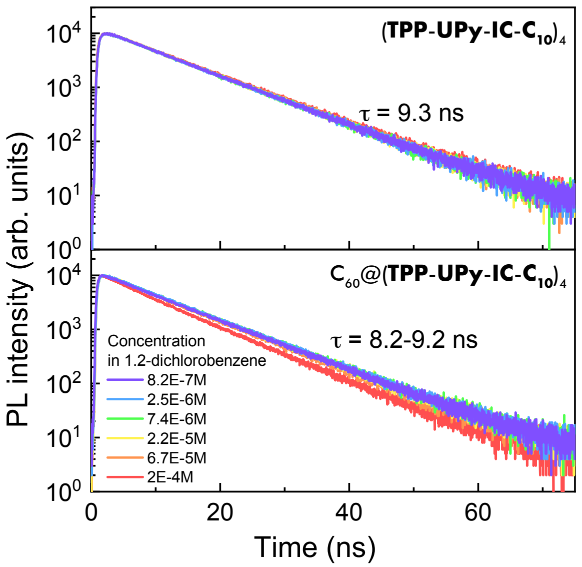

Figure S51. Fluorescence transients of (**TPP-UPy-IC-C$_{10}$**)$_4$ (top) and C_{60}@(**TPP-UPy-IC-C$_{10}$**)$_4$ (bottom) in 1,2-dichlorobenzene .

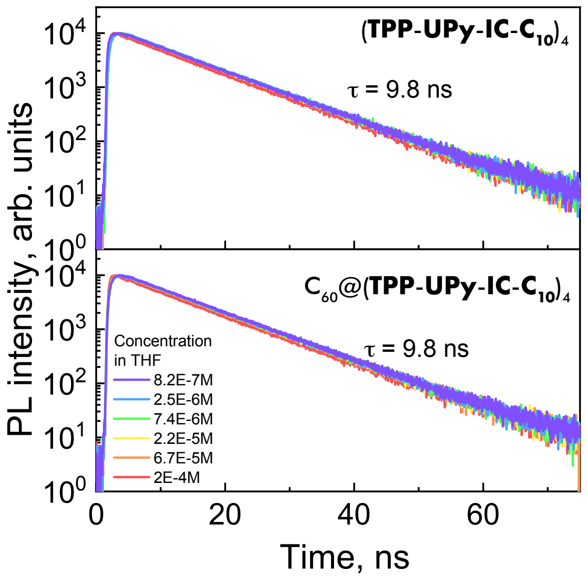

Figure S52. Fluorescence transients of (**TPP-UPy-IC-C$_{10}$**)$_4$ (top) and C_{60}@(**TPP-UPy-IC-C$_{10}$**)$_4$ (bottom) in THF.

Transient absorption

Transient absorption spectroscopy was employed to visualize energy and electron transfer processes in molecular complexes. Figure S53a shows transient absorption spectra of (**TPP-UPy-IC-C**$_{10}$)$_4$ in 1,2-dichlorobenzene solution, which indicate no excited state changes during the measured delay time window. The addition of C_{60} to (**TPP-UPy-IC-C**$_{10}$)$_4$ results in some energy transfer to new excited state species at later delay times (Figure S53b). Estimated 80 ps risetime visible in the transients of C_{60}@(**TPP-UPy-IC-C**$_{10}$)$_4$ (Figure S53c) could indicate the development of singlet state related to C_{60}. The following decay with the estimated lifetime of

1 ns suggests that further energy transfer occurs from singlet state to the triplet state of C_{60}. This is supported by spectral feature at 750 nm (Figure S53b) that does not decay with the rest of spectra and agrees with triplet excited state absorption spectra of C_{60} found in the literature.[S11-S12] Nevertheless, energy transfer in this system is very inefficient. Less pronounced differences were recorded between excitation dynamics of (**TPP-UPy-IC-C**$_{10}$)$_4$ monomer and C_{60}@(**TPP-UPy-IC-C**$_{10}$)$_4$ complex in chloroform solutions (Figure S54). Here, chloroform environment resulted in faster decay dynamics of both aggregates (Figure S54c) compared to 1,2-dichlorobenzene environment (Figure S53c). However, still faster decay of C_{60}@(**TPP-UPy-IC-C**$_{10}$)$_4$ complex could indicate energy transfer to C_{60}.

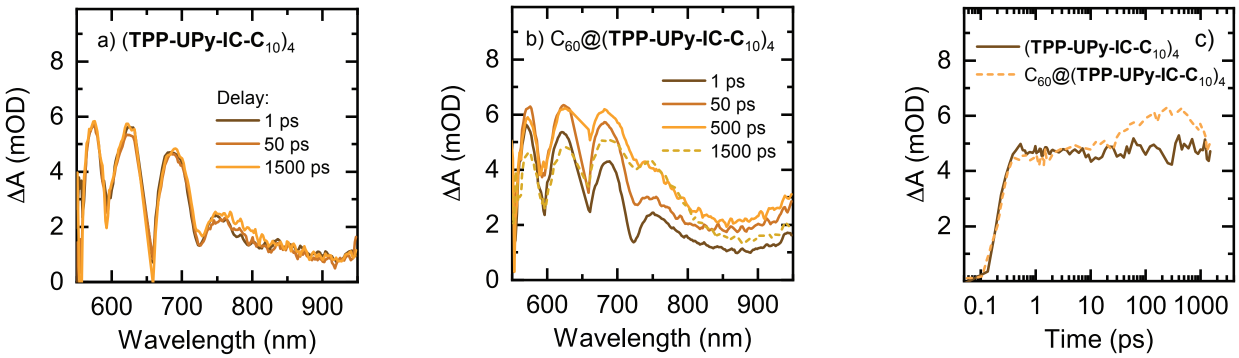

Figure S53. Transient absorption spectra at different delay times of (a) (**TPP-UPy-IC-C**$_{10}$)$_4$ and (b) C_{60}@(**TPP-UPy-IC-C**$_{10}$)$_4$ in 1,2-dichlorobenzene solutions. (c) Transients were recorded at 680 nm spectral position. Excitation wavelength was set to 650 nm for absorption at lowest **TPP** band. C_{60}@(**TPP-UPy-IC-C**$_{10}$)$_4$ sample concentration was the same as for sample in Figure S36 (c = 1.6 mM), to ensure quantitative formation of the complex.

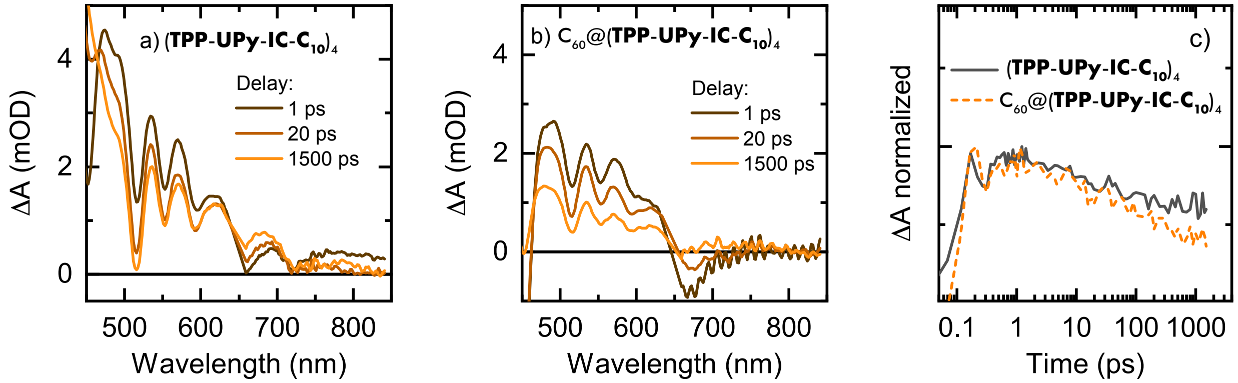

Figure S54. Transient absorption spectra at different delay times of (a) (**TPP-UPy-IC-C**$_{10}$)$_4$ and (b) C_{60}(**TPP-UPy-IC-C**$_{10}$)$_4$ in chloroform solutions. (c) Transients were recorded at 535 nm spectral position. Excitation wavelength was set to 650 nm for absorption at lowest **TPP** band.

Computational studies

The geometry of (**TPP-UPy-IC-C**$_{10}$)$_4$ and C_{60}@(**TPP-UPy-IC-C**$_{10}$)$_4$ was optimized using molecular mechanics as implemented in Spartan 15.

The conformational energy differences are calculated by Gaussian 16 software suite. The energy is evaluated by using the B3LYP hybrid exchange-correlation functional[S13] together with D3 version of Grimme's dispersion with Becke-Johnson damping.[S14] Pople-style 6-31G(d,p) basis set with polarization functions (d,p) on all atoms was used to expand electronic wavefunction. The Polarizable Continuum Model (PCM) using the integral equation formalism variant (IEFPCM) set to database entry for chloroform was used to model the solvent environment.
The center of mass between the decyl chain and porphyrin ring was used as a reaction coordinate to represent the energy differences. The energy difference between limiting conformations of decyl group with respect to its proximity to porphyrin ring was estimated to be -57 kJ/mol, therefore favoring the C-H … π interaction.

In order to study the ring-current effects on magnetic shielding, the nucleus independent chemical shifts (NICS) [S15-S23] were evaluated on a lattice from −9.0 to +9.0 Å for x- and y-axes and from -2.5 to 2.5 Å for the z-axis with a pitch of 0.1 Å above the π-plane of the porphyrin anion. The out-of-plane components of $NICS(1)_{zz}$, are plotted as a contour map in Figure 3. The system geometry was optimized by the density functional theory (DFT) using the 6–31G(d,p) basis set and the three-parameter Becke-Lee–Yang–Parr (B3LYP) hybrid exchange-correlation functional as implemented in the Gaussian 16 suite of programs.[S9] The energetic minimum was confirmed by performing vibrational analysis. The NICS values were calculated at the same B3LYP/6-31G(d,p) level with gauge-including atomic orbitals (GIAOs).

References

S1. Iglesias, B. A.; Hörner, M.; Toma, H. E.; Araki, K. 5-(1-(4- Phenyl)-3-(4-Nitrophenyl)Triazene)-10,15,20- Triphenylporphyrin: A New Triazene-Porphyrin Dye and Its Spectroelectrochemical Properties. *J. Porphyr. Phthalocyanines* **2012**, *16*, 200–209.

S2. Matthews, S. E.; Pouton, C. W.; Threadgill, M. D. Monofunctional Electrophilic and Nucleophilic Derivatives of Meso-Tetraphenylporphyrin for Attachment to Peptides. *J. Chem. Soc. Chem. Commun.* **1995**, *17*, 1809–1811.

S3. Račkauskaitė, D.; Gegevičius, R.; Matsuo, Y.; Wärnmark, K.; Orentas, E. An Enantiopure Hydrogen-Bonded Octameric Tube: Self-Sorting and Guest-Induced Rearrangement. *Angew. Chem. Int. Ed.* **2016**, *55*, 208–212.

S4. Bertran, A.; Henbest, K. B.; De Zotti, M.; Gobbo, M.; Timmel, C. R.; Di Valentin, M.; Bowen, A. M. Light-Induced Triplet–Triplet Electron Resonance Spectroscopy. *J. Phys. Chem. Lett.* **2021**, *12*, 80–85.

S5. Bertran, A.; Barbon, A.; Bowen, A. M.; Di Valentin, M. Light-Induced Pulsed Dipolar EPR Spectroscopy for Distance and Orientation Analysis. In *Methods in Enzymology*; Elsevier Inc., **2022**; Vol. 666, pp 171–231.

S6. Pannier, M.; Veit, S.; Godt, A.; Jeschke, G.; Spiess, H. W. Dead-Time Free Measurement of Dipole–Dipole Interactions between Electron Spins. *J. Magn. Reson.* **2000**, *142*, 331–340.

S7. Jeschke, G.; Chechik, V.; Ionita, P.; Godt, A.; Zimmermann, H.; Banham, J.; Timmel, C. R.; Hilger, D.; Jung, H. DeerAnalysis2006 - A Comprehensive Software Package for Analyzing Pulsed ELDOR Data. *Appl. Magn. Reson.* **2006**, *30*, 473–498.

S8. Bowen, A. M.; Jones, M. W.; Lovett, J. E.; Gaule, T. G.; McPherson, M. J.; Dilworth, J. R.; Timmel, C. R.; Harmer, J. R. Exploiting Orientation-Selective DEER: Determining Molecular Structure in Systems Containing Cu(II) Centres. *Phys. Chem. Chem. Phys.* **2016**, *18*, 5981–5994.

S9. Frisch, M. J.; Trucks, G. W.; Schlegel, H. B.; Scuseria, G. E.; Robb, M. A.; Cheeseman, J. R.; Scalmani, G.; Barone, V.; Petersson, G. A.; Nakatsuji, H.; Li, X.; Caricato, M.; Marenich, A.; Bloino, J.; Janesko, B. G.; Gomperts, R.; Mennucci, B.; Hratchian, H. P.; Ortiz, J. V.; Izmaylov, A. F.; Sonnenberg, J. L.; Williams-Young, D.; Ding, F.; Lipparini, F.; Egidi, F.; Goings, J.; Peng, B.; Petrone, A.; Henderson, T.; Ranasinghe, D.; Zakrzewski, V. G.; Gao, J.; Rega, N.; Zheng, G.; Liang, W.; Hada, M.; Ehara, M.; Toyota, K.; Fukuda, R.; Hasegawa, J.; Ishida, M.; Nakajima, T.; Honda, Y.; Kitao, O.; Nakai, H.; Vreven, T.; Throssell, K.; Montgomery, J. A.; Peralta, J. E.; Ogliaro, F.; Bearpark, M.; Heyd, J. J.; Brothers, E.; Kudin, K. N.; Staroverov, V. N.; Keith, T.; Kobayashi, R.; Normand, J.; Raghavachari, K.; Rendell, A.; Burant, J. C.; Iyengar, S. S.; Tomasi, J.; Cossi, M.; Millam, J. M.; Klene, M.; Adamo, C.; Cammi, R.; Ochterski, J. W.; Martin, R. L.; Morokuma, K.; Farkas, O.; Foresman, J. B.; Fox, D. J. Gaussian 09. Gaussian, Inc.: Wallingford CT 2009.

S10. Stoll, S.; Schweiger, A. EasySpin, a Comprehensive Software Package for Spectral Simulation and Analysis in EPR. *J. Magn. Reson.* **2006**, *178*, 42–55.

S11. Quaranta, A.; Qu, H.; Vencel, T.; Zhang, Y.; Leibl, W.; Leach, S.; Bensasson, R. V. Photophysical Properties in Aqueous Solutions of C60 Embedded in 2:1 γ-Cyclodextrin/[60]Fullerene Inclusion Complexes. *Chem. Phys. Lett.* **2014**, *614*, 234–237.

S12. Strelnikov, A. A.; Konev, A. S.; Levin, O. V.; Khlebnikov, A. F.; Iwasaki, A.; Yamanouchi, K.; Tkachenko, N. V. Switching Competition between Electron and Energy Transfers in Porphyrin–Fullerene Dyads. *J. Phys. Chem. B* **2020**, *124*, 10899–10912.

S13. Becke D. Density-functional thermochemistry. III. The role of exact exchange, *J. Chem. Phys*. **1996**, *98*, 5648-5652.

S14. Grimme, S.; Ehrlich S.; Goerigk L. Effect of the damping function in dispersion corrected density functional theory," *J. Comp. Chem*. **2011**, *32*, 1456-1465.

S15. Schleyer, P. v. R.; Maerker, C.; Dransfeld, A.; Jiao, H.; van Eikema Hommes, N. J. R. Nucleus-Independent Chemical Shifts: A Simple and Efficient Aromaticity Probe. *J. Am. Chem. Soc*. **1996**, *118*, 6317–6318.

S16. Cyrañski, M. K.; Krygowski, T. M.; Wisiorowski, M.; van Eikema Hommes, N. J. R.; Schleyer, P. v. R. Global and Local Aromaticity in Porphyrins: An Analysis Based on Molecular Geometries and Nucleus-Independent Chemical Shifts. *Angew. Chem., Int. Ed*. **1998**, *37*, 177–180.

S17. Chen, Z.; Wannere, C. S.; Corminboeuf, C.; Puchta, R.; Schleyer, P. v. R. Nucleus-Independent Chemical Shifts (NICS) as an Aromaticity Criterion. *Chem. Rev*. **2005**, *105*, 3842–3888.

S18. Fallah-Bagher-Shaidaei, H.; Wannere, C. S.; Corminboeuf, C.; Puchta, R.; Schleyer, P. v. R. Which NICS Aromaticity Index for Planar π Rings Is Best? *Org. Lett*. **2006**, *8*, 863–866.

S19. Stanger, A. NICS – Past and Present. *Eur. J. Org. Chem*. **2020**, 3120–3127.

S20 Stanger, A. Nucleus-Independent Chemical Shifts (NICS): Distance Dependence and Revised Criteria for Aromaticity and Antiaromaticity. *J. Org. Chem*. **2006**, *71*, 883– 893.

S21. Gershoni-Poranne, R.; Stanger, A. The NICS-XY-Scan: Identification of Local and Global Ring Currents in Multi-Ring Systems. *Chem. – Eur. J.* **2014**, *20*, 5673–5688.

S22. Stanger, A. Reexamination of NICSπ,zz: Height Dependence, Off-Center Values, and Integration. *J. Phys. Chem. A* **2019**, *123*, 3922–3927.

S23. Kleinpeter, E.; Klod, S.; Koch, A. Visualization of Through Space NMR Shieldings of Aromatic and Anti-aromatic Molecules and A Simple Means to Compare and Estimate Aromaticity. *J. Mol. Struct*.: THEOCHEM **2007**, *811*, 45–60.
